# Access to Optically Pure β-Hydroxy Esters via Non-Enzymatic Kinetic Resolution by a Planar-Chiral DMAP Catalyst

**DOI:** 10.3390/molecules190914273

**Published:** 2014-09-11

**Authors:** Alba E. Díaz-Álvarez, Laura Mesas-Sánchez, Peter Dinér

**Affiliations:** 1 Department of Chemistry-BMC, Uppsala University, Box 576, Uppsala SE-75123, Sweden; E-Mails: albaestrella.diaz@csic.es (A.E.D.-A.); laura.mesas@kemi.uu.se (L.M.-S.); 2 Department of Chemistry-Organic Chemistry, KTH Royal Institute of Technology, Stockholm SE-100 44, Sweden

**Keywords:** non-enzymatic kinetic resolution, β-hydroxy esters, planar-chiral DMAP catalyst, ferrocenyl catalyst

## Abstract

The development of new approaches to obtain optically pure β-hydroxy esters is an important area in synthetic organic chemistry since they are precursors of other high value compounds. Herein, the kinetic resolution of racemic β-hydroxy esters using a planar-chiral DMAP derivative catalyst is presented. Following this procedure, a range of aromatic β-hydroxy esters was obtained in excellent selectivities (up to *s* = 107) and high enantiomeric excess (up to 99% *ee*). Furthermore, the utility of the present method was demonstrated in the synthesis of (*S*)-3-hydroxy-*N*-methyl-3-phenylpropanamide, a key intermediate for bioactive molecules such as fluoxetine, tomoxetine or nisoxetine, in its enantiomerically pure form.

## 1. Introduction

**Scheme 1:**
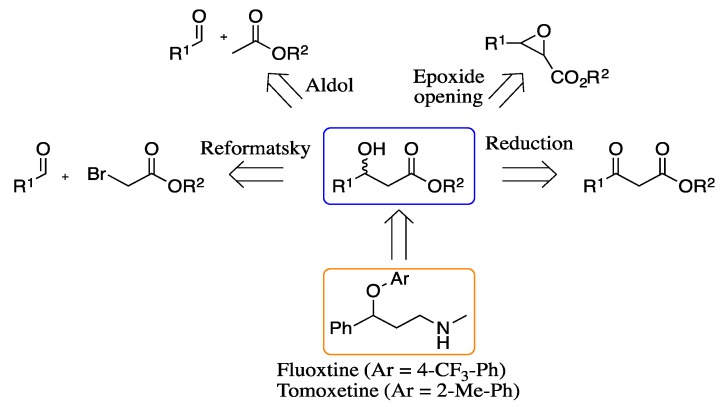
Different asymmetric approaches to β-hydroxy esters.

In the recent decades, the development of methods for asymmetric synthesis of organic molecules has gained increasing importance [1]. As a representative example, the development of approaches to achieve optically pure β-hydroxy esters has emerged as an important issue in synthetic organic chemistry [2], since these chiral derivatives have received special attention due to their importance as intermediates in the synthesis of a variety of important chemicals, such as β-lactams, pheromones and carotenoids [3,4]. In addition, β-hydroxy esters are also precursors to norepinephrine or serotonin reuptake inhibitors that are an important class of drugs. For example, fluoxetine (Prozac), which is formulated as a racemate, is one of the most widely prescribed antidepressants. It has been shown that the different enantiomers display different pharmacological properties [5,6,7] and therefore the enantioselective synthesis of enantiopure compounds is of interest [8,9,10,11,12]. Several routes to produce optically pure 1,3-hydroxy esters have been explored, including asymmetric aldol reaction [13,14,15,16], the Reformatsky reaction [17,18,19,20,21,22], or regioselective epoxide ring opening [23,24,25,26]. The most widely studied method to achieve this kind of derivatives is asymmetric reduction, using either biocatalysts [4,27,28,29,30] or organometallic complexes [31,32,33,34,35,36] (Scheme 1).

**Scheme 2:**
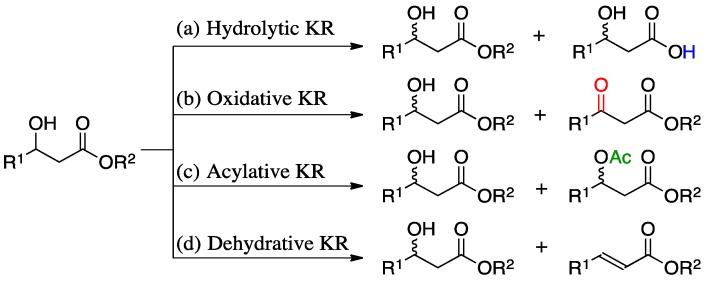
Kinetic resolution alternatives of β-hydroxy esters: (**a**) hydrolytic KR; (**b**) oxidative KR, (**c**) acylative KR and (**d**) dehydrative KR.

In comparison, kinetic resolution (KR) of β-hydroxy esters has been less studied. There are four different kinetic resolution approaches to prepare β-hydroxy esters including: hydrolytic KR [37,38,39,40], oxidative KR [41,42], acylation reactions [43,44,45], and dehydrative KR [46] (Scheme 2a–d).

With the exception of oxidative KR, which takes place in the presence of a palladium complex containing (−)-sparteine ligand [41,42], and the dehydrative KR, in which a chiral aminoalcohol in the presence of a zinc complex dehydrates the chiral alcohol [46], the remaining examples described in bibliography to date are enzymatic processes [37,38,39,40,43,44,45].

In the last decade, several organocatalysts for the enantioselective acylation of alcohols have been developed as an alternative to enzymes [47]. In 1996, Vedejs and Chen described the first example of a chiral DMAP derivative catalyst for the kinetic resolution of secondary aryl alcohols [48,49]. Independently, Fu and co-workers developed the synthesis of planar-chiral ferrocenyl DMAP analogues, e.g., (−)-**1**, which catalyzed the kinetic resolution of secondary aryl [50,51], allylic [52] and propargylic [53] alkyl alcohols with good selectivity factors. In addition, Fu and co-workers recently reported the compatibility of (−)-**1** with a ruthenium-based racemization catalyst representing the first highly selective non-enzymatic dynamic kinetic resolution (DKR) of secondary alcohols [54,55].

**Scheme 3:**
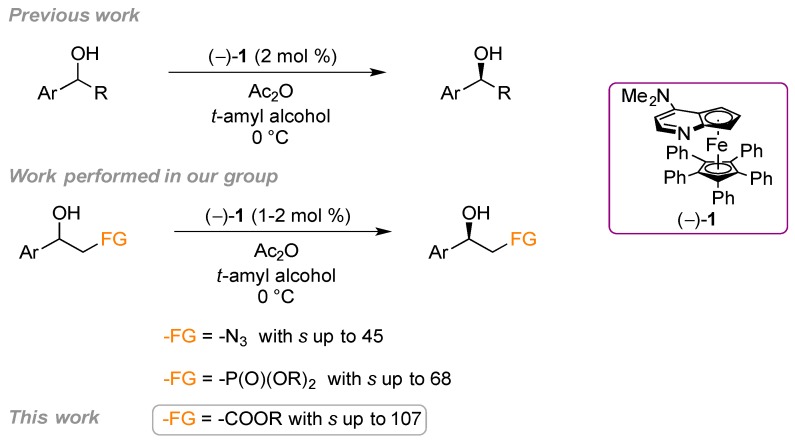
Kinetic resolution of secondary aryl alcohol catalyzed by planar-chiral DMAP derivative (−)-**1**.

The advantages of using the planar-chiral ferrocene DMAP derivative are that both (*S*)-(−)-**1** and (*R*)-(+)-**1** enantiomers are commercially available providing an easy access to both enantiomers of the substrates. Further, the kinetic resolution can be performed with low loading of the planar-chiral DMAP catalyst (1–2 mol %), the catalyst can easily be recovered after the reaction by simple flash chromatography [50,51], and it is possible to combine it with a racemization catalyst for an appealing DKR [54,55]. We have focused our effort in expanding the substrate scope of the KR by (−)-**1** to *sec*-aryl alcohols that contains an additional heteroatom-containing functional group in the alkyl moiety [56,57] (Scheme 3), which includes aromatic β-azido alcohols and β-hydroxy phosphonate alcohols, that are important intermediates for the synthesis of bioactive molecules.

In this study, we wish to present the non-enzymatic kinetic resolution of a variety of β-hydroxy esters with good to excellent selectivities, using the planar-chiral ferrocenyl DMAP catalyst (−)-**1**. Furthermore, the utility of the present method could be demonstrated in the synthesis of the (*S*)-3-hydroxy-*N*-methyl-3-phenylpropanamide, which is a key intermediate for highly selective norepinephrine or serotonin reuptake inhibitor aryloxyphenylpropylamine derivatives, such as fluoxetine, tomoxetine or nisoxetine, in their enantiomerically pure forms.

## 2. Results and Discussion

### 2.1. Substrate Screening

In accordance with previous studies [51,56,57], the kinetic resolution of secondary alcohols catalyzed by the planar-chiral DMAP catalyst (−)-**1** proceeds in an efficient and selective way in *tert*-amyl alcohol and in the presence of acetic anhydride (0.75 equiv.) as acylation agent. Employing these conditions, the influence of the temperature and addition of triethylamine was investigated in the kinetic resolution of *rac*-(**2a**), which was used as a model substrate in order to find the best conditions for the selectivity of the reaction. Using the standard conditions, it was found that the selectivity factor increases when the reaction was performed at lower temperature, but at the expense of the reaction rate with a subsequent increase of the reaction times. Previously, the addition of triethylamine increased the rate of the reaction [50,51,52]. Unfortunately, triethylamine catalyzed the unselective acetylation of the more reactive β-hydroxy esters, resulting in a decrease of the selectivity. For this reason, the rest of the kinetic resolution studies of the β-hydroxy esters were performed at a lower temperature (0 °C) and without addition of base.

Next, we wanted to explore the influence of the bulkiness of the substituent in the β-position to the hydroxyl group on the selectivity of the kinetic resolution. Kinetic resolutions were performed for the substrates (**2a**–**i**) containing either ethyl or the bulkier *tert*-butyl carboxylate group using the standard conditions. The selectivity factor was determined from the enantiomeric excess of the chiral alcohol and the acetylated product after 3 h of reaction (Entries 1 to 9, Table 1).

**Table 1:** Selectivity for the kinetic resolution of β-hydroxy-β-aryl esters (*rac*-2) by (−)-1 ^a^. 
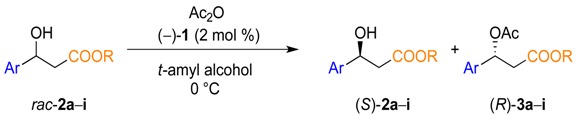

Entry	R	Ar	Time/h	*ee*_ROH_ (%) ^b^	*ee*_ROAc_ (%) ^b^	Conv. (%) ^c^	*s* ^c^
1	Et	Ph (2a)	3	32.4	95.4	25.4	58
2	Et	4-NO_2_-Ph (2b)	3	84.2	92.8	44.6	71
3	Et	4-MeO-Ph (2c)	3	39.8	94.6	29.6	53
4	*tert*-Bu	Ph (2d)	3	28.8	95.2	23.2	54
5	*tert*-Bu	4-NO_2_-Ph (2e)	3	88.4	93.6	48.6	89
6	*tert*-Bu	4-MeO-Ph (2f)	3	39.4	96.0	29.1	72
7	*tert*-Bu	2-Naphthyl (2g)	3	83.4	95.2	46.7	107
8	*tert*-Bu	4-Cl-Ph (2h)	3	75.0	95.2	44.1	92
9	*tert*-Bu	2,6-Cl_2_-Ph (2i)	3	40.4	96.6	29.5	86

^a^: Reaction conditions: *rac*-(**2**) (0.15 mmol), Ac_2_O (0.75 equiv.), (−)-**1** (0.003 mmol, 2 mol %) in 0.6 mL of *tert*-amyl alcohol at 0 °C; ^b^: Determined by HPLC; ^c^: Selectivity and conversion calculated using the following equations: *s* = ln[(1 − *c*)(1 − *ee*_ROH_)]/ln[(1 − *c*)(1 + *ee*_ROH_)] and *c* = *ee*_ROH_/(*ee*_ROH_ + *ee*_ROAc_).

In general, the substrates bearing the bulkier *tert*-butyl group show higher selectivity than the substrates with the ethyl substituents. The largest difference is seen for the substrates containing the electron donating methoxy group or an electron-withdrawing nitro group in the *para* position (Entries 1–6, Table 1). The tendency that kinetic resolutions of substrates with more bulky alkyl substituents lead to higher selectivity is in agreement with the results previously reported by Fu for secondary benzylic alcohols with a bulky aliphatic substituent (the *tert*-butyl compared to the methyl substituent) [51] as well as for secondary β-hydroxyl-β-aryl phosphonates [57].

We also wanted to explore the influence of electronic effects of the substitution on the aromatic ring. The replacement of the phenyl group with an aryl containing an extended π-system led to a higher selectivity (selectivity factor of 107) and a faster reaction rate compared to the parent compound (entry 4 *vs.* entry 7, Table 1). This is consistent with a suggested π–cation interaction between the cationic acylated catalyst and the aromatic substituent, which has previously been suggested for other catalytic systems [58,59,60]. In general, substrates with additional substituents gave a higher selectivity than the parent compound. For example, the compound with an electron donating methoxy group in the *para* position (**2f**) (entry 4 *vs.* entry 6, Table 1) and substrates with electron withdrawing groups in the phenyl ring led to higher selectivity compared to the parent compound. The results follow the previous trend [56,57] that electron-withdrawing substituents in the aromatic ring lead to a higher selectivity. This suggests that the electronic properties affect the interactions between the aromatic ring of the substrate and the acylated catalyst.

The kinetic resolutions were also performed with longer reaction times (24 h) in order to be able to access the remaining alcohol in high enantiomeric excess (Table 2). We were able to isolate all the alcohols **2a**–**f** in high enantiomeric excess (95%–99% *ee*) and in good to moderate yield (23%–45%).

**Table 2:** Substrate screening for the kinetic resolution of β-hydroxy*-*β-aryl esters (*rac*-2) by (−)-1 ^a^. 
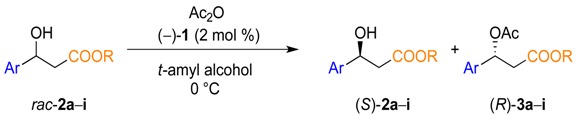

Entry	R	Ar	Conv. (%) ^c^	Yield (%) ^d^	*ee*_ROH_ (%) ^e^
1	Et	Ph (2a)	57	41	99
2	Et	4-NO_2_-Ph (2b)	79	23	99 ^f^
3	Et	4-MeO-Ph (2c)	55	32	99 ^f^
4 ^b^	*tert*-Bu	Ph (2d)	56	39	95
5	*tert*-Bu	4-NO_2_-Ph (2e)	66	33	99 ^f^
6	*tert*-Bu	4-MeO-Ph (2f)	55	41	99
7	*tert*-Bu	2-Naphthyl (2g)	54	45	98
8	*tert*-Bu	4-Cl-Ph (2h)	65	34	99 ^f^
9 ^b^	*tert*-Bu	2,6-Cl_2_-Ph (2i)	63	31	99 ^f^

^a^: Reaction conditions: *rac*-**2** (0.25 mmol), Ac_2_O (0.75 equiv.), (−)-**1** (0.005 mmol, 2 mol %) in 1 mL of *tert*-amyl alcohol at 0 °C, 24 h unless otherwise specified; ^b^: 48 h; ^c^: Conversion calculated using the following equation: *c* = *ee*_ROH_/(*ee*_ROH_ + *ee*_ROAc_); ^d^: Isolated yield base on *rac*-**2**; ^e^: Determined by HPLC; ^f^: A single peak was judged as 99% *ee*.

### 2.2. Assignment of the Absolute Configuration of **2e** and **2i** after KR

**Scheme 4:**
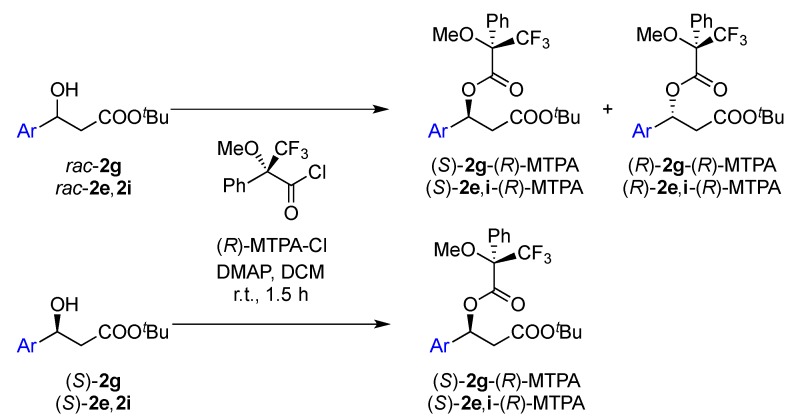
Synthesis of the Mosher’s esters using (*R*)-MTPA chloride as chiral derivatization agent.

The absolute configuration of the optically pure β-hydroxy esters was assigned by comparing the sign of the optical rotation with literature values. Ethyl (*S*)-3-(4-nitrophenyl)-3-hydroxypropanoate (*S*)-**2e** and ethyl (*S*)-3-(2,6-dichlorophenyl)-3-hydroxypropanoate (*S*)-**2i** were the two substrates whose specific rotations are not reported in the literature and had to be determined. In order to confirm the absolute configuration of these two substrates after KR, the secondary alcohol was derivatized with a chiral auxiliary of already known absolute configuration (Scheme 4).

Numerous chiral derivatizing reagents have been described and used to assign the absolute configuration of chiral secondary alcohols [61]. The most common are the aryl-containing carboxylic acids (*R*)-(+) and (*S*)-(−)-α-methoxy-α-trifluoromethylphenylacetic acid (MTPA, Mosher’s reagent; (**4**) [62]. Chadha *et al.*, reported the assignment of the absolute configuration of β-hydroxy esters using a single enantiomer of Mosher’s acid chloride [63,64,65].

**Figure 1:**
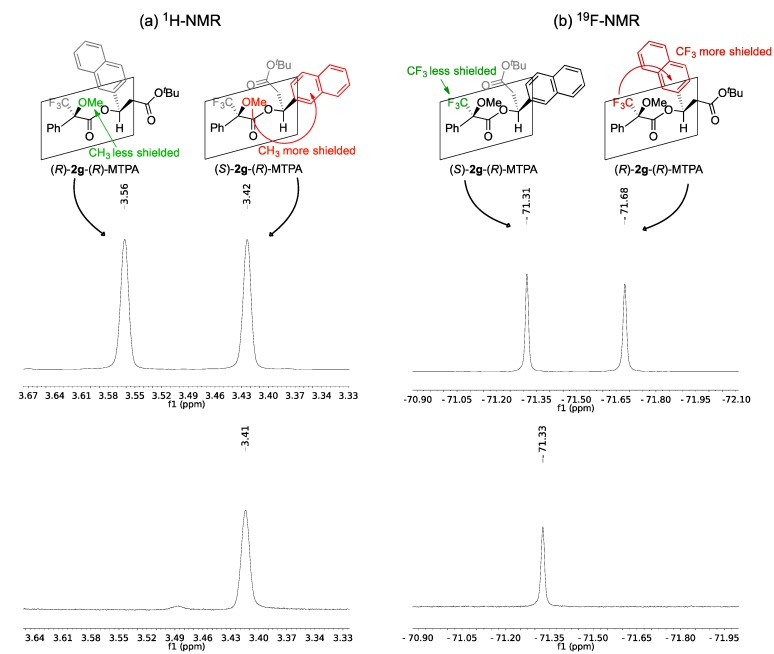
(**a**) ^1^H-NMR of the methoxy region of (*R*)-MTPA esters of *rac*-**2g** (above) and (*R*)-MTPA ester of (*S*)-**2g** (below); (**b**) ^19^F-NMR of (*R*)-MTPA esters of *rac*-**2g** (above) and (*R*)-MTPA ester of (*S*)-**2g** (below).

Firstly, the Mosher’s ester of the racemic mixture and the (*S*)-enantiomer of the substrate *tert*-butyl 3-hydroxy-3-(2-naphthyl)propanoate (**2g**) were prepared and used to validate the method. The ^1^H-NMR spectrum of the mixture of diastereomers formed shows that the chemical shift difference between the methoxy signals is significant enough to be used for the differentiation of the diastereomers (Figure 1a), as reported by Padhi and Chadha [64]. This chemical shift difference in the diastereomer originates from the fact that the methoxy group and the aromatic ring of the β-hydroxy ester are on the same side of the MTPA plane (*i.e*., (*S*)-**2g**-(*R*)-MTPA) and that the signal for the methoxy group protons is shielded due to the diamagnetic effect of the aromatic ring and appears at a lower chemical shift (3.42 ppm) (see Figure 1). The signal from the methoxy protons of the other diastereomer (*i.e*., (*R*)-**2g**-(*R*)-MTPA) appears downfield (3.56 ppm) because they are less shielded. In the same manner, the signals of the CF_3_ group allow the assignment by ^19^F-NMR and eliminate the possibility of overlapping signals (Figure 1b). The signal at −71.68 ppm belongs to the diastereomer that has the CF_3_ group and the aromatic ring on the same side of the MTPA plane (*i.e*., (*R*)-**2g**-(*R*)-MTPA), whereas the signal at −71.31 ppm belongs to the other diastereomer that has these two groups in opposite sides of the plane (*i.e*., (*S*)-**2g**-(*R*)-MTPA) (see Figure 1).

**Figure 2:**
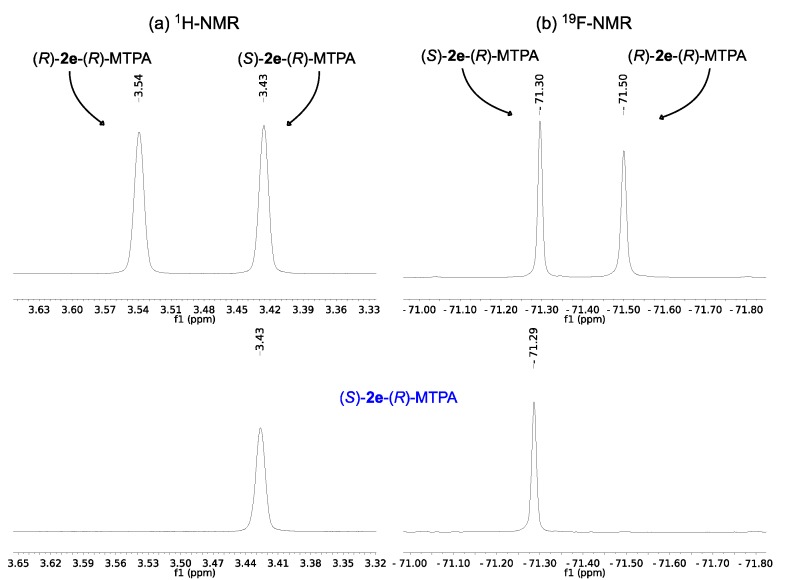
(**a**) ^1^H-NMR of the methoxy region of (*R*)-MTPA esters of *rac*-**2e** (above) and (*R*)-MTPA ester of (*S*)-**2e** (below); (**b**) ^19^F-NMR of (*R*)-MTPA esters of *rac*-**2e** (above) and (*R*)-MTPA ester of (*S*)-**2e** (below).

**Figure 3:**
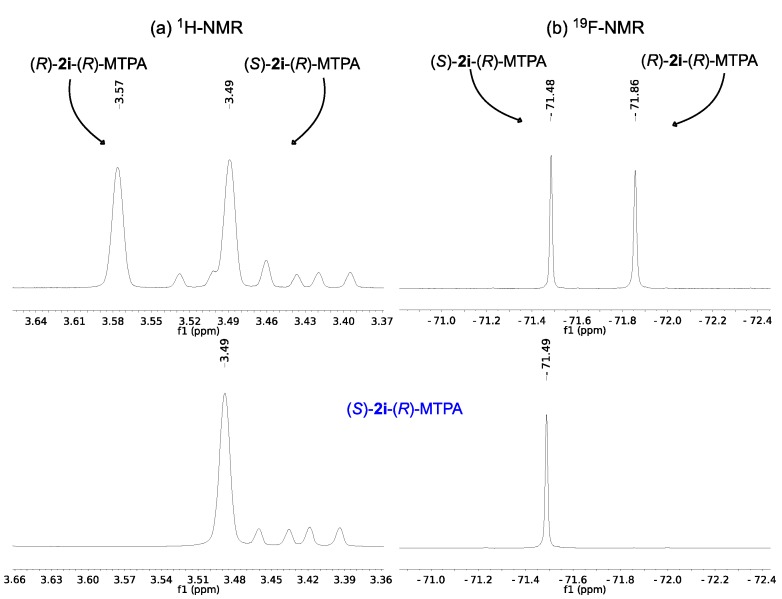
(**a**) ^1^H-NMR of the methoxy region of (*R*)-MTPA esters of *rac*-**2i** (above) and (*R*)-MTPA ester of (*S*)-**2i** (below); (**b**) ^19^F-NMR of (*R*)-MTPA esters of *rac*-**2i** (above) and (*R*)-MTPA ester of (*S*)-**2i** (below).

Therefore, the analysis of the ^1^H and ^19^F-NMR spectrum of the known enantiomer of (*S*)-**2g** allows us to conclude that obtained enantiomer has (*S*)-configuration which is in agreement with the previously reported sign of the optical rotation. The same method was used to determine the absolute configuration of the enantiomerically pure unknown enantiomers of **2e** and **2i** obtained after kinetic resolution. The ^1^H-NMR analysis of the Mosher’s esters (Figure 2a and Figure 3b) shows that the signals of the methoxy protons are shielded (3.43 and 3.49 ppm for the Moshers esters of enantiopure **2e** and **2i** respectively). This supports the fact that the formed diastereomers have the methoxy group and the aromatic ring in the same side of the MTPA plane, *i.e.*, the β-hydroxy esters have (*S*)-configuration.

The same conclusion can be drawn from the ^19^F-NMR spectrum. The fluorine signal obtained belongs to the (*S*)-**2e**-(*R*)-MTPA (−71.29 ppm, Figure 2b) and (*S*)-**2i**-(*R*)-MTPA (−71.49 ppm, Figure 2b) because it appears in the deshielded region and this it indicates that the CF_3_ group and the aromatic ring are not in the same side of the plane as previously observed.

The ^1^H-NMR and ^19^F-NMR analyses confirm that the absolute configuration of ethyl (*S*)-3-(4-nitrophenyl)-3-hydroxypropanoate (*S*)-**2e** and ethyl (*S*)-3-(2,6-dichlorophenyl)-3-hydroxypropanoate (*S*)-**2i** is *S*.

### 2.3. Synthesis of (S)-3-Hydroxy-N-methyl-3-phenylpropanamide

**Scheme 5:**
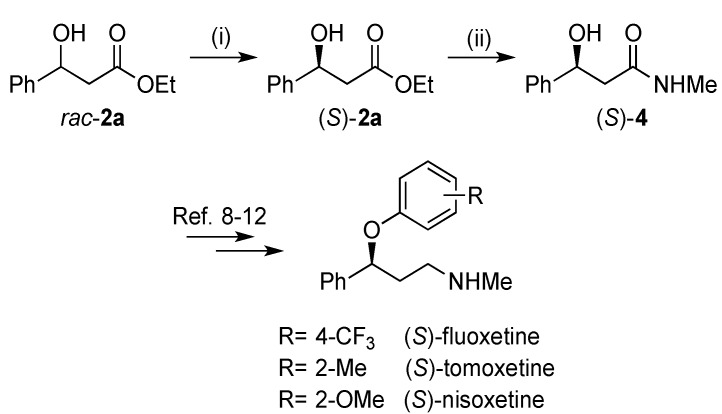
Synthesis of (*S*)-3-hydroxy-*N*-methyl-3-phenylpropanamide.

In order to demonstrate the synthetic applicability of the present method, the synthesis of the enantiomerically pure intermediate (*S*)-3-hydroxy-*N*-methyl-3-phenylpropanamide was carried out (Scheme 5). First, the kinetic resolution of ethyl 3-hydroxy-3-phenylpropanoate, *rac*-**2a** (547.6 mg, 2.8 mmol) was performed, recovering 62% of the (*S*)-enantiomer (32% yield, 99% *ee*). The subsequent treatment of the β-hydroxy esters with aqueous methylamine gave access to the β-hydroxy amide (*S*)-**4** in 85% yield and with retention of the configuration (99% *ee*). The β-hydroxyamide (*S*)-**4** can then be reduced by lithium aluminium hydride in THF to the corresponding amine, followed by an aromatic nucleophilic substitution by 4-chlorobenzotrifluoride using sodium hydride in DMF, in order to yield (*S*)-fluoxetine [8,9,10,11,12].

*Reagents and conditions*: (i) *rac*-(**2a**) (2.8 mmol), Ac_2_O (0.75 equiv.), (−)-**1** (0.005 mmol, 2 mol %) in 1 mL of *tert*-amyl alcohol at 0 °C, 24 h. (ii) MeNH_2_ (40% aq), r.t, 3 h.

## 3. Experimental Section

### 3.1. General Information

Commercially available reagents were purchased from Sigma-Aldrich Co. and used without further purification. Thin layer chromatography (TLC) was performed on ALUGRAM^®^ SIL G/UV_254_ plates (0.2 mm), using UV-light (254 nm) for visualization. Flash column chromatography was performed using Merck silica gel (0.04–0.06 mm). ^1^H and ^13^C{^1^H} NMR spectra were recorded on a Varian Mercury 300 MHz, Varian Unity 400 MHz or Varian Unity 500 MHz spectrometer. The chemical shift values (δ) are given in parts per million (ppm) and are referred to the residual peak of the deuterated solvent used (CDCl_3_). Chemical shifts and literature NMR shifts were used as references in identification and characterization of the optically pure synthesized compounds. Characterization data for these compounds are as follows (copies of the HPLC chromatograms, ^1^H- and ^13^C{^1^H}-NMR spectra are included in the Supplementary data). IR spectra were recorded on a PerkinElmer Spectrum One (ATR Technique). High-resolution mass spectra were recorded by Aleh Yahorau, Department of Pharmaceutical Biosciences, Uppsala University, Sweden.

### 3.2. Preparation of the Racemic Substrates

#### 3.2.1. General Procedure

The racemic substrates **2a**–**c** and **4a**–**f** were prepared following the procedure described by Xu and Yuan [43]. The racemic acetates **3a**–**c** and **5a**–**f** were prepared, as a references for the measurement of the HPLC retention times, by routine acetylation of the corresponding alcohols in dichloromethane at r.t. in the presence of catalytic amounts of DMAP and triethylamine. Chemical shifts and literature NMR shifts were used as references in identification and characterization of the synthesized compounds [43,66,67,68,69,70,71]. For the new substrates (**4b** and **4f**) the IR and HRMS data are included in the characterization data.

#### 3.2.2. Characterization Data

*Ethyl 3-hydroxy-3-phenylpropanoate* (**2a**) [43]

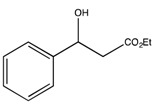

^1^H-NMR (CDCl_3_) δ 7.28–7.17 (m, 5H, Ar-H), 5.04 (dd, *J* = 7.8, 4.2 Hz, 1H, CH), 4.10 (m, *J* = 6.9 Hz, 2H, CH_2_), 3.22 (bs, 1H, OH), 2.64-2.50 (m, 2H, CH_2_), and 1.17 (t, *J* = 6.9 Hz, 3H, CH_3_). ^13^C{^1^H}-NMR (CDCl_3_) δ 172.6, 142.8, 128.8 (2C), 128.0, 125.9 (2C), 70.6, 61.1, 43.6 and 14.4.

*Ethyl 3-hydroxy-3-(4-nitrophenyl)propionate* (**2b**) [43] 
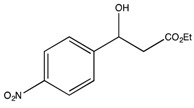

^1^H-NMR (CDCl_3_) δ 7.87 (d, 2H, Ar-H), 7.46 (d, 2H, Ar-H), 5.25 (dd, *J* = 7.8, 4.2 Hz, 1H, CH), 4.29–4.11 (m, 2H, CH_2_), 3.62 (bs, 1H, OH), 2.71 (q, *J* = 6.9 Hz, 2H, CH_2_) and 1.24 (t, *J* = 6.9 Hz, 3H, CH_3_). ^13^C{^1^H}-NMR (CDCl_3_) δ 172.0, 149.9, 147.4, 126.2 (2C), 123.9 (2C), 69.2, 61.3, 43.1 and 14.1.

*Ethyl 3-hydroxy-3-(4-metoxyphenyl)propionate* (**2c**) [66] 
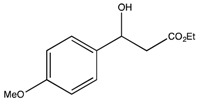

^1^H-NMR (CDCl_3_) δ. 7.29 (d, 2H, *J* = 7.5 Hz, Ar-H), 6.86 (d, 2H, *J* = 7.5 Hz, Ar-H), 5.08 (dd, *J* = 8.8, 4.0 Hz, 1H, CH), 4.19–4.09 (m, 2H, CH_2_), 3.80 (s, 3H, OCH_3_), 2.76–2.69 (m, 2H, CH_2_) and 1.27 (t, *J* = 6.9 Hz, 3H, CH_3_). ^13^C{^1^H}-NMR (CDCl_3_) δ 172.3, 159.1, 134.8, 126.9 (2C), 113.8 (2C), 69.9, 60.7, 55.2, 43.3 and 14.1.

*t-Butyl 3-hydroxy-3-phenylpropionate* (**2d**) [67] 
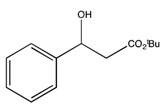

^1^H-NMR (CDCl_3_) δ 7.39–7.22 (m, 5H, Ar–H), 5.07 (dd, 1H, *J* = 7.8, 4.2 Hz, CH), 3.42 (bs, 1H, OH), 2.67 (dd, 1H, *J* = 16.0, 4.8 Hz, CH2), 2.61 (dd, 1H, *J* = 16.0, 4.8 Hz, CH_2_) and 1.43 (s, 9H, 3 CH_3_). ^13^C{1H}-NMR (CDCl_3_) δ 171.9, 142.6, 128.4 (2C), 127.6, 125.7 (2C), 81.5, 70.4, 44.3 and 28.0.

*t-Butyl 3-hydroxy-3-(4-nitrophenyl)propionate* (**2e**) [68] 
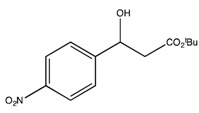

^1^H-NMR (CDCl_3_) δ 8.20 (d, 2H, *J* = 8.1 Hz, Ar-H), 7.55 (d, 2H, *J* = 8.1 Hz, Ar-H), 5.18 (dd, *J* = 8.3, 4.1 Hz, 1H, CH), 2.73–2.57 (m, 2H, CH_2_) and 1.49 (s, 9H, 3 CH_3_). ^13^C{^1^H}-NMR (CDCl_3_) δ 171.3, 150.0, 147.3, 126.5 (2C), 123.6 (2C), 82.1, 69.4, 43.8 and 28.0. IR (neat): *v*(cm^−1^) 3438, 2941, 2977, 2903, 1700, 1510, 1343, 1148, 843. HRMS (ESI, *m/z*) Calcd for C_13_H_18_NO_5_^+^ [M+H^+^]: 268.1185, found: 268.1189.

*t-Butyl 3-hydroxy-3-(4-methoxyphenyl)propionate* (**2f**) [69] 
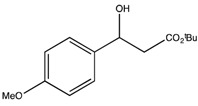

^1^H-NMR (CDCl_3_) δ 7.29 (d, 2H, *J* = 7.5 Hz, Ar-H), 6.86 (d, 2H, *J* = 7.5 Hz, Ar-H), 5.01 (dd, *J* = 8.8, 4.0 Hz, 1H, CH), 3.78 (s, 3H, OCH_3_), 3.15 (bs, 1H, OH), 2.71–2.54 (m, 2H, CH_2_) and 1.44 (s, 9H, 3 CH_3_). ^13^C{^1^H}-NMR (CDCl_3_) δ 171.9, 159.1, 134.9, 127.0 (2C), 113.8 (2C), 81.4, 70.0, 55.2, 44.3, and 28.0.

*t-Butyl 3-hydroxy-3-(2-naphthyl)propionate* (**2g**) [70] 
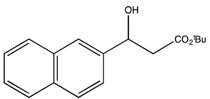

^1^H-NMR (CDCl_3_) δ 7.85–7.81 (m, 4H, Ar-H), 7.50–7.26 (m, 3H, Ar-H), 5.26 (dd, *J* = 7.3, 5.5 Hz, 1H, CH), 2.76–2.74 (m, 2H, CH_2_) and 1.46 (s, 9H, 3 CH_3_).^ 13^C{^1^H}-NMR (CDCl_3_) δ 171.9, 140.1, 133.3, 133.0, 128.3, 128.0, 127.7, 126.1, 125.9, 124.5, 123.9, 81.6, 70.5, 44.3 and 28.1.

*t-Butyl 3-hydroxy-3-(4-chloro)propionate* (**2h**) [69] 
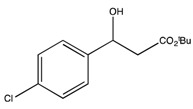

^1^H-NMR (CDCl_3_) δ 7.34–7.31 (m, 4H, Ar-H), 5.09–5.05 (m, 1H, CH), 2.63–2.60 (m, 2H, CH_2_) and 1.45 (s, 9H, 3 CH_3_). ^13^C{1H}-NMR (CDCl3) δ 171.7, 141.1, 133.3, 128.6 (2C), 127.1 (2C), 81.7, 69.7, 44.1 and 28.0.

*t-Butyl 3-hydroxy-3-(2',6'-dichlorophenyl) propionate* (**2i**)

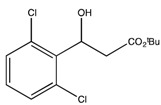

^1^H-NMR (CDCl_3_) δ 7.32–7.12 (m, 3H, Ar-H), 5.90 (dd, 1H, *J* = 10.5, *J* = 4.2 Hz, 1H, CH), 3.23–2.62 (m, 2H, CH_2_) and 1.46 (s, 9H, 3 CH_3_). ^13^C{^1^H}-NMR (CDCl_3_) δ 170.5, 136.2, 134.6, 129.4 (2C), 129.2 (2C), 81.3, 68.2, 40.7 and 28.0. IR (neat): *v*(cm^−1^) 3514, 2987, 2938, 1700, 1561, 1144, 766. HRMS (ESI, *m/z*) Calcd for C_13_H_17_Cl_2_O_3_^+^ [M+H^+^]: 291.0555, found: 291.0551.

### 3.3. General Procedure for the Kinetic Resolution

#### 3.3.1. Selectivity Factor for the KR of **2a**–**i** after 3 h

Catalyst (−)-**1** (2.0 mg, 0.003 mmol), β-hydroxy ester **2a**–**i** (0.15 mmol) and *t*-amyl alcohol (0.6 mL) were sequentially added to a vial. The vial was capped and stirred at room temperature to help dissolve the catalyst. The reaction mixture was cooled to 0 °C in an ice-water bath and stirred for 15 min. Then, acetic anhydride (11 μL, 0.11 mmol) was added. After 3 h, a sample (0.2–0.3 mL) was quenched by the addition of methanol. The resulting solution was filtered through a short plug of silica using ether as eluent and then it was concentrated. The enantiomeric excess of the unreactive alcohol and the acetate were determined by HPLC (Table 1) using the appropriate chiral column and conditions. The chromatograms are attached in Supplementary section 1.

The selectivity *s* was calculated using the Equation (1), where *C* is the conversion (calculated using the Equation (2), *ee*_ROH_ and *ee*_ROAc_ are the enantiomeric excess for the unreactive alcohol and the resulting acetate respectively determined by HPLC [47]:

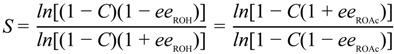
(1)

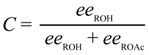
(2)


#### 3.3.2. Synthesis of Optically Pure Alcohols

Catalyst (−)-**1** (3.3 mg, 0.005 mmol), β-hydroxy ester **2a**–**i** (0.25 mmol) and *t*-amyl alcohol (1.0 mL) were sequentially added to a vial. The vial was capped and stirred at room temperature to help dissolve the catalyst. The reaction mixture was cooled to 0 °C, and then acetic anhydride (18 μL, 0.19 mmol) was added. After the appropriate amount of time, the reaction mixture was quenched by the addition of a large excess of methanol. The resulting solution was concentrated, and the unreactive alcohol, the acetate and the catalyst were separated by flash chromatography using increasing polarity mixtures of pentane/ethyl acetate as eluent. The enantiomeric excess of the unreactive alcohol and the acetate were determined by HPLC (Table 2). The chromatograms are attached in Supplementary section 2. Chemical shifts and literature NMR shifts were used as references in identification and characterization of the optically pure synthesized compounds **2a**–**i**. The ^1^H and ^13^C{^1^H}-NMR spectra are attached in Supplementary section 3.

*Ethyl (S)-3-hydroxy-3-phenylpropanoate* ((*S*)-**2a**) [43]. Colorless oil (21.8 mg, 41% yield); *ee* 95%, Kromasil 5-CellCoat, *n*-hexane/*i*-PrOH = 95:05, 0.5 mL/min, 220 nm, t_R_[(*S*)/(*R*)] = 20.5/28.5 min; 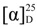: −45.3 (*c* 3.7, CDCl_3_) (Lit. [71] [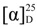: = −46.5 (*c* 1.04, CHCl_3_)). ^1^H-NMR (CDCl_3_) δ 7.28–7.17 (m, 5H, Ar*H*), 5.04 (dd, *J* = 7.8, 4.2 Hz, 1H, C*H*), 4.10 (m, *J* = 6.9 Hz, 2H, C*H*_2_), 3.22 (bs, 1H, O*H*), 2.64–2.50 (m, 2H, C*H*_2_), and 1.17 (t, *J* = 6.9 Hz, 3H, C*H*_3_). ^13^C{^1^H}-NMR (CDCl_3_) δ 172.6, 142.8, 128.8 (2C), 128.0, 125.9 (2C), 70.6, 61.1, 43.6 and 14.4.

*Ethyl (S)-3-hydroxy-3-(4-nitrophenyl)propanoate* ((*S*)-**2b**) [43]. Colorless oil (13.8 mg, 23% yield); *ee* 99%, Chiralpack AD, *n*-hexane/*i*-PrOH = 90:10, 0.5 mL/min, 220 nm, t_R_[(*S*)/(*R*)] = 26.6/28.4 min. 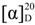: −58.0 (*c* 0.23, CHCl_3_) (Lit. [64] 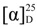: −59.5 (*c* 1.5, CHCl_3_)). ^1^H-NMR (CDCl_3_) δ 7.87 (d, 2H, Ar*H*), 7.46 (d, 2H, Ar*H*), 5.25 (dd, *J* = 7.8, 4.2 Hz, 1H, C*H*), 4.29–4.11 (m, 2H, C*H*_2_), 3.62 (bs, 1H, O*H*), 2.71 (q, *J* = 6.9 Hz, 2H, C*H*_2_) and 1.24 (t, *J* = 6.9 Hz, 3H, C*H*_3_). ^13^C{^1^H}-NMR (CDCl_3_) δ 172.0, 149.9, 147.4, 126.2 (2C), 123.9 (2C), 69.2, 61.3, 43.1 and 14.1.

*Ethyl (S)-3-hydroxy-3-(4-methoxyphenyl)propanoate* ((*S*)-**2c**) [66]. Yellow oil (18.0 mg, 32% yield); *ee* 99%, Reprosil chiral NR, *n*-hexane/*i*-PrOH = 80:20, 0.5 mL/min, 220 nm, t_R_[(*S*)/(*R*)] = 15.9/18.7 min. 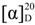: −45.9 (*c* 0.75, CHCl_3_) (Lit. [71] 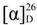: −28.6 (*c* 1, CHCl_3_)). ^1^H-NMR (CDCl_3_) δ 7.29 (d, 2H, *J* = 7.5 Hz, Ar*H*), 6.86 (d, 2H, *J* = 7.5 Hz, Ar*H*), 5.08 (dd, *J* = 8.8, 4.0 Hz, 1H, C*H*), 4.19–4.09 (m, 2H, C*H*_2_), 3.80 (s, 3H, OC*H*_3_), 2.76–2.69 (m, 2H, C*H*_2_) and 1.27 (t, *J* = 6.9 Hz, 3H, C*H*_3_). ^13^C{^1^H}-NMR (CDCl_3_) δ 172.3, 159.1, 134.8, 126.9 (2C), 113.8 (2C), 69.9, 60.7, 55.2, 43.3 and 14.1.

*tert-Butyl (S)-3-hydroxy-3-phenylpropanoate* ((*S*)-**2d**) [67]. Pale yellow oil (21.6 mg, 39% yield); *ee* 95%, Reprosil Chiral NR, *n*-hexane/*i*-PrOH = 99:01, 1.0 mL/min, 220 nm, t_R_[(*S*)/(*R*)] = 11.4/15.6 min. 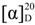: −85.0 (*c* 0.1, CHCl_3_) (Lit. [71] 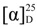: −37.7 (*c* 1.2, CHCl_3_)). ^1^H-NMR (CDCl_3_) δ 7.39–7.22 (m, 5H, Ar*H*), 5.07 (dd, 1H, *J* = 7.8, 4.2 Hz, C*H*), 3.42 (bs, 1H, O*H*), 2.67 (dd, 1H, *J* = 16.0, 4.8 Hz, C*H*_2_), 2.61 (dd, 1H, *J* = 16.0, 4.8 Hz, C*H*_2_) and 1.43 (s, 9H, 3 C*H*_3_). ^13^C{1H}-NMR (CDCl_3_) δ 171.9, 142.6, 128.4 (2C), 127.6, 125.7 (2C), 81.5, 70.4, 44.3 and 28.0.

*tert-Butyl (S)-3-hydroxy-3-(4-nitrophenyl)propanoate* ((*S*)-**2e**) [68]. Yellow oil (18.0 mg, 32% yield); *ee* 99%, Reprosil Chiral NR, *n*-hexane/*i*-PrOH = 90:10, 0.5 mL/min, 220 nm, t_R_[(*S*)/(*R*)] = 14.5/15.5 min. 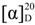: −31.4 (*c* 0.19, CHCl_3_). ^1^H-NMR (CDCl_3_) δ 8.20 (d, 2H, *J* = 8.1 Hz, Ar*H*), 7.55 (d, 2H, *J* = 8.1 Hz, Ar*H*), 5.18 (dd, *J* = 8.3, 4.1 Hz, 1H, C*H*), 2.73–2.57 (m, 2H, C*H*_2_) and 1.49 (s, 9H, 3 C*H*_3_). ^13^C{^1^H}-NMR (CDCl_3_) δ 171.3, 150.0, 147.3, 126.5 (2C), 123.6 (2C), 82.1, 69.4, 43.8 and 28.0. IR (neat): *v*(cm^−1^) 3438, 2941, 2977, 2903, 1700, 1510, 1343, 1148, 843. HRMS (ESI, *m/z*) Calcd for C_13_H_18_NO_5_^+^ [M+H^+^]: 268.1185, found: 268.1189.

*tert-Butyl (S)-3-hydroxy-3-(4-methoxyphenyl)propanoate* ((*S*)-**2f**) [69]. Yellow oil (26.0 mg, 41% yield); *ee* 99% after acetylation with DMAP, Chiralpak AD, *n*-hexane/*i*-PrOH = 90:10, 0.5 mL/min, 220 nm, t_R_[(*S*)/(*R*)] = 24.8/11.9 min. 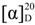: −34.5 (*c* 1.6, CHCl_3_) (Lit. value for the (*R*)-enantiomer [46] 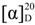: +28.0 (*c* 0.15, CHCl_3_)). ^1^H-NMR (CDCl_3_) δ 7.29 (d, 2H, *J* = 7.5 Hz, Ar*H*), 6.86 (d, 2H, *J* = 7.5 Hz, Ar*H*), 5.01 (dd, *J* = 8.8, 4.0 Hz, 1H, C*H*), 3.78 (s, 3H, OC*H*_3_), 3.15 (bs, 1H, O*H*), 2.71–2.54 (m, 2H, C*H*_2_) and 1.44 (s, 9H, 3 C*H*_3_). ^13^C{^1^H}-NMR (CDCl_3_) δ 171.9, 159.1, 134.9, 127.0 (2C), 113.8 (2C), 81.4, 70.0, 55.2, 44.3, and 28.0.

*tert-Butyl (S)-3-hydroxy-3-(2-naphthyl)propanoate* ((*S*)-**2g**) [70]. Yellow oil (30.3 mg, 45% yield); *ee* 96%, Kromasil 5-CellCoat, *n*-hexane/*i*-PrOH = 99:01, 1.0 mL/min, 220 nm, t_R_[(*S*)/(*R*)] = 26.4/29.6 min. 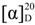: −36.9 (*c* 0.42, CHCl_3_) (Lit. [21] 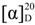: −24.8 (*c* 1.1, CHCl_3_)). ^1^H-NMR (CDCl_3_) δ 7.85–7.81 (m, 4H, Ar*H*), 7.50–7.26 (m, 3H, Ar*H*), 5.26 (dd, *J* = 7.3, 5.5 Hz, 1H, C*H*), 2.76–2.74 (m, 2H, C*H*_2_) and 1.46 (s, 9H, 3 C*H*_3_). ^13^C{^1^H}-NMR (CDCl_3_) δ 171.9, 140.1, 133.3, 133.0, 128.3, 128.0, 127.7, 126.1, 125.9, 124.5, 123.9, 81.6, 70.5, 44.3 and 28.1.

*tert-Butyl (S)-3-(4-chlorophenyl) 3-hydroxypropanoate* ((*S*)-**2h**) [69]. Colorless oil (22.0 mg, 33% yield); *ee* 99%, Reprosil Chiral NR, *n*-hexane/*i*-PrOH = 90:10, 0.5 mL/min, 220 nm, t_R_[(*S*)/(*R*)] = 10.7/12.2 min. 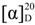: −34.1 (*c* 1.9, CHCl_3_) (Lit. [21] 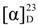: −25.4 (*c* 2.0, CHCl_3_)). ^1^H-NMR (CDCl_3_) δ 7.34–7.31 (m, 4H, Ar*H*), 5.09–5.05 (m, 1H, C*H*), 2.63–2.60 (m, 2H, C*H*_2_) and 1.45 (s, 9H, 3 C*H*_3_). ^13^C{1H}-NMR (CDCl3) δ 171.7, 141.1, 133.3, 128.6 (2C), 127.1 (2C), 81.7, 69.7, 44.1 and 28.0.

*tert-Butyl (S)-3-(2*,*6-dichlorophenyl) 3-hydroxypropanoate* ((*S*)-**2i**). Colorless oil (23.0 mg, 31% yield); *ee* 99%, Kromasil 5-CellCoat, *n*-hexane/*i*-PrOH = 99.5:0.5, 1.0 mL/min, 220 nm, t_R_[(*S*)/(*R*)] = 10.7/16.1 min. 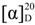: +21.8 (*c* 2.0, CHCl_3_). ^1^H-NMR (CDCl_3_) δ 7.32–7.12 (m, 3H, Ar*H*), 5.90 (dd, 1H, *J* = 10.5, *J* = 4.2 Hz, 1H, C*H*), 3.23–2.62 (m, 2H, C*H*_2_) and 1.46 (s, 9H, 3 C*H*_3_).^ 13^C{^1^H}-NMR (CDCl_3_) δ 170.5, 136.2, 134.6, 129.4 (2C), 129.2 (2C), 81.3, 68.2, 40.7 and 28.0. IR (neat): *v*(cm^−1^) 3514, 2987, 2938, 1700, 1561, 1144, 766. HRMS (ESI, *m/z*) Calcd for C_13_H_17_Cl_2_O_3_^+^ [M+H^+^]: 291.0555, found: 291.0551.

#### 3.3.3. Methods Used to Determine Enantiomeric Excess

The enantiomeric separations of β-hydroxy esters **2a**–**i** were performed by high performance liquid chromatography (HPLC) with a Young Lin 9100 instrument using the appropriate chiral column at 25 °C with *n*-hexane and isopropanol as eluents. The enantiomeric separation of **2f** was achieved converting the alcohol to the corresponding acetate using DMAP and triethylamine in DCM. The selectivity (*S*)-factors were calculated with the equation: *S* = ln[(1 − c)(1 − *ee*_ROH_)]/ln[(1 − c)(1 + *ee*_ROH_)].

### 3.4. Synthesis of (S)-3-Hydroxy-N-methyl-3-phenylpropanamide ((S)-**4**)

Catalyst (−)-**1** (37 mg, 0.0056 mmol), racemic substrate **2a** (2.8 mmol) and *tert*-amyl alcohol (11 mL) were sequentially added to a vial. The vial was capped and stirred at room temperature to help dissolve the catalyst. The reaction mixture was cooled to 0 °C, and then acetic anhydride (200 μL, 2.1 mmol) was added. After 24 h, the reaction mixture was quenched by the addition of a large excess of methanol. The resulting solution was concentrated, and the unreactive alcohol was separated from the acetate and the catalyst by flash chromatography using increasing polarity mixtures of pentane/ethyl acetate as eluent (175 mg, 32% yield, 99% *ee*). The β-hydroxy ester (*S*)-**2a** was treated with a 40% aqueous solution of methylamine (1.56 mL, 18 mmol) at r.t. during 3 h. Beige solid was obtained (137.8 mg, 85.3% yield, 99% *ee*). Chemical shifts and literature NMR shifts were used as references in identification and characterization of the optically pure synthesized compound (*S*)-**4** [36]. Beige solid (138 mg, 85% yield); *ee* 99%, Chiralpak AD, *n*-hexane/*i*-PrOH = 98:02, 1.0 mL/min, 220 nm, t_R_[(*R*)/(*S*)] = 41/51 min. 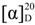: −26.7 (*c* 1.0, CH_3_OH) (Lit. [36] 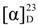: −26.2 (*c* 1.25, CH_3_OH)). ^1^H-NMR (CDCl_3_) δ 7.49–7.14 (m, 5H, Ar*H*), 5.82 (br s, 1H, N*H*), 5.16-5.05 (m, 1H, C*H*OH), 2.82 (d, *J* = 4.5 Hz, 3H, NHC*H*_3_), 2.59–2.52 (m, 2H, C*H*_2_). ^13^C{1H}-NMR (CDCl_3_) δ 172.56, 143.16, 128.67, 127.84, 125.71, 71.07, 44.72, 26.37.

## 4. Conclusions

In summary, we have demonstrated that the planar-chiral DMAP derivative catalyst (−)-**1** catalyzes the kinetic resolution for a range of aromatic β-hydroxy esters with excellent selectivities (selectivity factor up to 107) and high enantiomeric excess (up to 99% *ee*) of the remaining alcohol. To the best of our knowledge, these results represent the first example of the kinetic resolution of this family of substrates employing a chiral DMAP derivative as catalyst.

## Supplementary Materials

Supplementary materials can be accessed at: http://www.mdpi.com/1420-3049/19/9/14273/s1.

## Supplementary Files

Supplementary File 1
