# Supplementary material for: Access to Optically Pure β-Hydroxy Esters via Non-Enzymatic Kinetic Resolution by a Planar-Chiral DMAP Catalyst
Source: Molecules. 2014 Sep 11;19(9):14273–91. doi: 10.3390/molecules190914273 (PMC6270874; doi:10.3390/molecules190914273)

Supplementary Materials

Table of Contents

Supplementary Section 1. HPLC Chromatograms for the Determination of *s* S1
Supplementary Section 2. HPLC Chromatograms of Isolated Compounds S6
Supplementary Section 3. NMR Spectra.. S25

Supplementary Section 1. HPLC Chromatograms for the Determination of *s*

Figure S1. KR of ethyl 3-hydroxy-3-phenylpropanoate, **2a**.

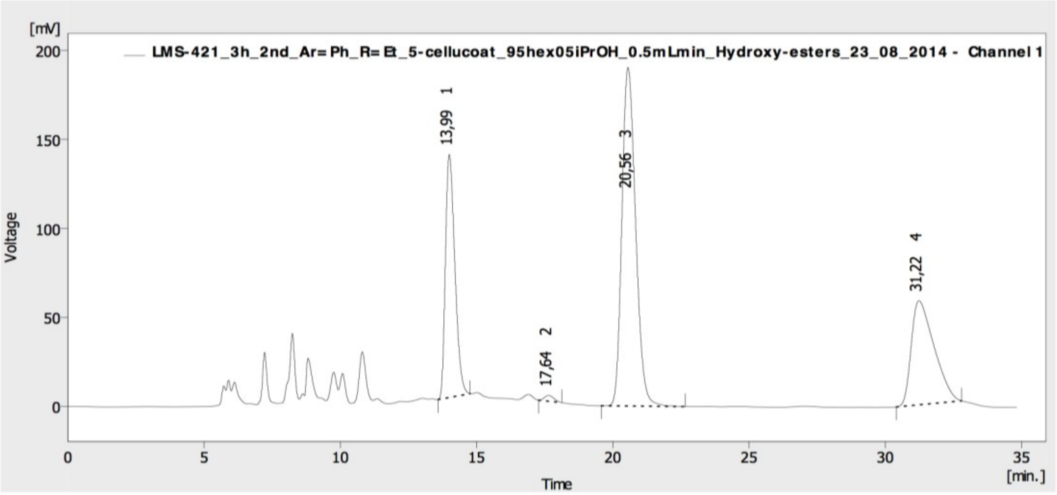

Result Table (Uncal - LMS-421_3h_2nd_Ar=Ph_R=Et_5-cellucoat_95hex05iPrOH_0.5mLmin_Hydroxy-esters_23_08_2014 - Channel 1)

	Reten. Time [min]	Area [mV.s]	Area [%]	W05 [min]	Compound Name	ee
1	13,990	3293,665	24,5	0,39		3,068
2	17,640	79,006	0,6	0,39		1,365
3	20,557	6656,013	49,6	0,54		1,568
4	31,223	3395,518	25,3	0,94		4,375
	Total	13424,202	100,0			10,376

Figure S2. KR of ethyl 3-hydroxy-3-(4-nitrophenyl)propanoate, **2b**.

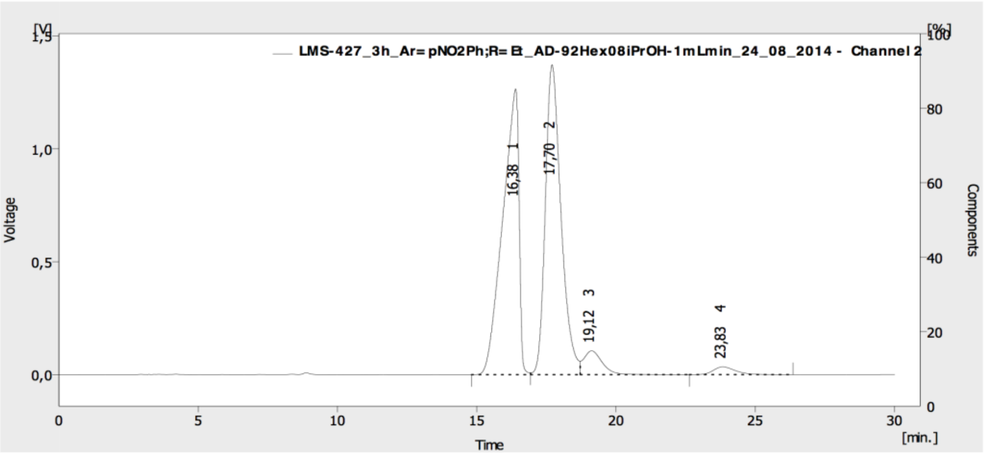

Result Table (Uncal - LMS-427_3h_Ar=pNO2Ph;R=Et_AD-92Hex08iPrOH-1mLmin_24_08_2014 - Channel 2)

	Reten. Time [min]	Area [mV.s]	Height [mV]	Area [%]	Height [%]	W05 [min]	Compound Name
1	16,383	51243,807	1264,494	45,8	45,5	0,65	
2	17,700	53812,242	1371,853	48,1	49,4	0,59	
3	19,117	4926,490	105,661	4,4	3,8	0,80	
4	23,833	1890,957	34,435	1,7	1,2	0,84	
	Total	111873,497	2776,443	100,0	100,0		

Figure S3. KR of ethyl 3-hydroxy-3-(4-metoxyphenyl)propanoate, **2c**.

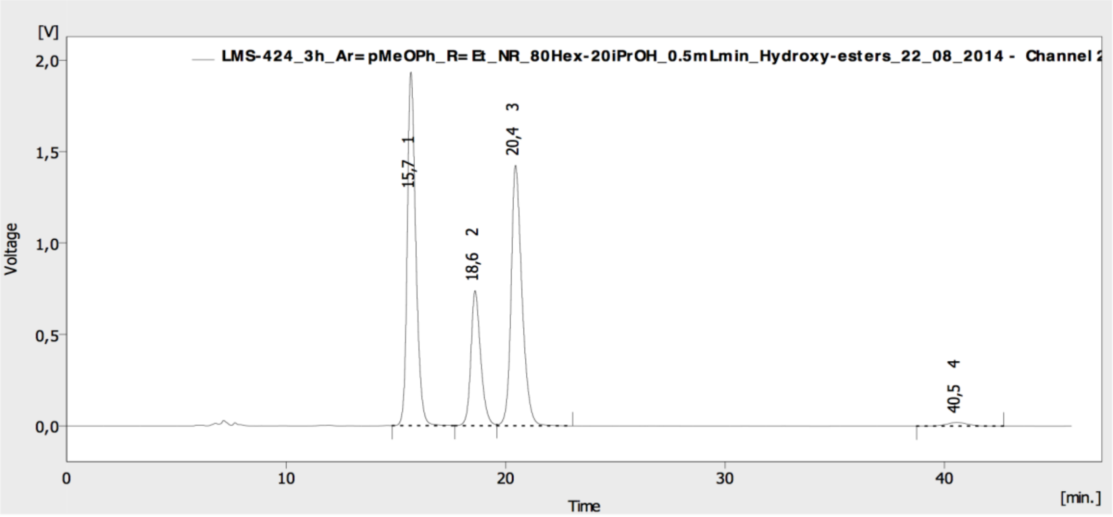

Result Table (Uncal - LMS-424_3h_Ar=pMeOPh_R=Et_NR_80Hex-20iPrOH_0.5mLmin_Hydroxy-esters_22_08_2014 - Channel 2)

	Reten. Time [min]	Area [mV.s]	Area [%]	W05 [min]	Compound Name	ee
1	15,683	52301,355	41,7	0,40		2,139
2	18,600	22866,926	18,2	0,46		2,170
3	20,450	49013,043	39,1	0,51		2,383
4	40,550	1329,088	1,1	0,99		0,380
	Total	125510,412	100,0			7,072

Figure S4. KR of *t*-Butyl 3-hydroxy-3-phenylpropanoate, **2d**.

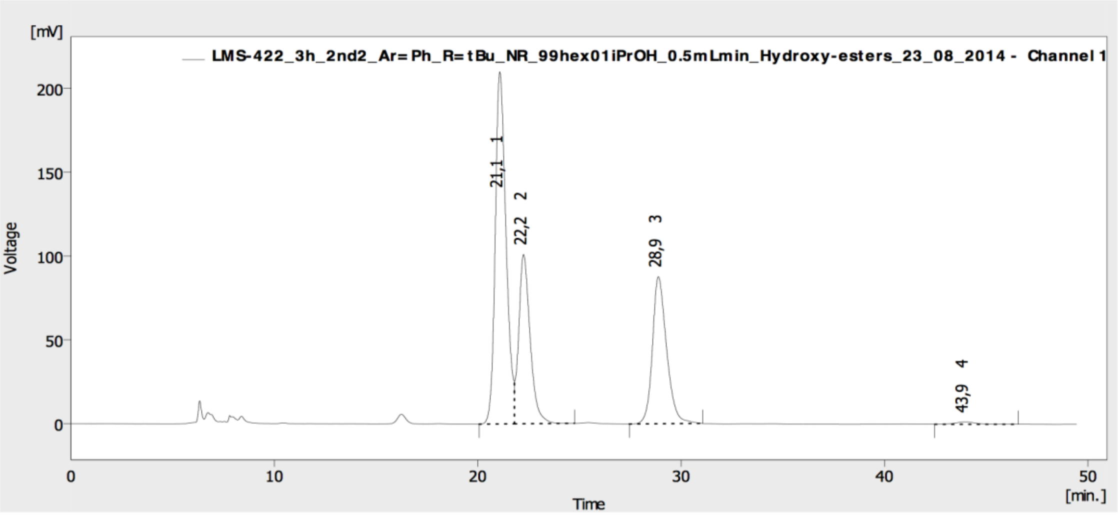

Result Table (Uncal - LMS-422_3h_2nd2_Ar=Ph_R=tBu_NR_99hex01iPrOH_0.5mLmin_Hydroxy-esters_23_08_2014 - Channel 1)

	Reten. Time [min]	Area [mV.s]	Area [%]	W05 [min]	Compound Name	ee
1	21,067	7729,771	48,2	0,56		2,968
2	22,233	3957,831	24,7	0,59		2,670
3	28,867	4261,705	26,6	0,72		1,999
4	43,933	94,821	0,6	1,02		0,230
	Total	16044,127	100,0			7,868

Figure S5. KR of *t*-Butyl 3-hydroxy-3-(4-nitrophenyl)propanoate, **2e**.

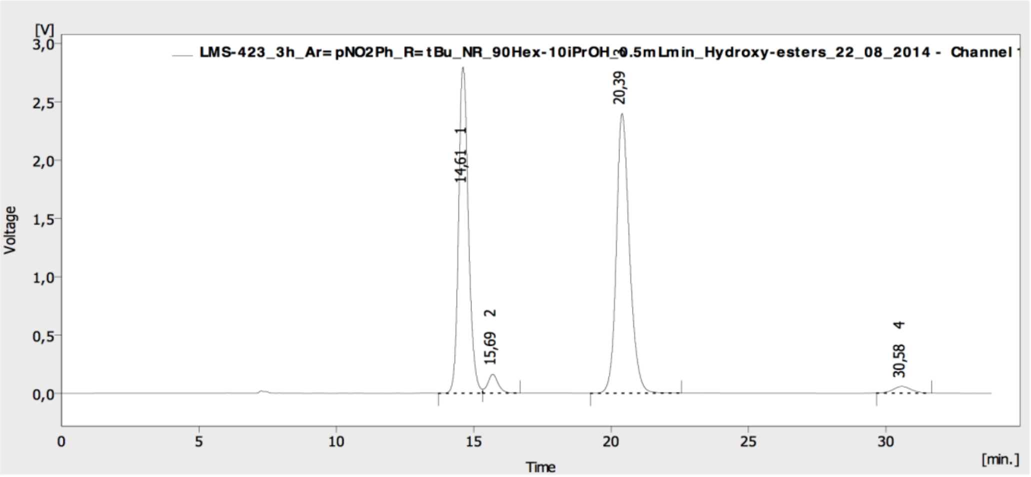

Result Table (Uncal - LMS-423_3h_Ar=pNO2Ph_R=tBu_NR_90Hex-10iPrOH_0.5mLmin_Hydroxy-esters_22_08_2014 - Channel 1)

	Reten. Time [min]	Area [mV.s]	Area [%]	W05 [min]	Compound Name	ee
1	14,610	69711,705	44,5	0,39		1,465
2	15,693	4254,201	2,7	0,39		0,597
3	20,393	79927,771	51,0	0,49		1,955
4	30,577	2675,029	1,7	0,72		0,460
	Total	156568,706	100,0			4,478

Figure S6. KR of *t*-Butyl 3-hydroxy-3-(4-metoxyphenyl)propanoate, **2f**.

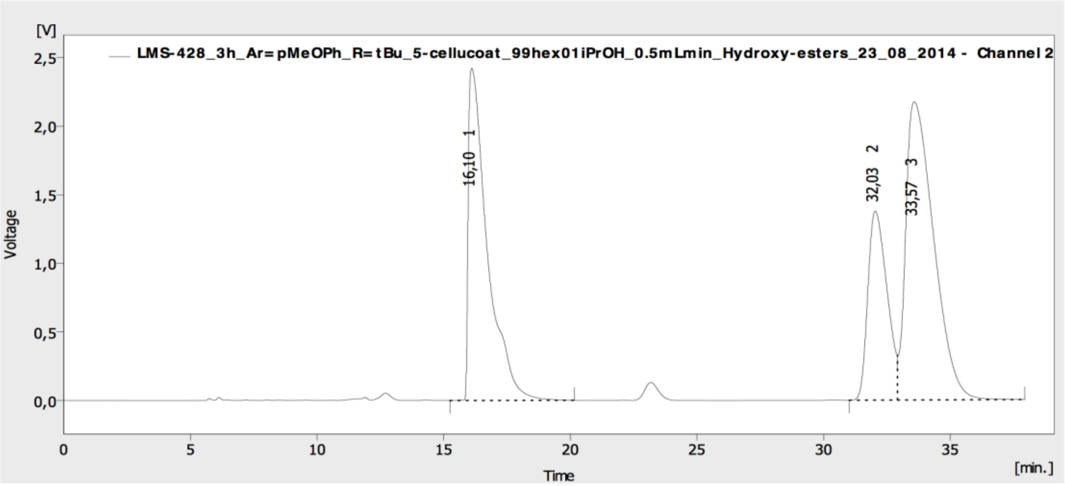

Result Table (Uncal - LMS-428_3h_Ar=pMeOPh_R=tBu_5-cellucoat_99hex01iPrOH_0.5mLmin_Hydroxy-esters_23_08_2014 - Channel 2)

	Reten. Time [min]	Area [mV.s]	Area [%]	W05 [min]	Compound Name	ee
1	16,100	126818,774	34,6	0,72		5,248
2	32,033	72722,564	19,8	0,87		2,632
3	33,567	167291,121	45,6	1,19		4,494
	Total	366832,459	100,0			12,374

Separation of the Alcohol

Figure S6. *Cont.*

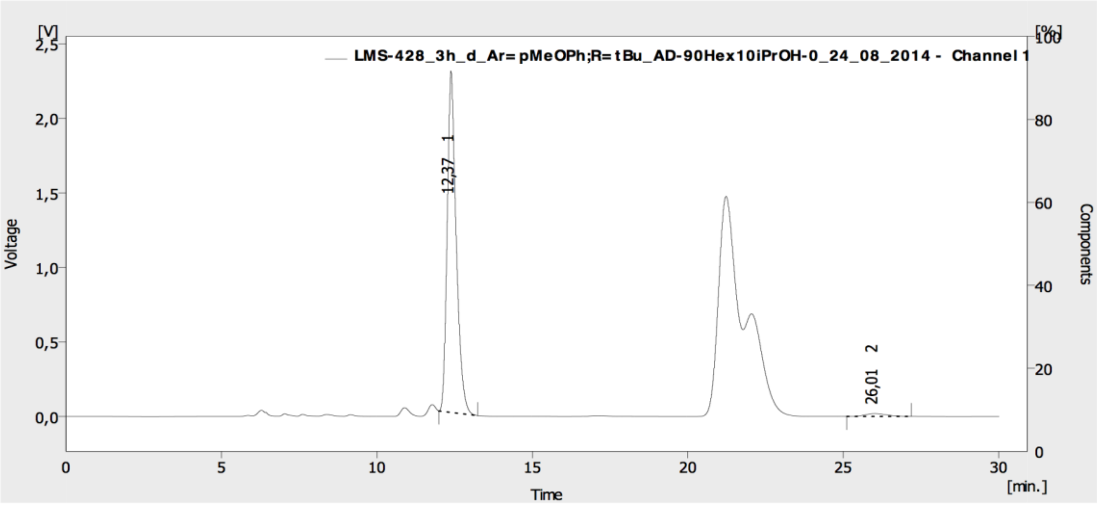

Result Table (Uncal - LMS-428_3h_d_Ar=pMeOPh;R=tBu_AD-90Hex10iPrOH-0_24_08_2014 - Channel 1)

	Reten. Time [min]	Area [mV.s]	Height [mV]	Area [%]	Height [%]	W05 [min]	Compound Name
1	12,373	46063,048	2291,214	98,0	99,2	0,30	
2	26,007	921,585	19,020	2,0	0,8	0,77	
	Total	46984,633	2310,233	100,0	100,0		

Separation of the Acetate

Figure S7. KR of *t*-Butyl 3-hydroxy-3-(2-naphthyl)propanoate, **2g**.

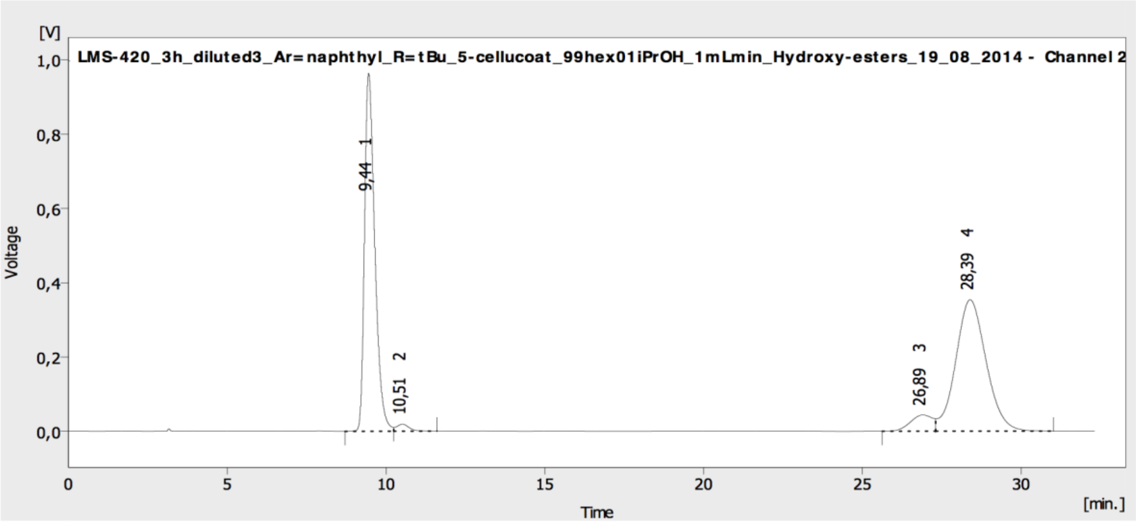

Result Table (Uncal - LMS-420_3h_diluted3_Ar=naphthyl_R=tBu_5-cellucoat_99hex01iPrOH_1mLmin_Hydroxy-esters _19_08_2014 - Channel 2)

	Reten. Time [min]	Area [mV.s]	Area [%]	W05 [min]	Compound Name
1	9,440	21753,138	45,3	0,35	
2	10,507	529,225	1,1	0,50	
3	26,890	2143,872	4,5	0,84	
4	28,390	23552,018	49,1	1,02	
	Total	47978,253	100,0		

Figure S8. KR of *t*-Butyl 3-hydroxy-3-(4-chloro)propanoate, **2h**.

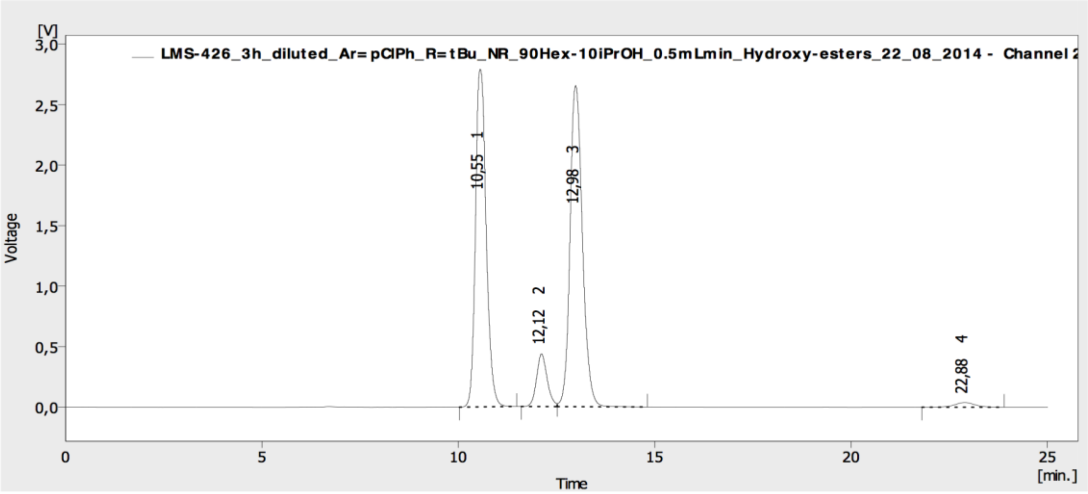

Result Table (Uncal - LMS-426_3h_diluted_Ar=pClPh_R=tBu_NR_90Hex-10iPrOH_0.5mLmin_Hydroxy-esters_22_08_2014 - Channel 2)

	Reten. Time [min]	Area [mV.s]	Area [%]	W05 [min]	Compound Name	ee
1	10,550	52764,331	43,7	0,29		1,833
2	12,117	8207,470	6,8	0,29		0,952
3	12,983	58346,341	48,3	0,34		1,462
4	22,883	1405,686	1,2	0,54		0,135
	Total	120723,828	100,0			4,383

Figure S9. KR of *t*-Butyl 3-hydroxy-3-(2,6-dichlorophenyl) propanoate, **2i**.

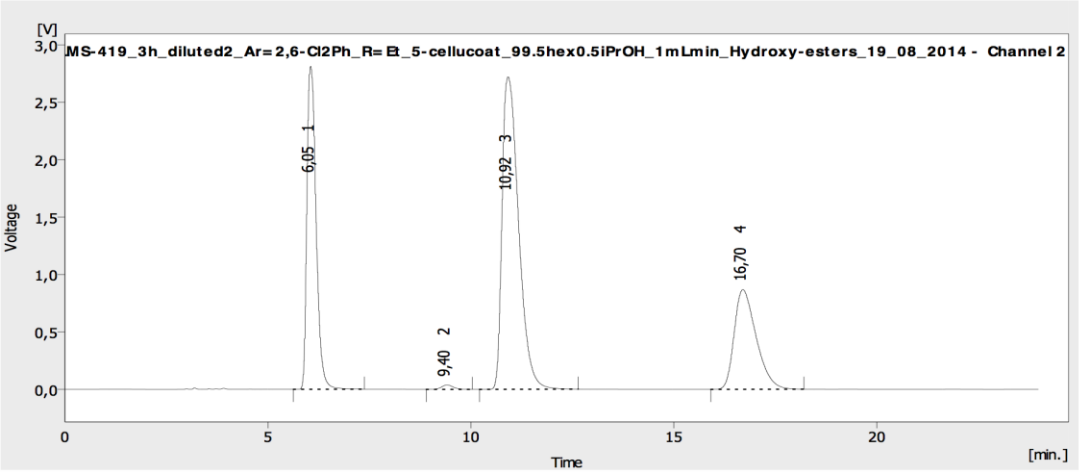

Result Table (Uncal - LMS-419_3h_diluted2_Ar=2,6-Cl2Ph_R=Et_5-cellucoat_99.5hex0.5iPrOH_1mLmin_Hydroxy-esters_19_08_2014 - Channel 2)

	Reten. Time [min]	Area [mV.s]	Area [%]	W05 [min]	Compound Name
1	6,050	44365,759	28,7	0,25	
2	9,400	769,839	0,5	0,32	
3	10,917	76856,431	49,7	0,44	
4	16,700	32603,014	21,1	0,58	
	Total	154595,044	100,0		

Supplementary Section 2. HPLC Chromatograms of Isolated Compounds

The HPLC chromatograms of the racemic **2a**–**i** and **3a–i** and their corresponding enantiopure counterparts obtained after the kinetic resolution are attached below. The HPLC chromatograms of *rac*-**4** and (*S*)-**4** are also included.

Figure S10. Ethyl 3-hydroxy-3-phenylpropanoate, **2a**.

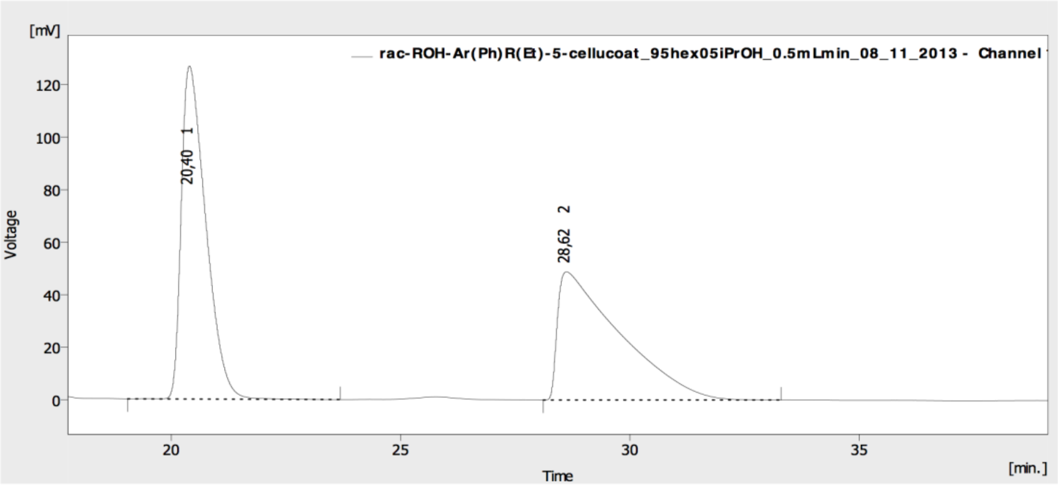

Result Table (Uncal - rac-ROH-Ar(Ph)R(Et)-5-cellucoat_95hex05iPrOH_0.5mLmin_08_11_2013 - Channel 1)

	Reten. Time [min]	Area [mV.s]	Area [%]	W05 [min]	Compound Name
1	20,400	4708,591	50,6	0,58	
2	28,617	4588,556	49,4	1,44	
	Total	9297,147	100,0		

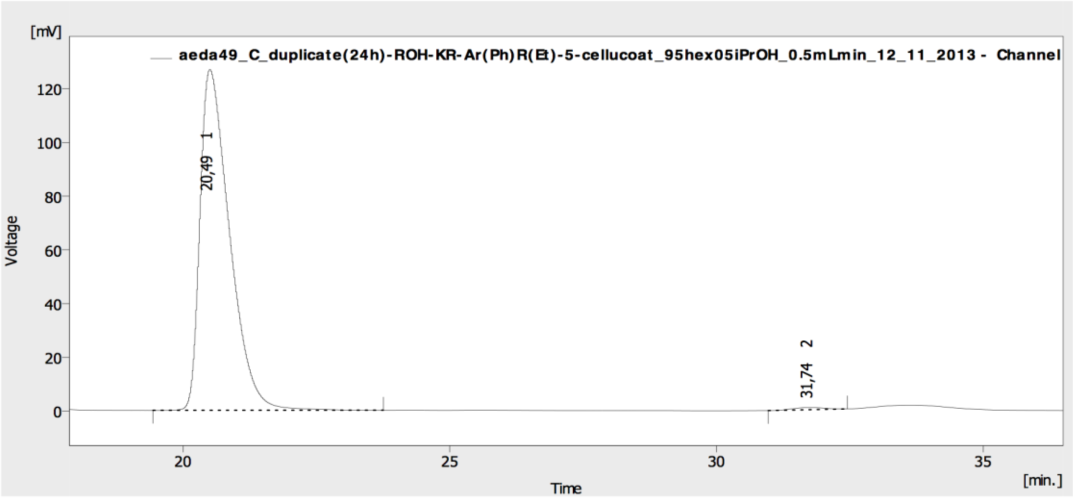

Result Table (Uncal - aeda49_C_duplicate(24h)-ROH-KR-Ar(Ph)R(Et)-5-cellucoat_95hex05iPrOH_0.5mLmin_12_11_2013 - Channel 1)

	Reten. Time [min]	Area [mV.s]	Area [%]	W05 [min]	Compound Name
1	20,490	4880,501	99,3	0,59	
2	31,740	33,856	0,7	0,70	
	Total	4914,356	100,0		

Figure S11. Ethyl 3-hydroxy-3-(4-nitrophenyl)propanoate, **2b**.

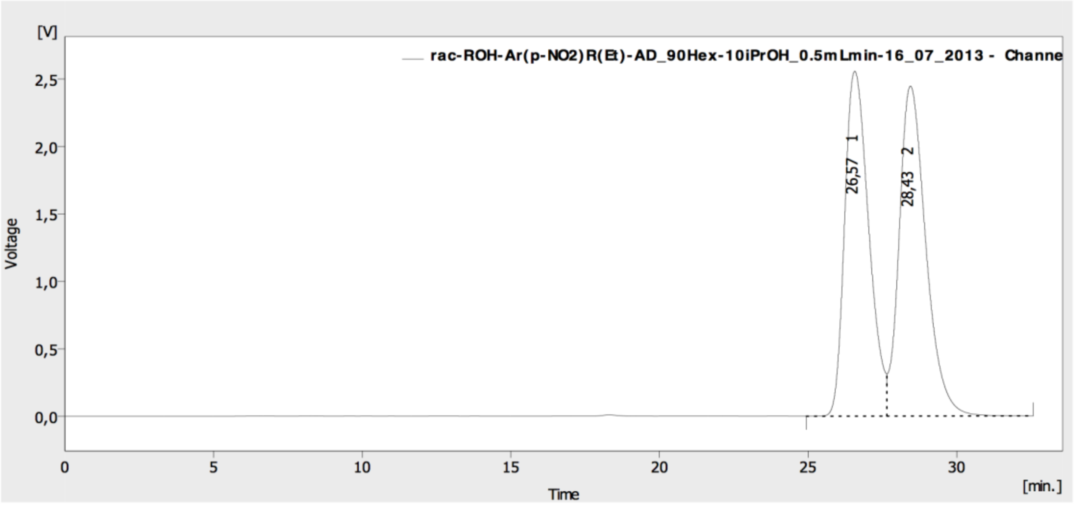

Result Table (Uncal - rac-ROH-Ar(p-NO2)R(Et)-AD_90Hex-10iPrOH_0.5mLmin-16_07_2013 - Channel 1)

	Reten. Time [min]	Area [mV.s]	Area [%]	W05 [min]	Compound Name
1	26,567	144218,712	48,5	0,89	
2	28,433	153158,124	51,5	0,95	
	Total	297376,836	100,0		

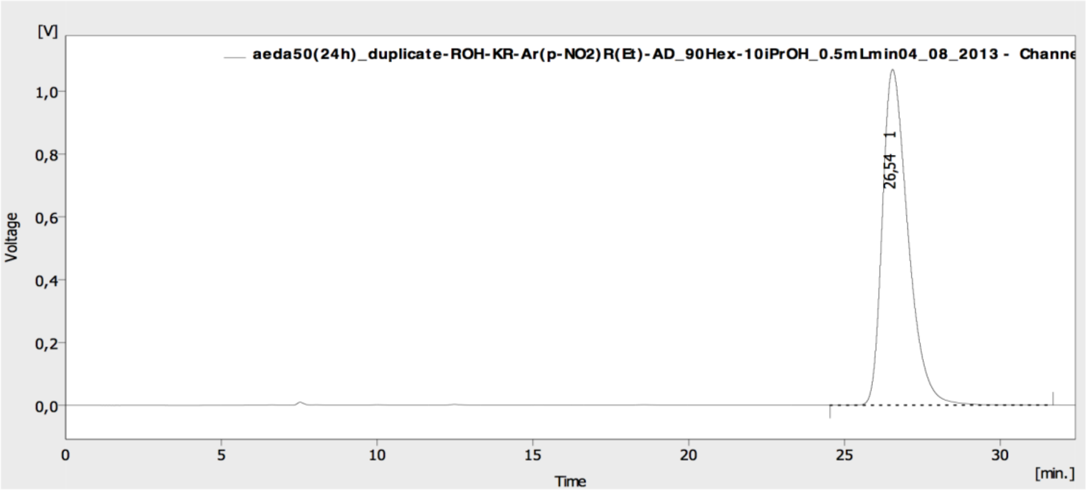

Result Table (Uncal - aeda50(24h)_duplicate-ROH-KR-Ar(p-NO2)R(Et)-AD_90Hex-10iPrOH_0.5mLmin04_08_2013 - Channel 1)

	Reten. Time [min]	Area [mV.s]	Area [%]	W05 [min]	Compound Name
1	26,540	61279,782	100,0	0,87	
	Total	61279,782	100,0		

Figure S12. Ethyl 3-hydroxy-3-(4-metoxyphenyl)propanoate, **2c**.

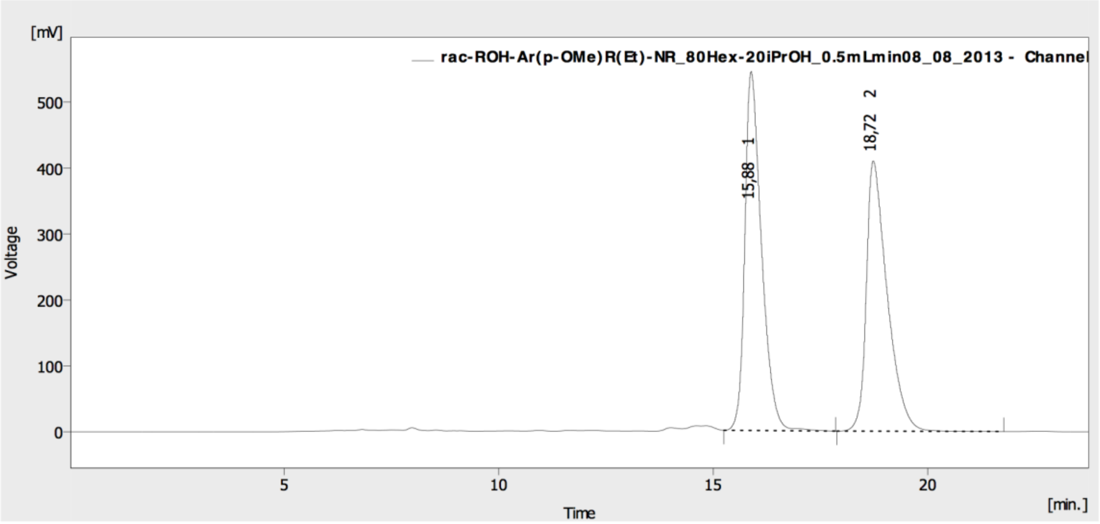

Result Table (Uncal - rac-ROH-Ar(p-OMe)R(Et)-NR_80Hex-20iPrOH_0.5mLmin08_08_2013 - Channel 1)

	Reten. Time [min]	Area [mV.s]	Area [%]	W05 [min]	Compound Name
1	15,883	14721,678	51,5	0,41	
2	18,717	13856,642	48,5	0,50	
	Total	28578,320	100,0		

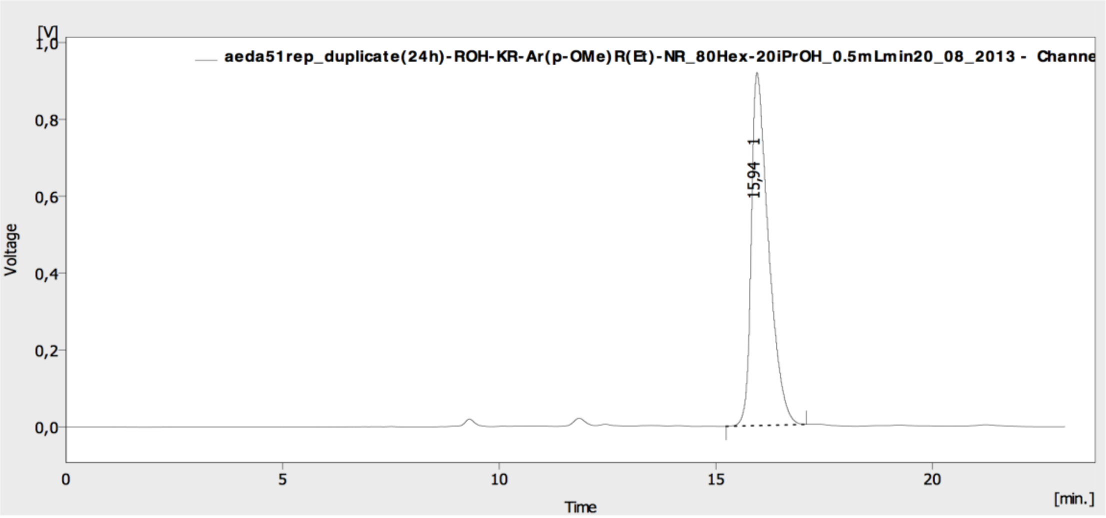

Result Table (Uncal - aeda51rep_duplicate(24h)-ROH-KR-Ar(p-OMe)R(Et)-NR_80Hex-20iPrOH_0.5mLmin20_08_2013 - Channel 1)

	Reten. Time [min]	Area [mV.s]	Area [%]	W05 [min]	Compound Name
1	15,940	25470,285	100,0	0,42	
	Total	25470,285	100,0		

Figure S13. *t*-Butyl 3-hydroxy-3-phenylpropanoate, **2d**.

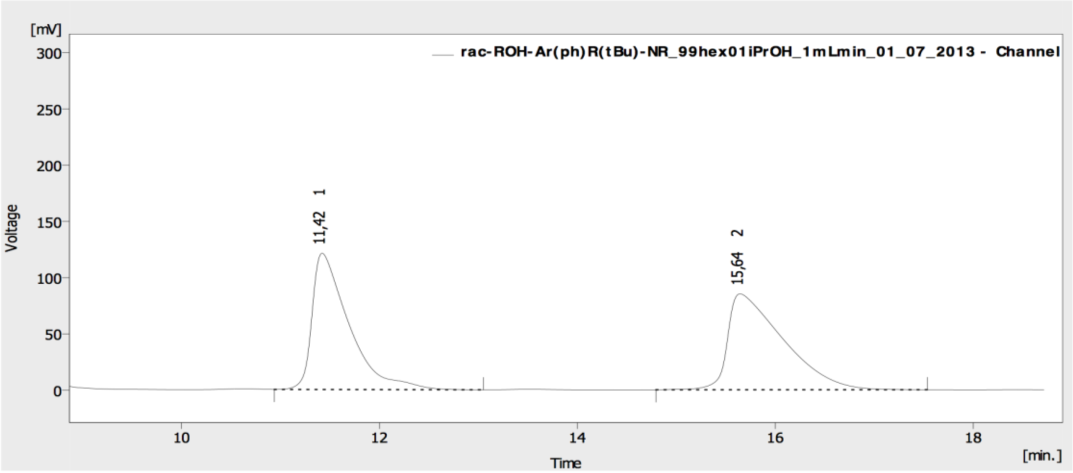

Result Table (Uncal - rac-ROH-Ar(ph)R(tBu)-NR_99hex01iPrOH_1mLmin_01_07_2013 - Channel 1)

	Reten. Time [min]	Area [mV.s]	Area [%]	W05 [min]	Compound Name
1	11,420	3140,841	48,6	0,37	
2	15,643	3316,039	51,4	0,59	
	Total	6456,881	100,0		

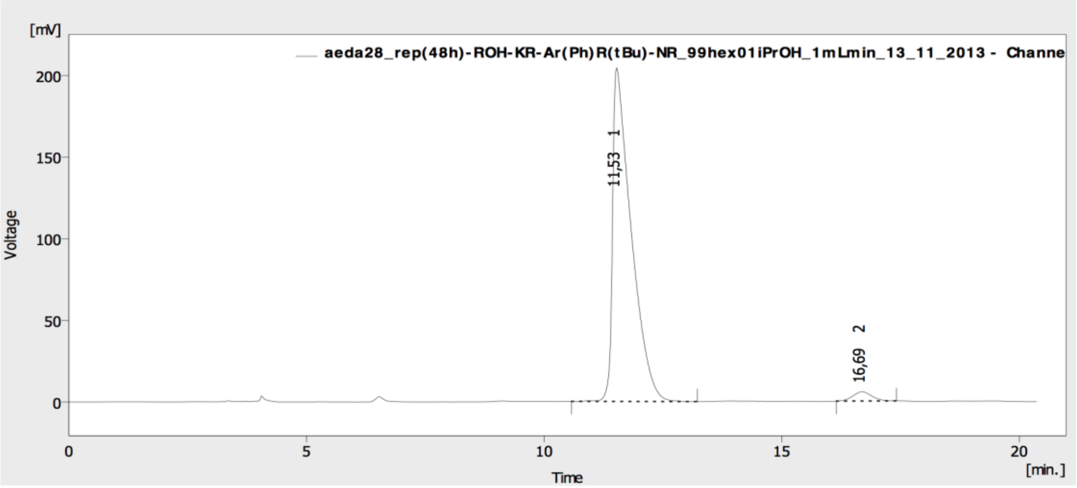

Result Table (Uncal - aeda28_rep(48h)-ROH-KR-Ar(Ph)R(tBu)-NR_99hex01iPrOH_1mLmin_13_11_2013 - Channel 1)

	Reten. Time [min]	Area [mV.s]	Area [%]	W05 [min]	Compound Name
1	11,527	5567,420	97,3	0,41	
2	16,687	156,661	2,7	0,43	
	Total	5724,081	100,0		

Figure S14. *t*-Butyl 3-hydroxy-3-(4-nitrophenyl)propanoate, **2e**.

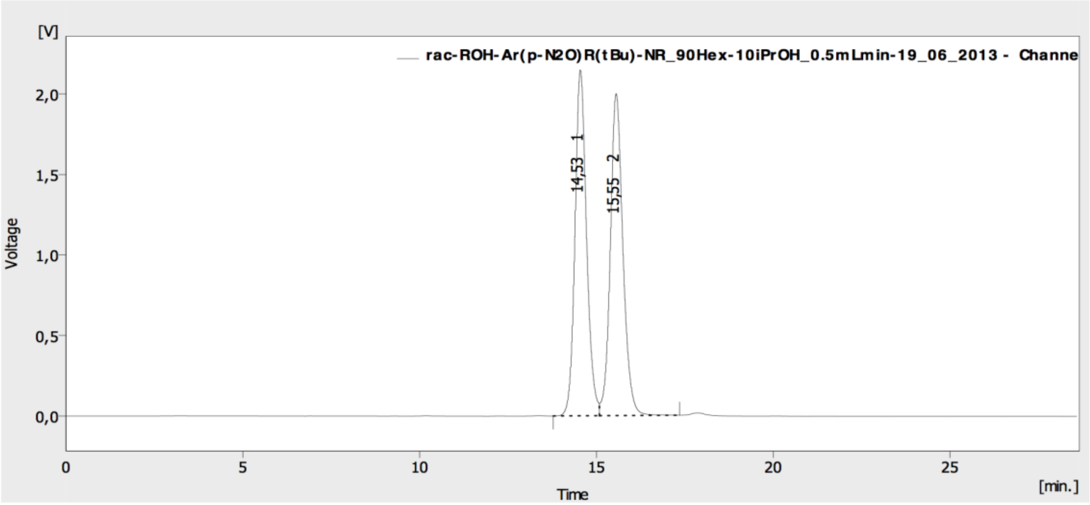

Result Table (Uncal - rac-ROH-Ar(p-N2O)R(tBu)-NR_90Hex-10iPrOH_0.5mLmin-19_06_2013 - Channel 1)

	Reten. Time [min]	Area [mV.s]	Area [%]	W05 [min]	Compound Name
1	14,533	48876,849	49,5	0,35	
2	15,550	49828,603	50,5	0,37	
	Total	98705,452	100,0		

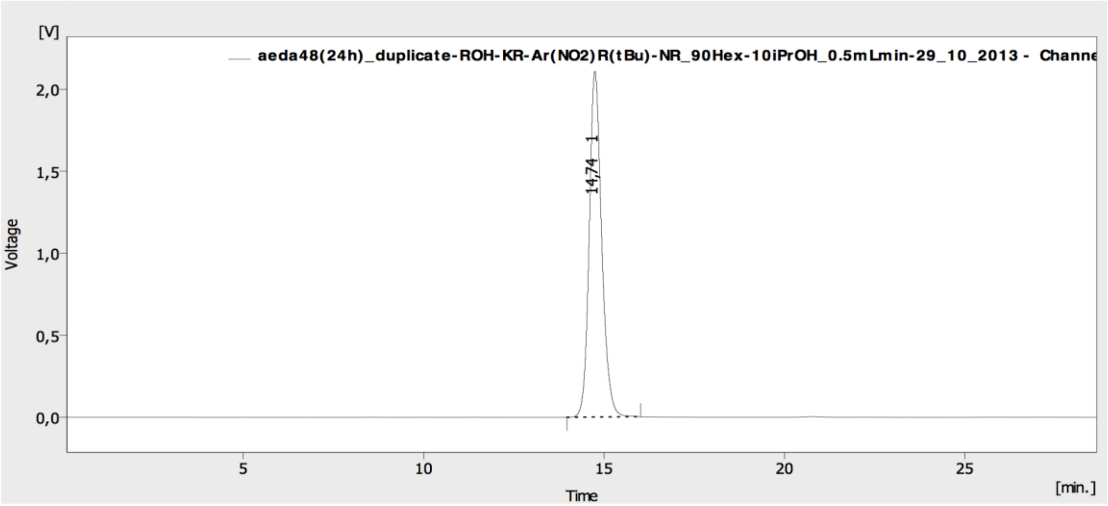

Result Table (Uncal - aeda48(24h)_duplicate-ROH-KR-Ar(NO2)R(tBu)-NR_90Hex-10iPrOH_0.5mLmin-29_10_2013 - Channel 1)

	Reten. Time [min]	Area [mV.s]	Area [%]	W05 [min]	Compound Name
1	14,740	50383,495	100,0	0,35	
	Total	50383,495	100,0		

Figure S15. *t*-Butyl 3-hydroxy-3-(4-metoxyphenyl)propanoate, **2f**. The chromatograms attached below correspond to the racemic acetate and the acetylated alcohol.

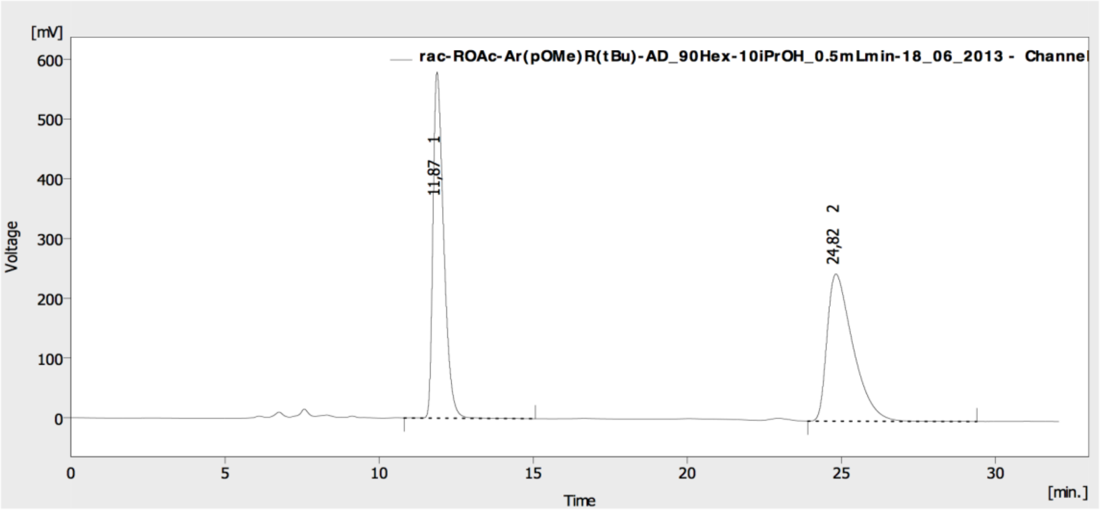

Result Table (Uncal - rac-ROAc-Ar(pOMe)R(tBu)-AD_90Hex-10iPrOH_0.5mLmin-18_06_2013 - Channel 1)

	Reten. Time [min]	Area [mV.s]	Area [%]	W05 [min]	Compound Name
1	11,873	14012,667	49,3	0,37	
2	24,823	14401,498	50,7	0,89	
	Total	28414,165	100,0		

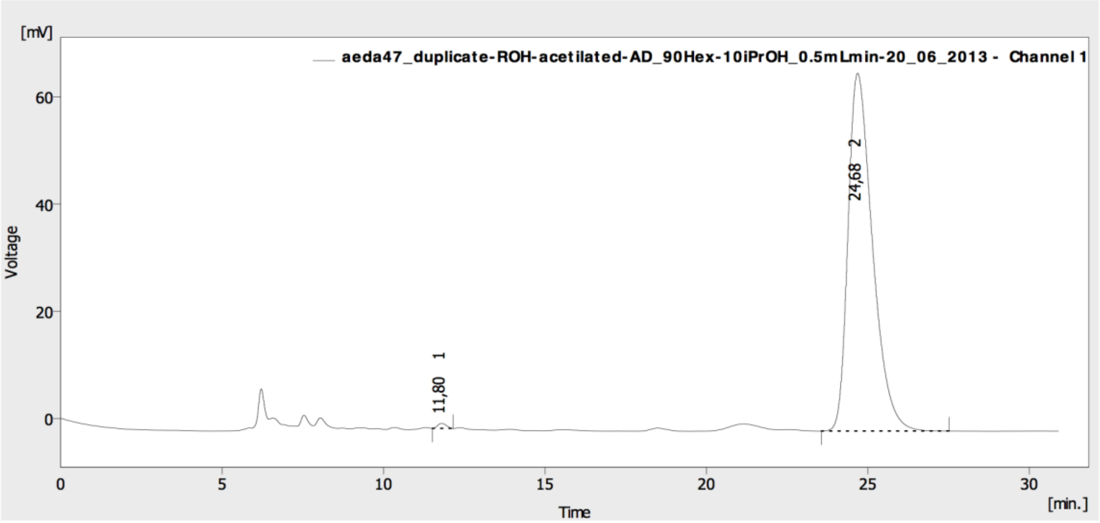

Result Table (Uncal - aeda47_duplicate-ROH-acetilated-AD_90Hex-10iPrOH_0.5mLmin-20_06_2013 - Channel 1)

	Reten. Time [min]	Area [mV.s]	Area [%]	W05 [min]	Compound Name
1	11,800	17,645	0,5	0,31	
2	24,683	3514,087	99,5	0,80	
	Total	3531,731	100,0		

Figure S16. *t*-Butyl 3-hydroxy-3-(2-naphthyl)propanoate, **2g**.

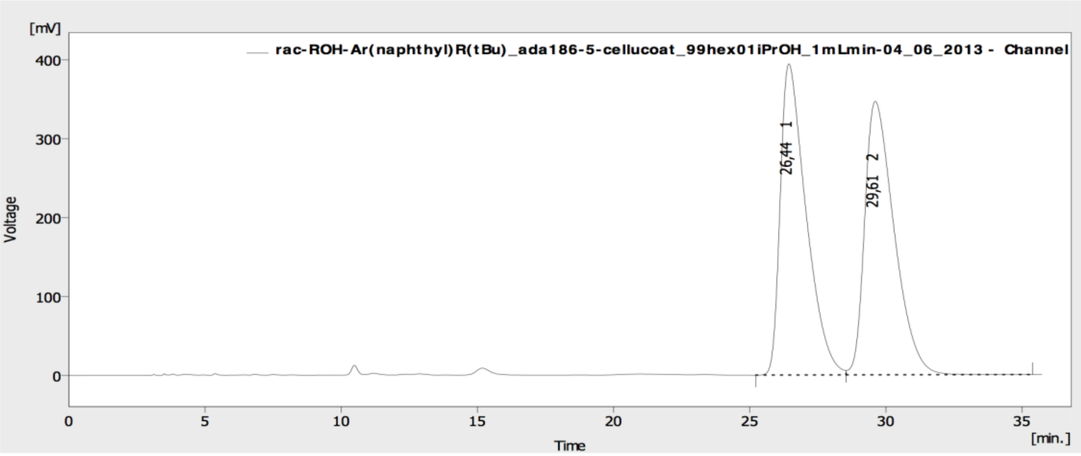

Result Table (Uncal - rac-ROH-Ar(naphthyl)R(tBu)_ada186-5-cellucoat_99hex01iPrOH_1mLmin-04_06_2013 - Channel 1)

	Reten. Time [min]	Area [mV.s]	Area [%]	W05 [min]	Compound Name
1	26,440	26035,941	49,9	1,01	
2	29,613	26117,077	50,1	1,16	
	Total	52153,019	100,0		

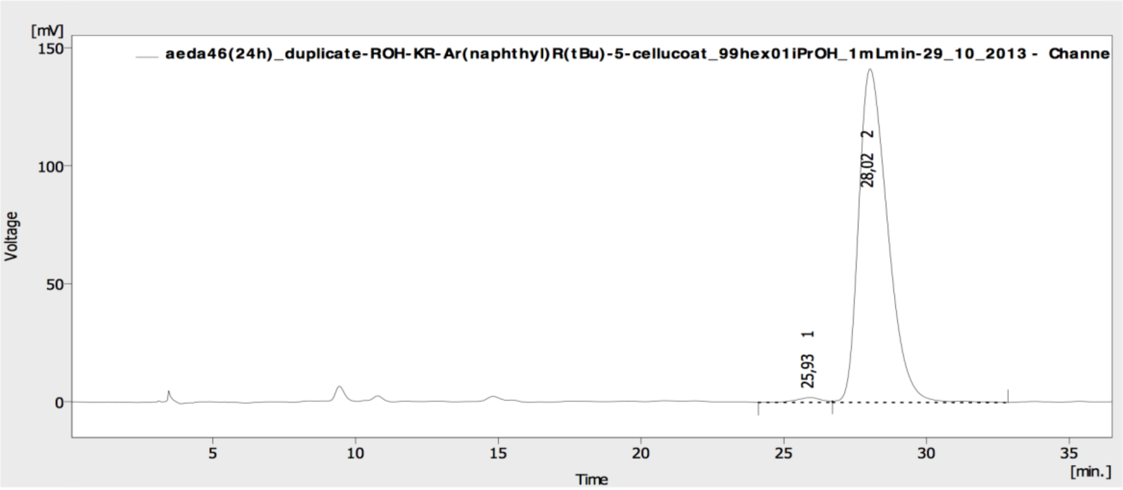

Result Table (Uncal - aeda46(24h)_duplicate-ROH-KR-Ar(naphthyl)R(tBu)-5-cellucoat_99hex01iPrOH_1mLmin-29_10_2013 - Channel 1)

	Reten. Time [min]	Area [mV.s]	Area [%]	W05 [min]	Compound Name
1	25,933	126,073	1,2	0,93	
2	28,023	9976,615	98,8	1,09	
	Total	10102,688	100,0		

Figure S17. *t*-Butyl 3-hydroxy-3-(4-chloro)propanoate, **2h**.

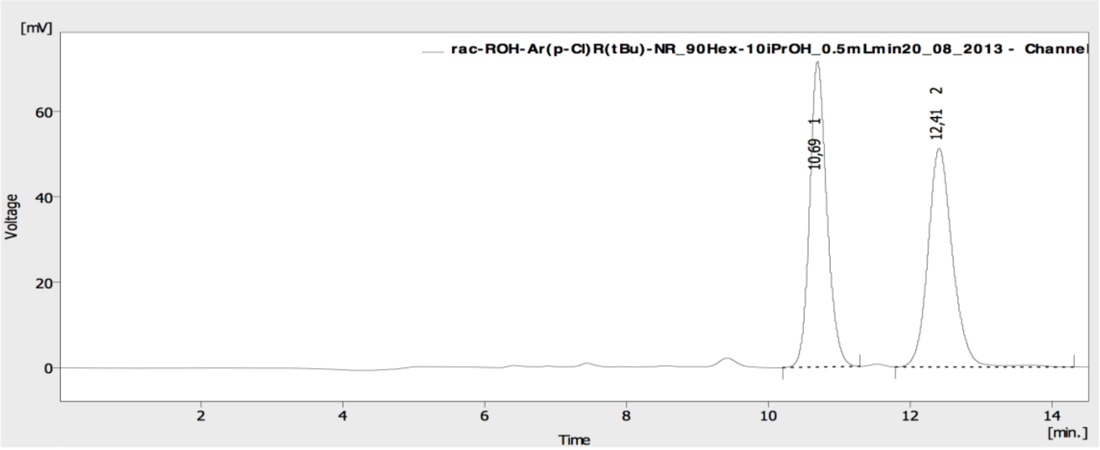

Result Table (Uncal - rac-ROH-Ar(p-Cl)R(tBu)-NR_90Hex-10iPrOH_0.5mLmin20_08_2013 - Channel 1)

	Reten. Time [min]	Area [mV.s]	Area [%]	W05 [min]	Compound Name
1	10,690	1205,182	49,4	0,25	
2	12,407	1235,165	50,6	0,37	
	Total	2440,347	100,0		

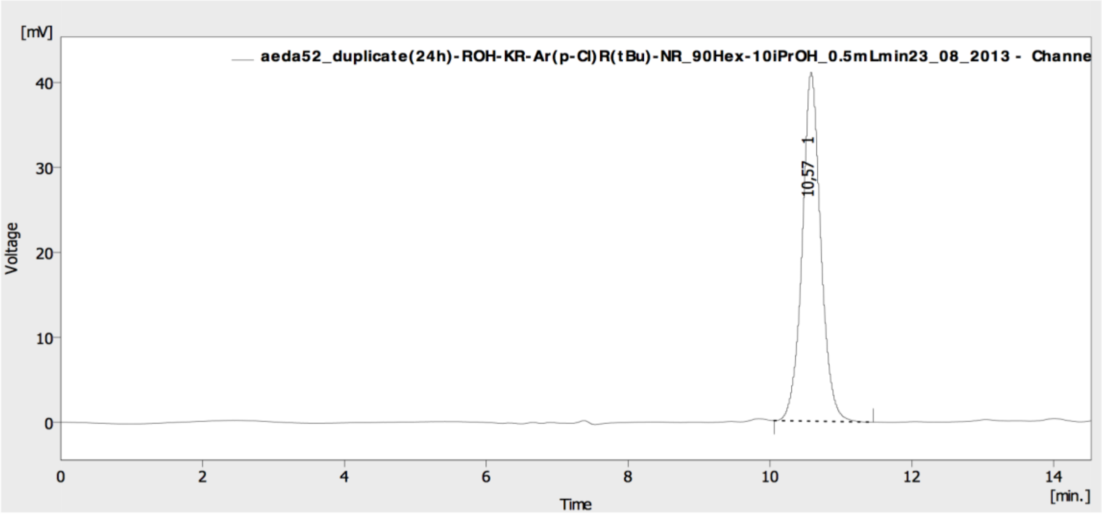

Result Table (Uncal - aeda52_duplicate(24h)-ROH-KR-Ar(p-Cl)R(tBu)-NR_90Hex-10iPrOH_0.5mLmin23_08_2013 - Channel 1)

	Reten. Time [min]	Area [mV.s]	Area [%]	W05 [min]	Compound Name
1	10,573	747,554	100,0	0,27	
	Total	747,554	100,0		

Figure S18. *t*-Butyl 3-hydroxy-3-(2,6-dichlorophenyl) propanoate, **2i**.

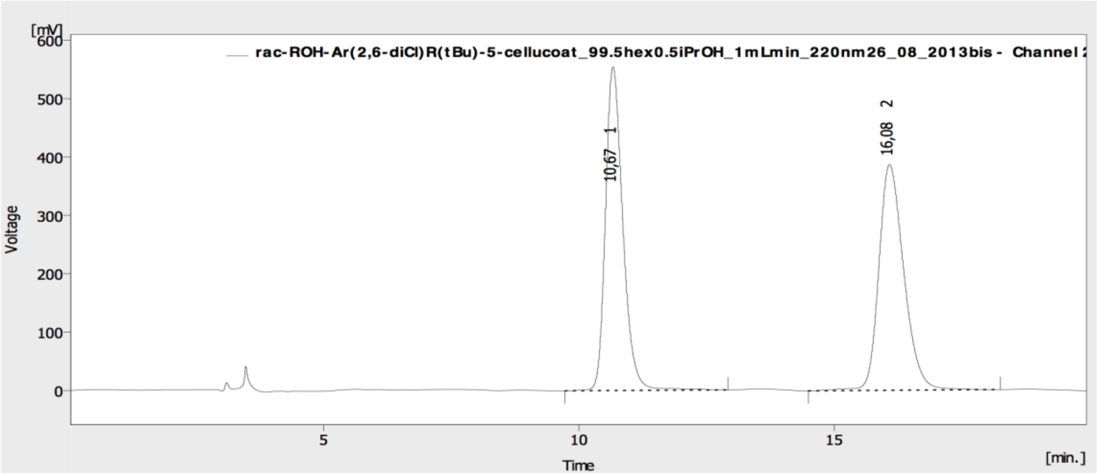

Result Table (Uncal - rac-ROH-Ar(2,6-diCl)R(tBu)-5-cellucoat_99.5hex0.5iPrOH_1mLmin_220nm26_08_2013bis - Channel 2)

	Reten. Time [min]	Area [mV.s]	Area [%]	W05 [min]	Compound Name
1	10,667	13156,869	49,9	0,35	
2	16,083	13192,207	50,1	0,52	
	Total	26349,076	100,0		

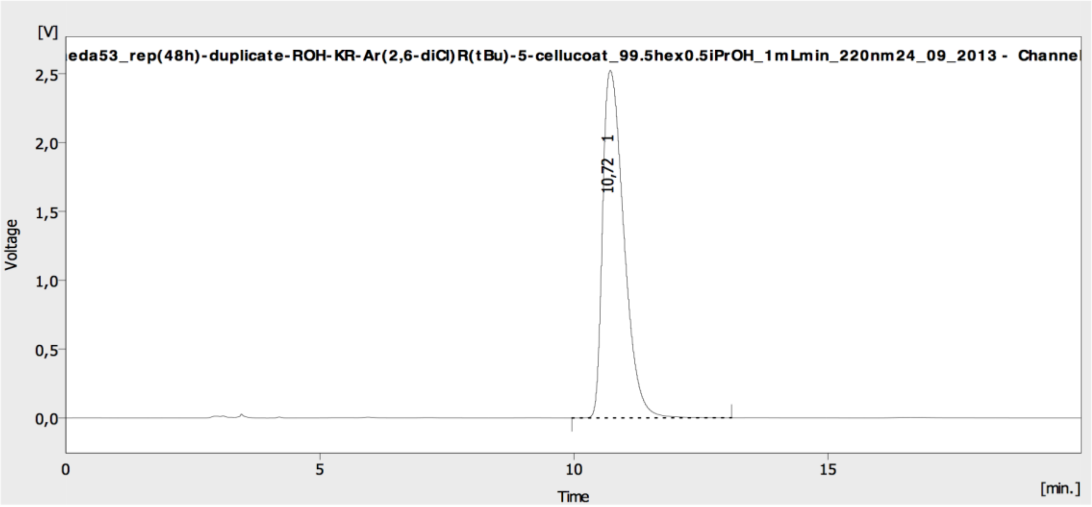

Result Table (Uncal - aeda53_rep(48h)-duplicate-ROH-KR-Ar(2,6-diCl)R(tBu)-5-cellucoat_99.5hex0.5iPrOH_1mLmin_220nm24_09_2013 - Channel 2)

	Reten. Time [min]	Area [mV.s]	Area [%]	W05 [min]	Compound Name
1	10,717	73324,000	100,0	0,45	
	Total	73324,000	100,0		

Figure S19. Ethyl 3-acetoxy-3-phenylpropanoate, **3a**.

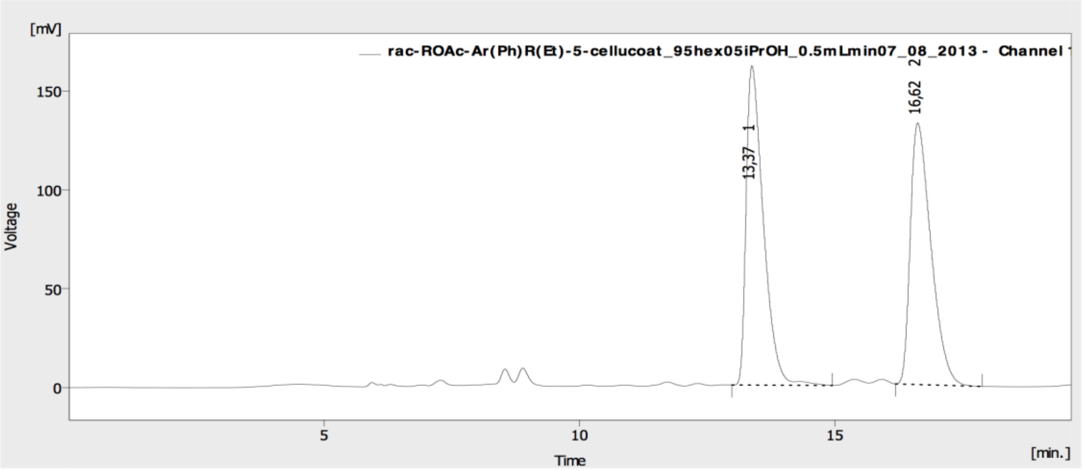

Result Table (Uncal - rac-ROAc-Ar(Ph)R(Et)-5-cellucoat_95hex05iPrOH_0.5mLmin07_08_2013 - Channel 1)

	Reten. Time [min]	Area [mV.s]	Area [%]	W05 [min]	Compound Name
1	13,367	3800,711	50,5	0,37	
2	16,617	3726,980	49,5	0,44	
	Total	7527,691	100,0		

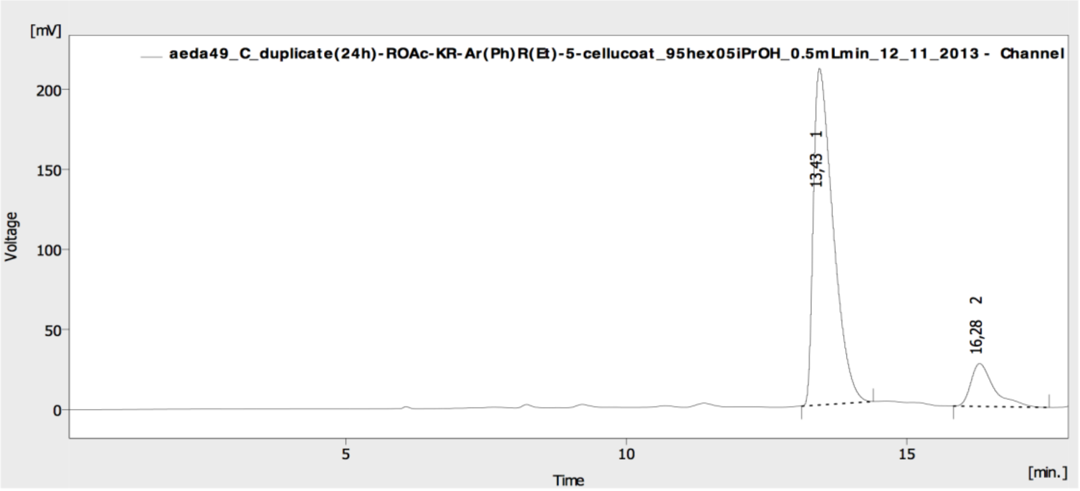

Result Table (Uncal - aeda49_C_duplicate(24h)-ROAc-KR-Ar(Ph)R(Et)-5-cellucoat_95hex05iPrOH_0.5mLmin_12_11_2013 - Channel 1)

	Reten. Time [min]	Area [mV.s]	Area [%]	W05 [min]	Compound Name
1	13,433	5159,609	87,0	0,39	
2	16,283	772,741	13,0	0,39	
	Total	5932,350	100,0		

Figure S20. Ethyl 3-acetoxy-3-(4-nitrophenyl)propanoate, **3b**.

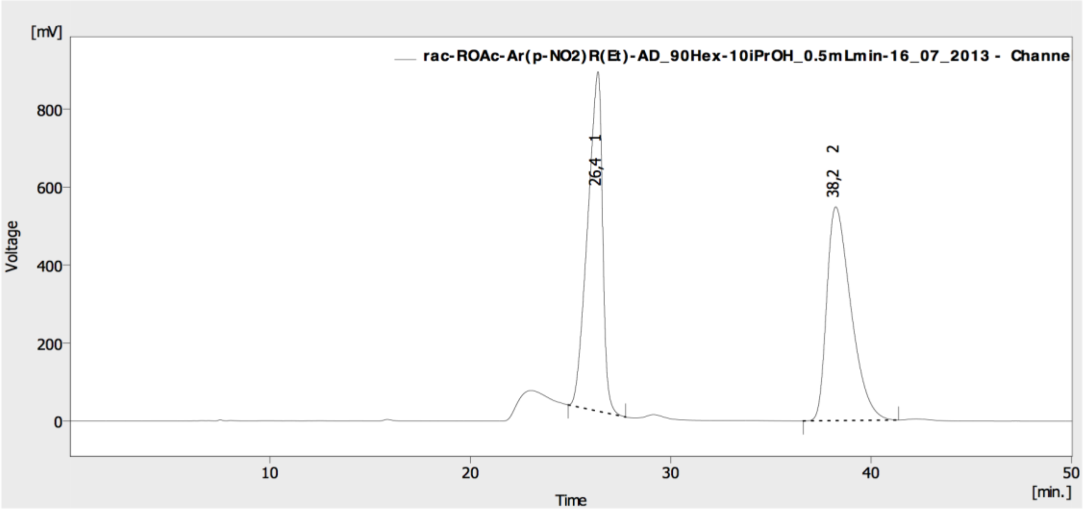

Result Table (Uncal - rac-ROAc-Ar(p-NO2)R(Et)-AD_90Hex-10iPrOH_0.5mLmin-16_07_2013 - Channel 1)

	Reten. Time [min]	Area [mV.s]	Area [%]	W05 [min]	Compound Name
1	26,367	45053,331	49,4	0,82	
2	38,233	46121,967	50,6	1,29	
	Total	91175,298	100,0		

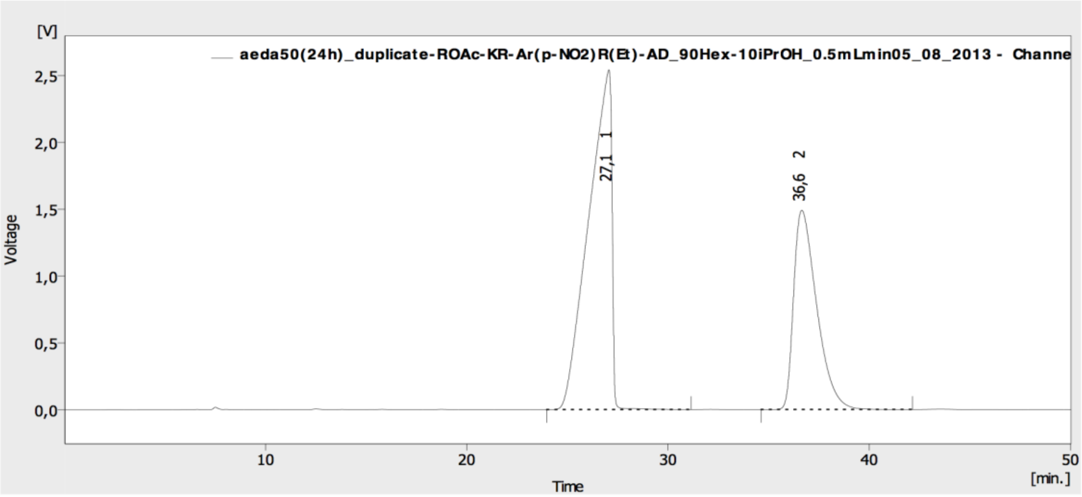

Result Table (Uncal - aeda50(24h)_duplicate-ROAc-KR-Ar(p-NO2)R(Et)-AD_90Hex-10iPrOH_0.5mLmin05_08_2013 - Channel 1)

	Reten. Time [min]	Area [mV.s]	Area [%]	W05 [min]	Compound Name
1	27,060	202385,385	63,0	1,31	
2	36,643	118608,531	37,0	1,23	
	Total	320993,917	100,0		

Figure S21. Ethyl 3-acetoxy-3-(4-metoxyphenyl)propanoate, **3c**.

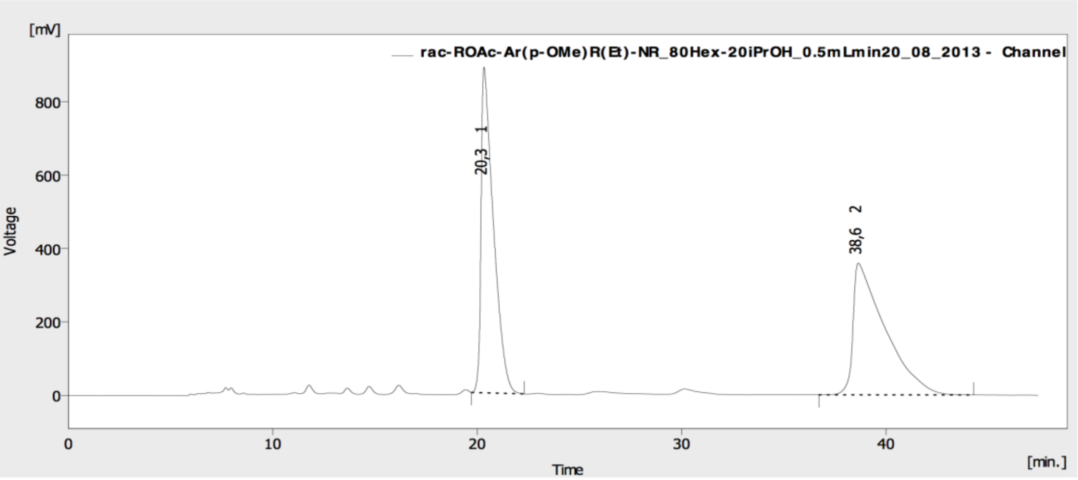

Result Table (Uncal - rac-ROAc-Ar(p-OMe)R(Et)-NR_80Hex-20iPrOH_0.5mLmin20_08_2013 - Channel 1)

	Reten. Time [min]	Area [mV.s]	Area [%]	W05 [min]	Compound Name
1	20,323	37197,261	48,8	0,64	
2	38,640	39102,838	51,2	1,60	
	Total	76300,099	100,0		

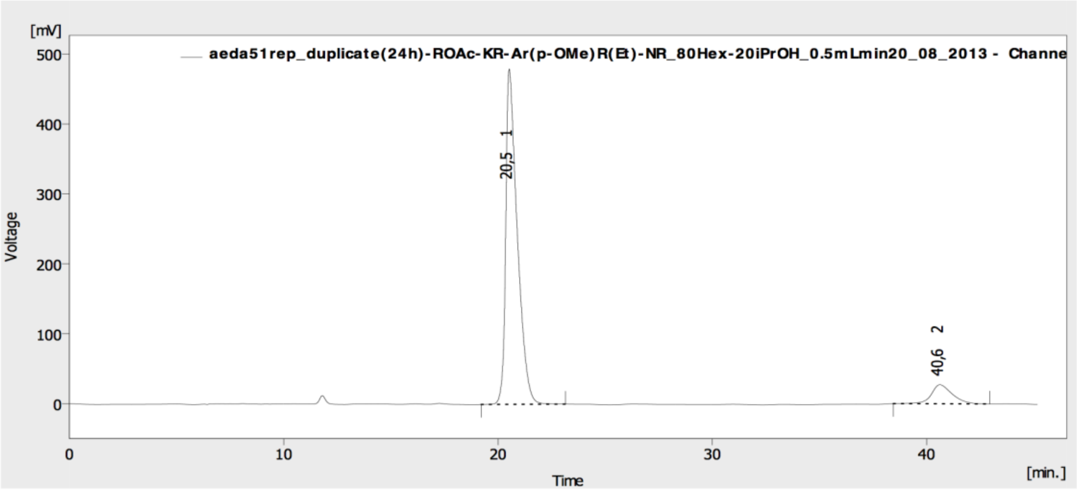

Result Table (Uncal - aeda51rep_duplicate(24h)-ROAc-KR-Ar(p-OMe)R(Et)-NR_80Hex-20iPrOH_0.5mLmin20_08_2013 - Channel 1)

	Reten. Time [min]	Area [mV.s]	Area [%]	W05 [min]	Compound Name
1	20,517	17862,221	90,5	0,55	
2	40,617	1865,094	9,5	1,00	
	Total	19727,315	100,0		

Figure S22. *t*-Butyl 3-acetoxy-3-phenylpropanoate, **3d**.

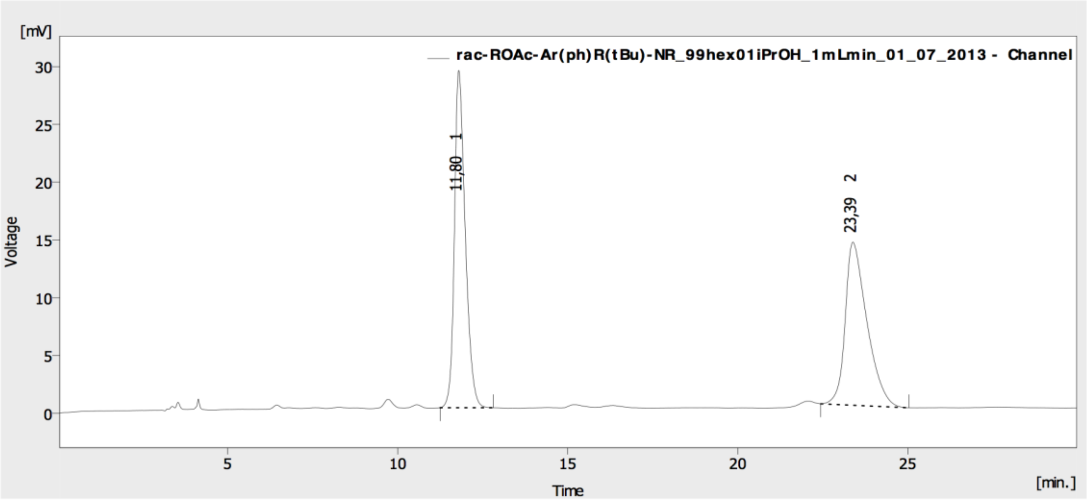

Result Table (Uncal - rac-ROAc-Ar(ph)R(tBu)-NR_99hex01iPrOH_1mLmin_01_07_2013 - Channel 1)

	Reten. Time [min]	Area [mV.s]	Area [%]	W05 [min]	Compound Name
1	11,797	645,415	50,8	0,33	
2	23,387	625,742	49,2	0,66	
	Total	1271,157	100,0		

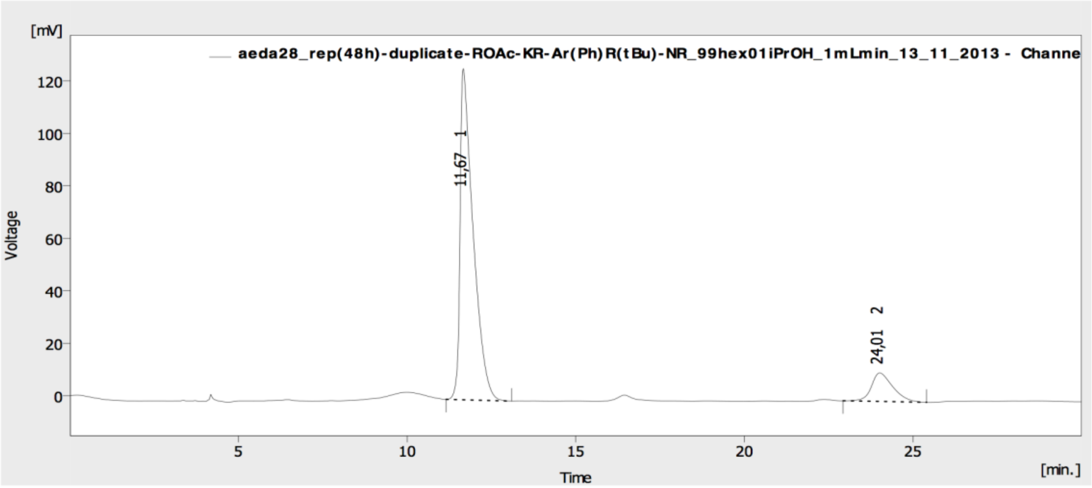

Result Table (Uncal - aeda28_rep(48h)-duplicate-ROAc-KR-Ar(Ph)R(tBu)-NR_99hex01iPrOH_1mLmin_13_11_2013 - Channel 1)

	Reten. Time [min]	Area [mV.s]	Area [%]	W05 [min]	Compound Name
1	11,667	3394,340	87,6	0,40	
2	24,013	480,017	12,4	0,66	
	Total	3874,357	100,0		

Figure S23. *t*-Butyl 3-acetoxy-3-(4-nitrophenyl)propanoate, **3e**.

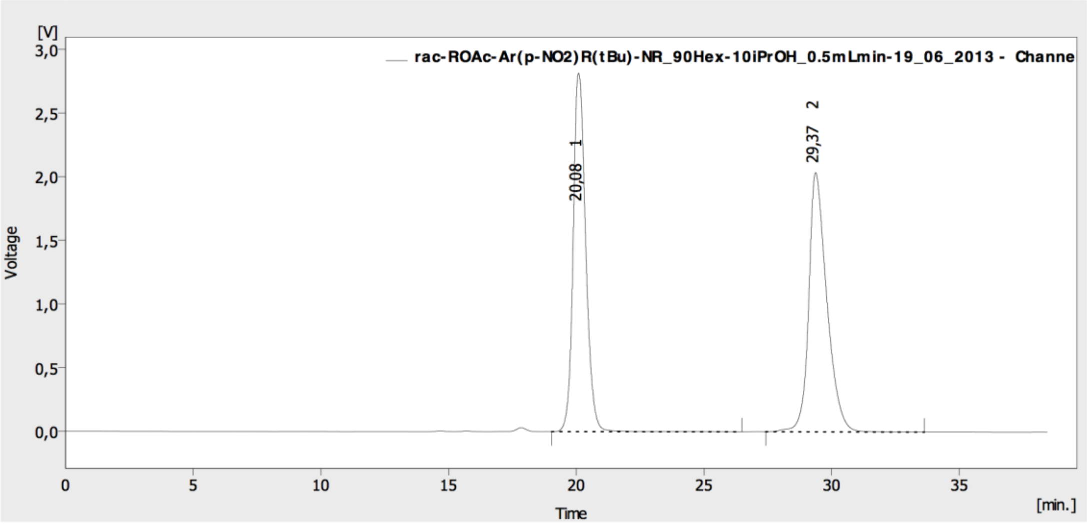

Result Table (Uncal - rac-ROAc-Ar(p-NO2)R(tBu)-NR_90Hex-10iPrOH_0.5mLmin-19_06_2013 - Channel 1)

	Reten. Time [min]	Area [mV.s]	Area [%]	W05 [min]	Compound Name
1	20,083	96513,561	48,9	0,52	
2	29,367	100670,653	51,1	0,72	
	Total	197184,214	100,0		

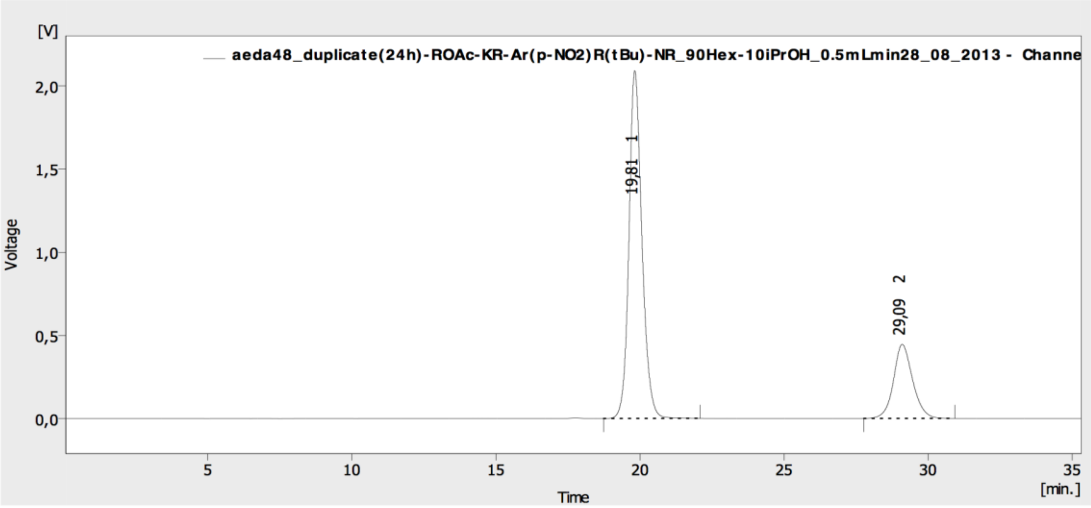

Result Table (Uncal - aeda48_duplicate(24h)-ROAc-KR-Ar(p-NO2)R(tBu)-NR_90Hex-10iPrOH_0.5mLmin28_08_2013 - Channel 1)

	Reten. Time [min]	Area [mV.s]	Area [%]	W05 [min]	Compound Name
1	19,807	65408,650	76,1	0,47	
2	29,090	20589,628	23,9	0,69	
	Total	85998,278	100,0		

Figure S24. *t*-Butyl 3-acetoxy-3-(4-metoxyphenyl)propanoate, **3f**.

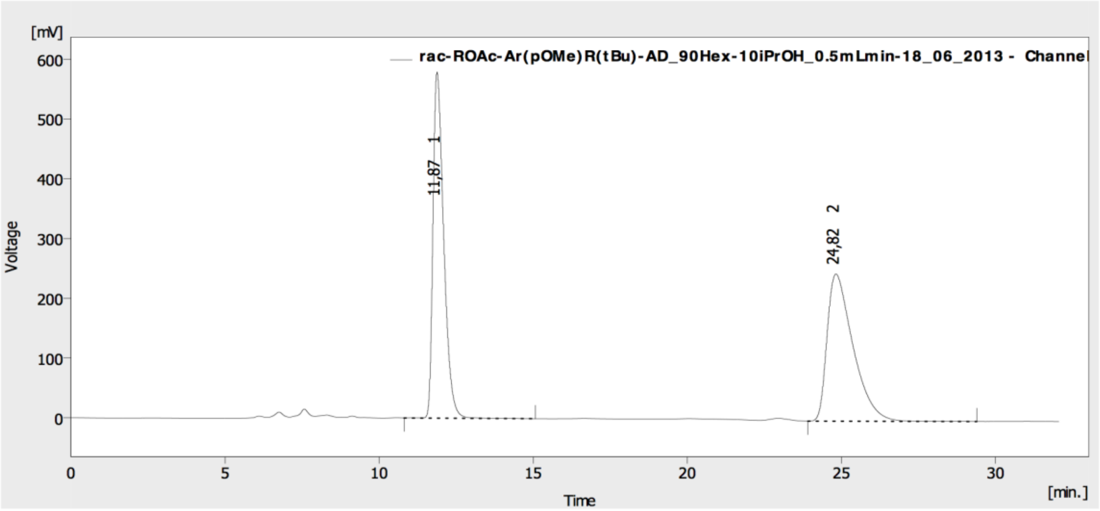

Result Table (Uncal - rac-ROAc-Ar(pOMe)R(tBu)-AD_90Hex-10iPrOH_0.5mLmin-18_06_2013 - Channel 1)

	Reten. Time [min]	Area [mV.s]	Area [%]	W05 [min]	Compound Name
1	11,873	14012,667	49,3	0,37	
2	24,823	14401,498	50,7	0,89	
	Total	28414,165	100,0		

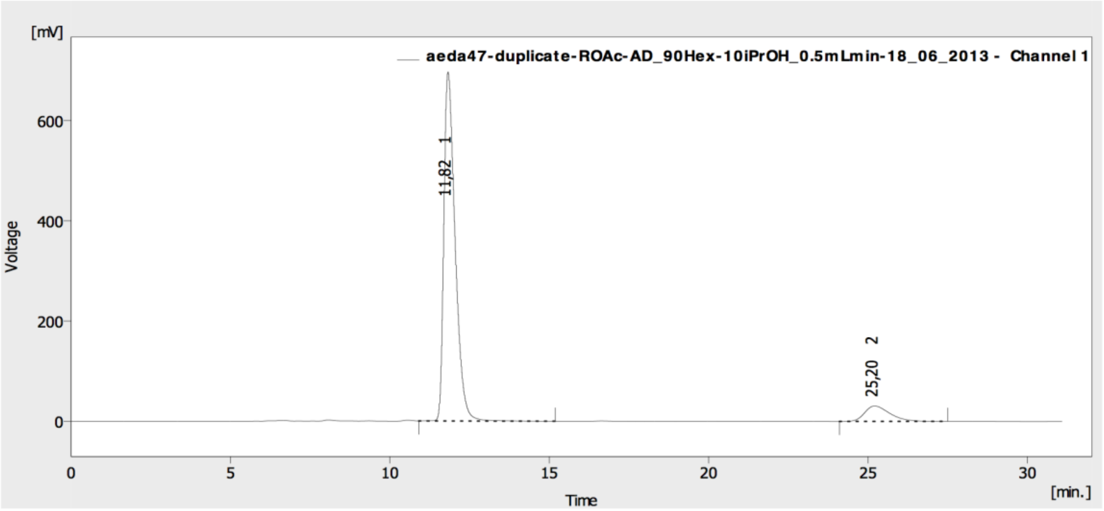

Result Table (Uncal - aeda47-duplicate-ROAc-AD_90Hex-10iPrOH_0.5mLmin-18_06_2013 - Channel 1)

	Reten. Time [min]	Area [mV.s]	Area [%]	W05 [min]	Compound Name
1	11,817	17133,734	91,0	0,37	
2	25,200	1686,064	9,0	0,84	
	Total	18819,798	100,0		

Figure S25. *t*-Butyl 3-acetoxy-3-(2-naphthyl)propanoate, **3g**.

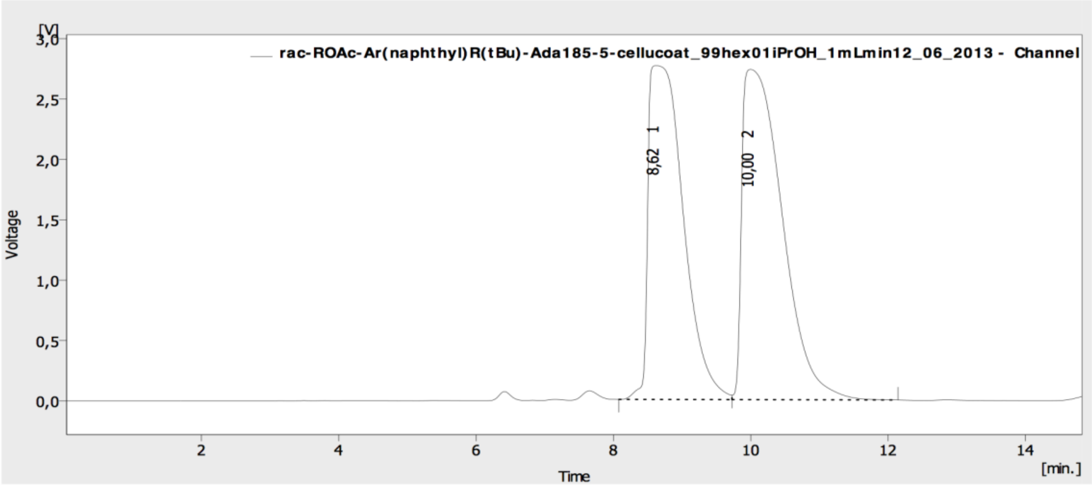

Result Table (Uncal - rac-ROAc-Ar(naphthyl)R(tBu)-Ada185-5-cellucoat_99hex01iPrOH_1mLmin12_06_2013 - Channel 1)

	Reten. Time [min]	Area [mV.s]	Area [%]	W05 [min]	Compound Name
1	8,617	98514,025	47,0	0,55	
2	9,997	111279,035	53,0	0,64	
	Total	209793,059	100,0		

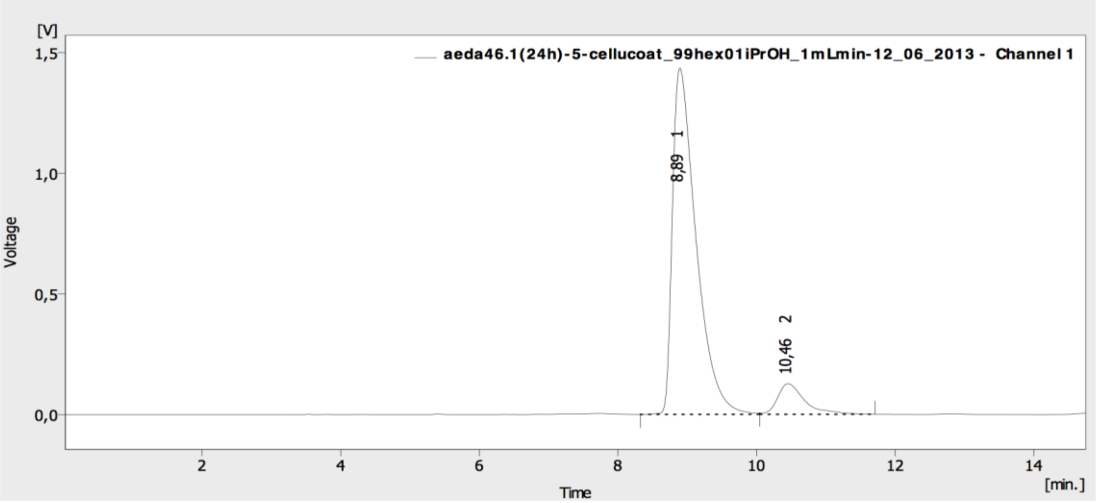

Result Table (Uncal - aeda46.1(24h)-5-cellucoat_99hex01iPrOH_1mLmin-12_06_2013 - Channel 1)

	Reten. Time [min]	Area [mV.s]	Area [%]	W05 [min]	Compound Name
1	8,893	34001,771	90,8	0,36	
2	10,457	3456,716	9,2	0,38	
	Total	37458,487	100,0		

Figure S26. *t*-Butyl 3-acetoxy-3-(4-chloro)propanoate, **3h**.

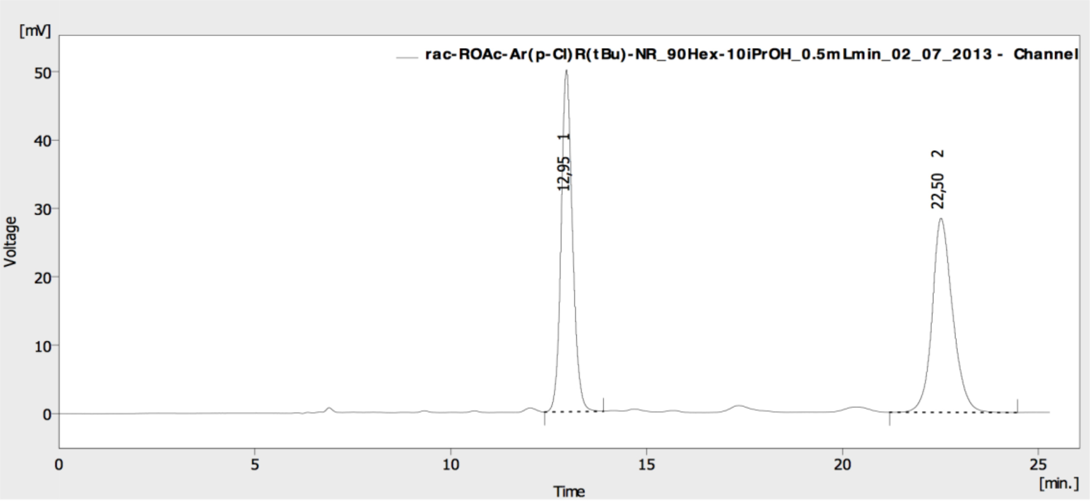

Result Table (Uncal - rac-ROAc-Ar(p-Cl)R(tBu)-NR_90Hex-10iPrOH_0.5mLmin_02_07_2013 - Channel 1)

	Reten. Time [min]	Area [mV.s]	Area [%]	W05 [min]	Compound Name
1	12,950	1006,742	49,3	0,30	
2	22,500	1034,691	50,7	0,54	
	Total	2041,433	100,0		

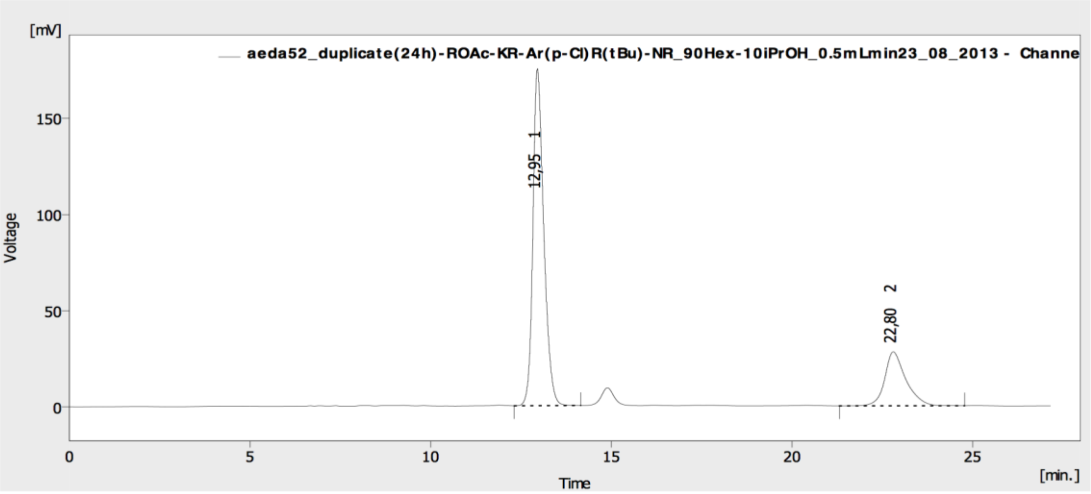

Result Table (Uncal - aeda52_duplicate(24h)-ROAc-KR-Ar(p-Cl)R(tBu)-NR_90Hex-10iPrOH_0.5mLmin23_08_2013 - Channel 1)

	Reten. Time [min]	Area [mV.s]	Area [%]	W05 [min]	Compound Name
1	12,950	3658,138	76,3	0,31	
2	22,800	1134,232	23,7	0,58	
	Total	4792,370	100,0		

Figure S27. *t*-Butyl 3-acetoxy-3-(2,6-dichlorophenyl)propanoate, **3i**.

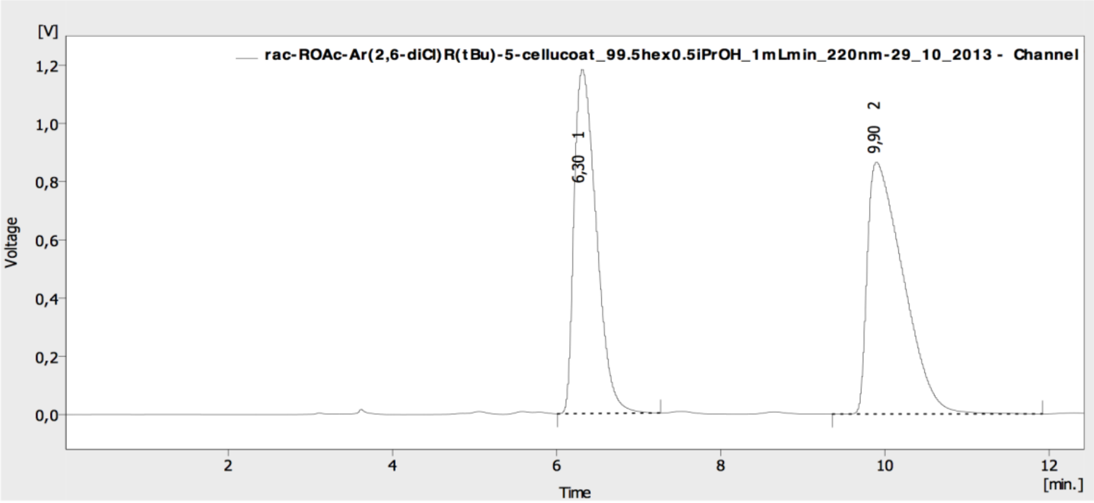

Result Table (Uncal - rac-ROAc-Ar(2,6-diCl)R(tBu)-5-cellucoat_99.5hex0.5iPrOH_1mLmin_220nm-29_10_2013 - Channel 1)

	Reten. Time [min]	Area [mV.s]	Area [%]	W05 [min]	Compound Name
1	6,300	22236,962	45,9	0,30	
2	9,900	26258,525	54,1	0,49	
	Total	48495,486	100,0		

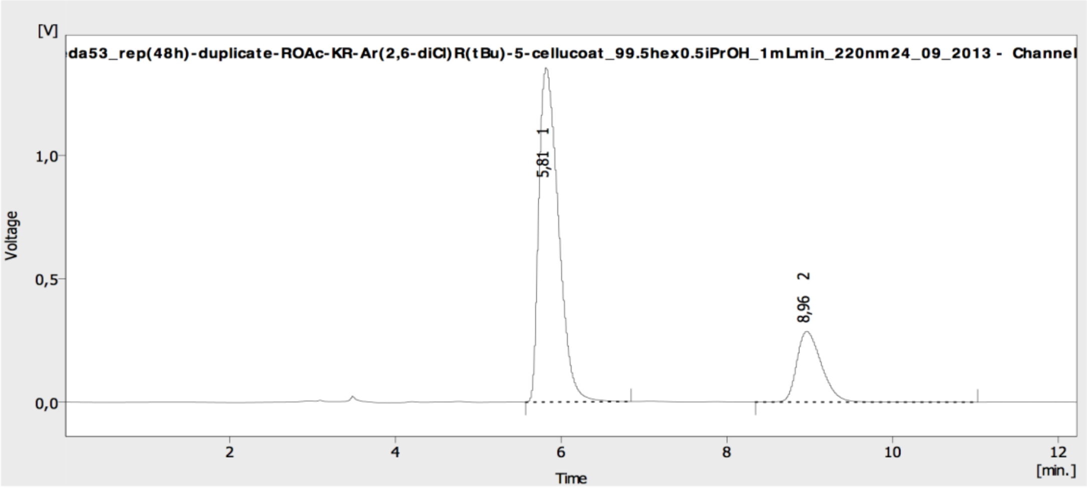

Result Table (Uncal - aeda53_rep(48h)-duplicate-ROAc-KR-Ar(2,6-diCl)R(tBu)-5-cellucoat_99.5hex0.5iPrOH_1mLmin_220nm24_09_2013 - Channel 1)

	Reten. Time [min]	Area [mV.s]	Area [%]	W05 [min]	Compound Name
1	5,810	22243,801	78,9	0,25	
2	8,960	5940,326	21,1	0,32	
	Total	28184,127	100,0		

Figure S28. 3-Hydroxy-*N*-methyl-3-phenylpropanamide, **4**.

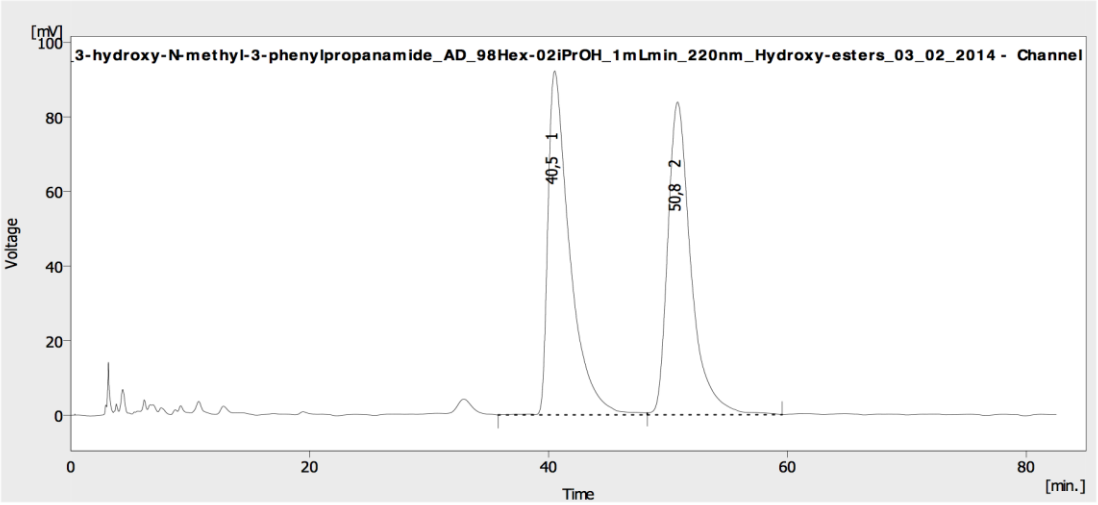

Result Table (Uncal - rac_3-hydroxy-N-methyl-3-phenylpropanamide_AD_98Hex-02iPrOH_1mLmin_220nm_Hydroxy-esters_03_02_2014 - Channel 1)

	Reten. Time [min]	Area [mV.s]	Area [%]	W05 [min]	Compound Name
1	40,500	11747,446	50,7	1,82	
2	50,800	11411,538	49,3	1,96	
	Total	23158,984	100,0		

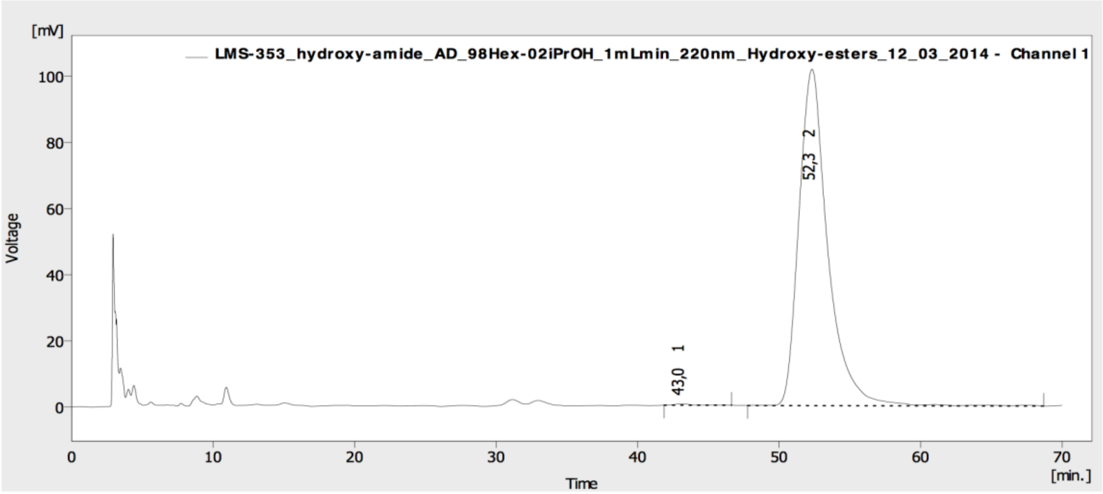

Result Table (Uncal - LMS-353_hydroxy-amide_AD_98Hex-02iPrOH_1mLmin_220nm_Hydroxy-esters_12_03_2014 - Channel 1)

	Reten. Time [min]	Area [mV.s]	Area [%]	W05 [min]	Compound Name
1	43,050	36,101	0,2	1,25	
2	52,317	14468,349	99,8	2,05	
	Total	14504,450	100,0		

Supplementary Section 3. NMR Spectra

The ^{1}H and $^{13}C\{^{1}H\}$ NMR spectra of the compounds (*S*)-**2a-i** and (*S*)-**4** are attached below.

Figure S29. Ethyl (*S*)-3-hydroxy-3-phenylpropanoate, (*S*)-**2a**.

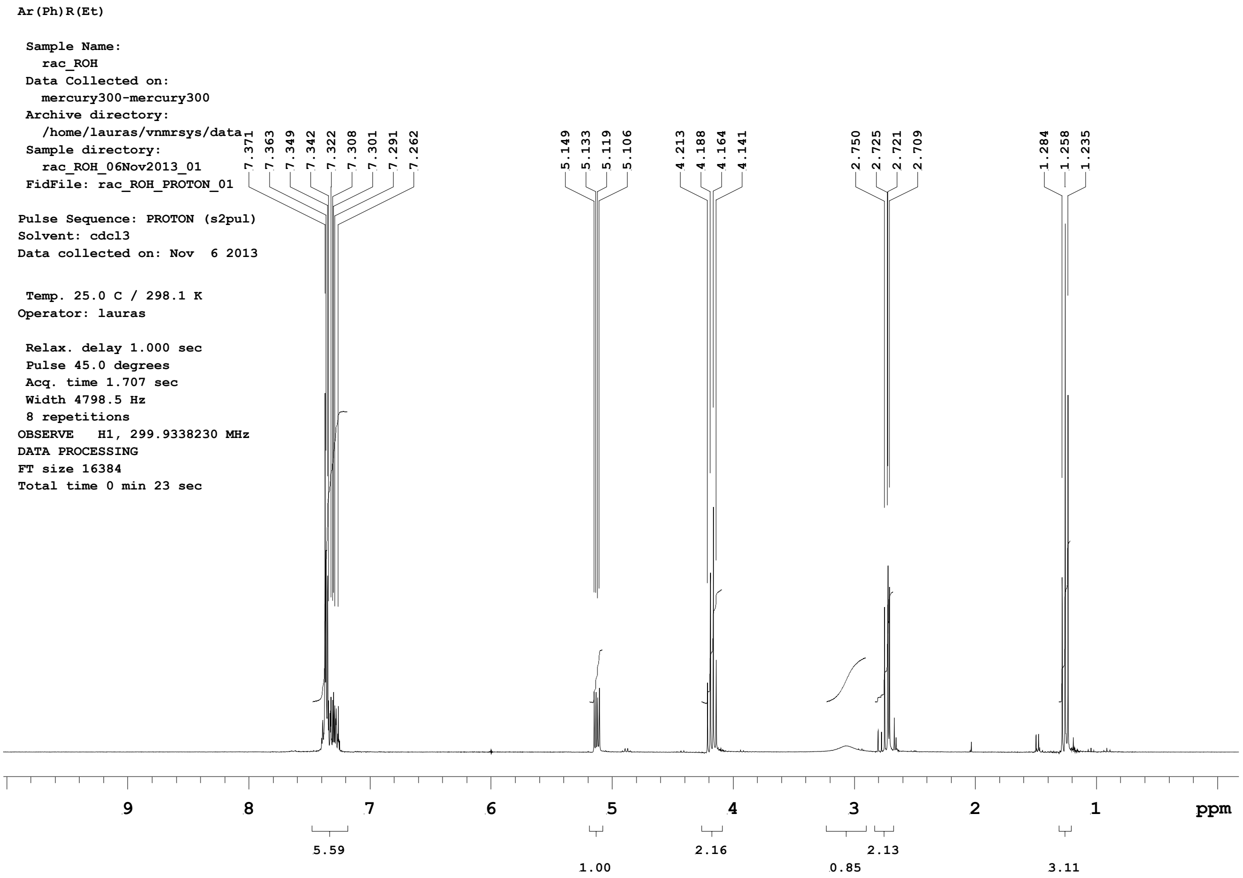

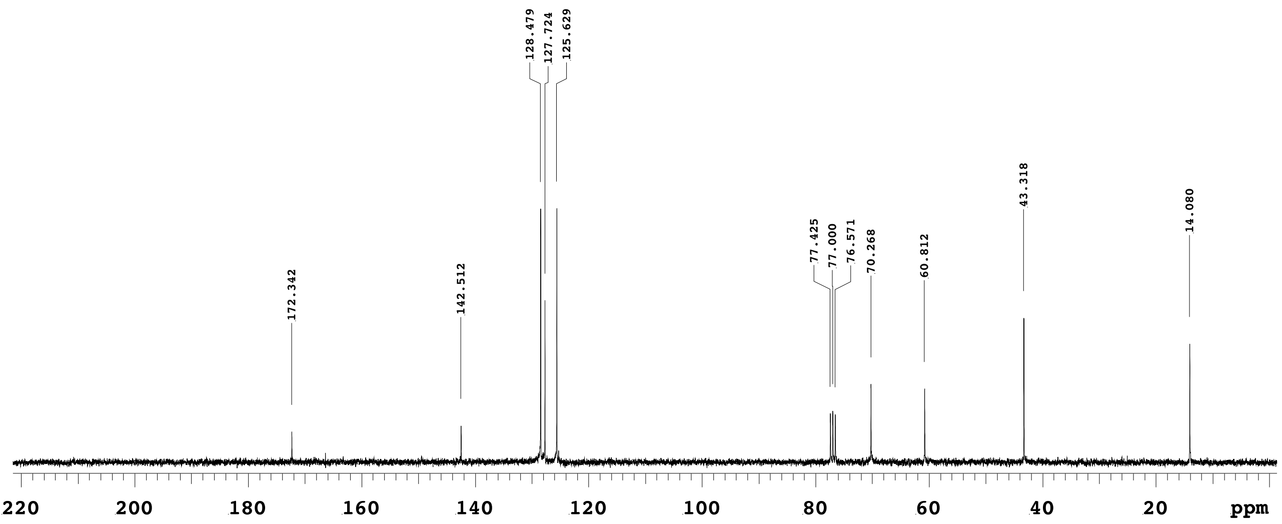

Figure S30. Ethyl (*S*)-3-hydroxy-3-(4-nitrophenyl)propionate, (*S*)-**2b**.

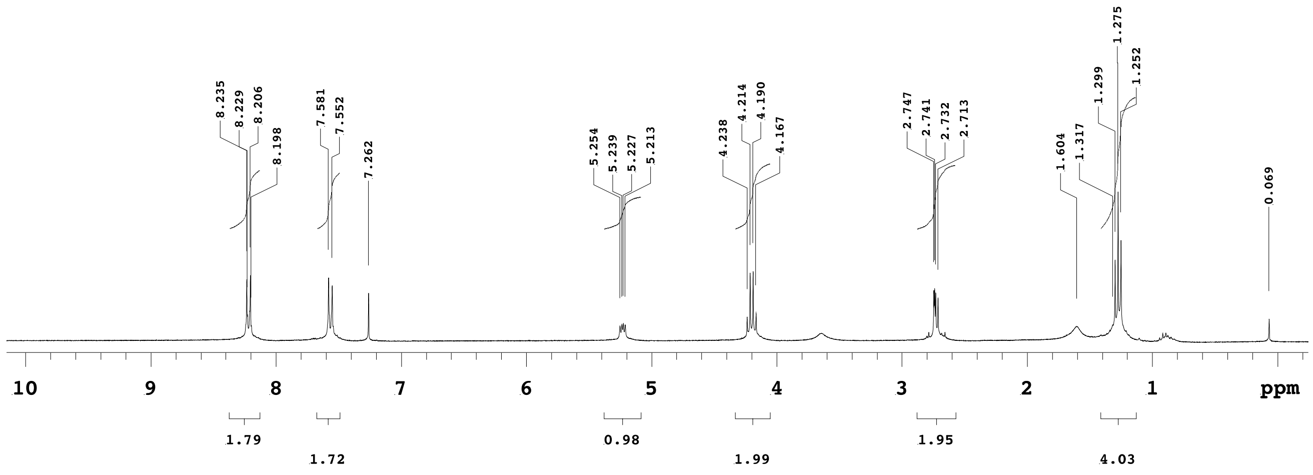

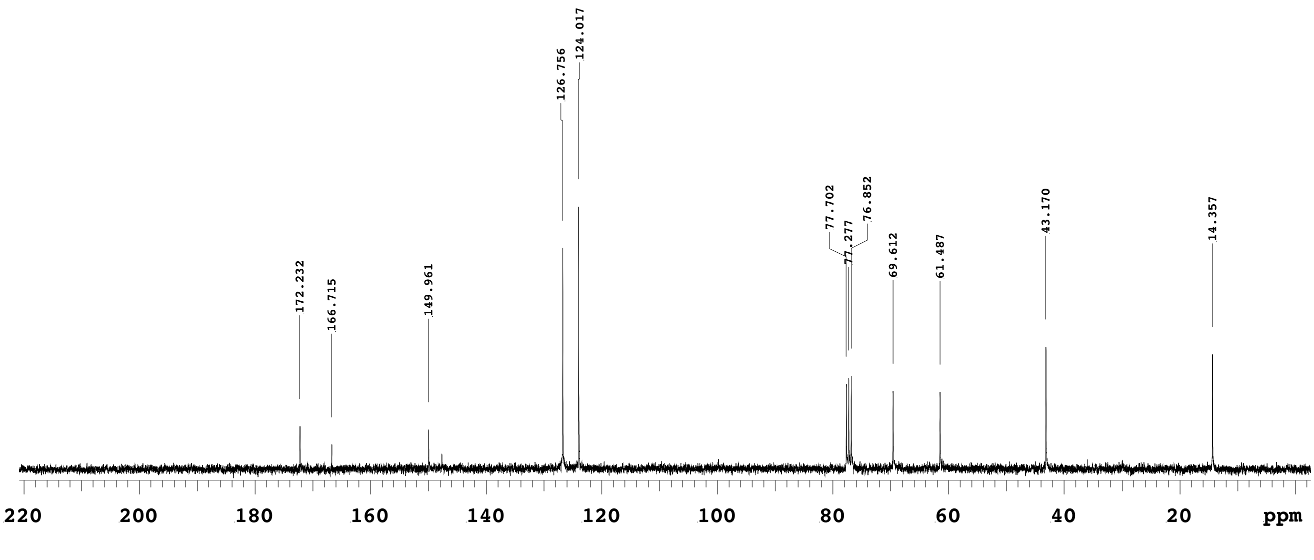

Figure S31. Ethyl (*S*)-3-hydroxy-3-(4-metoxyphenyl)propionate, (*S*)-**2c**.

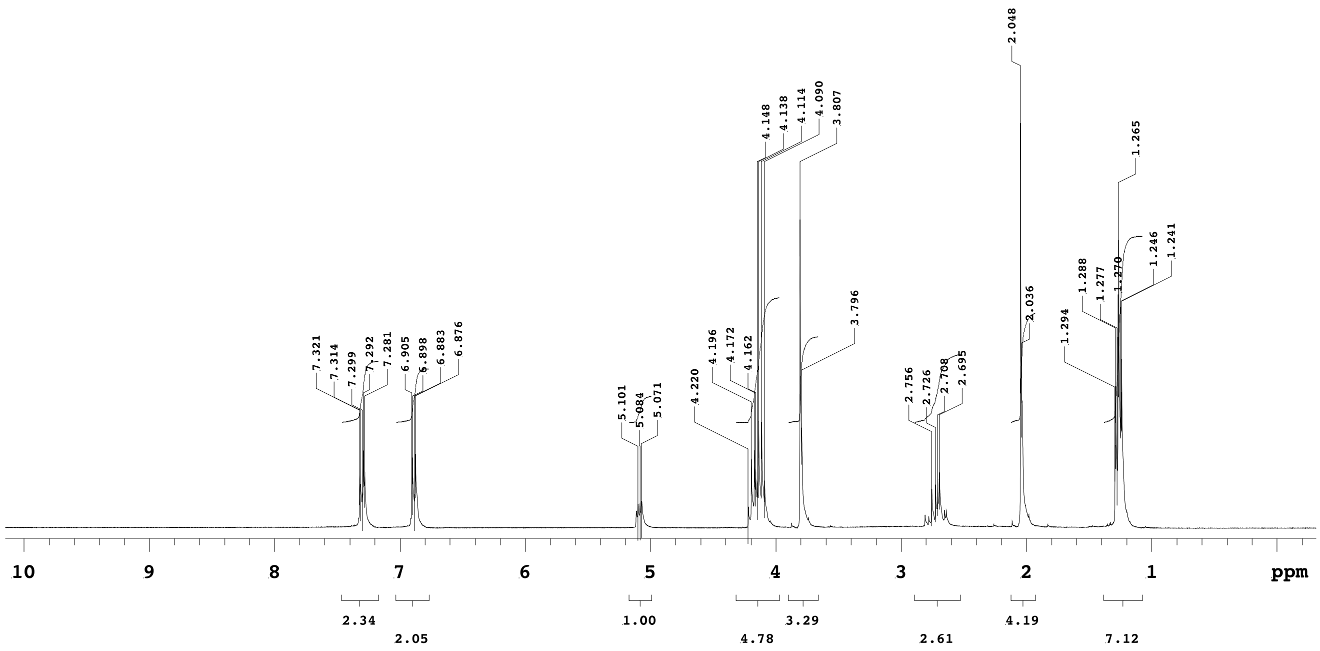

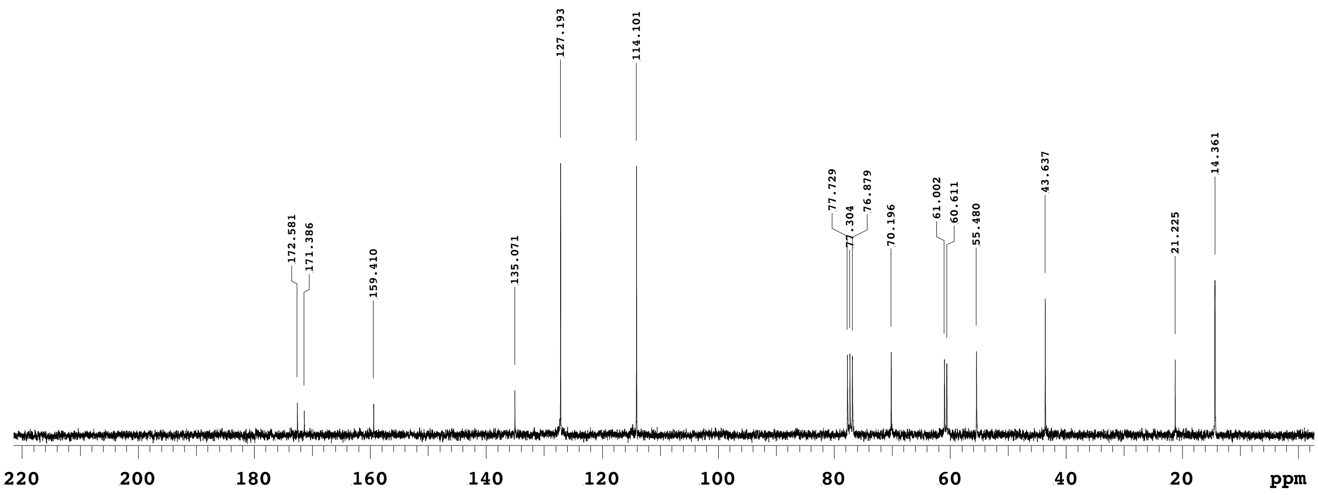

Figure S32. *t*-Butyl (*S*)-3-hydroxy-3-phenylpropanoate, (*S*)-**2d**.

Ar(Ph)R(tBu)

Sample Name:
rac_ROH_2
Data Collected on:
mercury300-mercury300
Archive directory:
/home/lauras/vnmrsys/data
Sample directory:
rac_ROH_2_06Nov2013_01
FidFile: rac_ROH_2_PROTON_01

Pulse Sequence: PROTON (s2pul)
Solvent: cdcl3
Data collected on: Nov 6 2013

Temp. 25.0 C / 298.1 K
Operator: lauras

Relax. delay 1.000 sec
Pulse 45.0 degrees
Acq. time 1.707 sec
Width 4798.5 Hz
8 repetitions
OBSERVE H1, 299.9338230 MHz
DATA PROCESSING
FT size 16384
Total time 0 min 23 sec

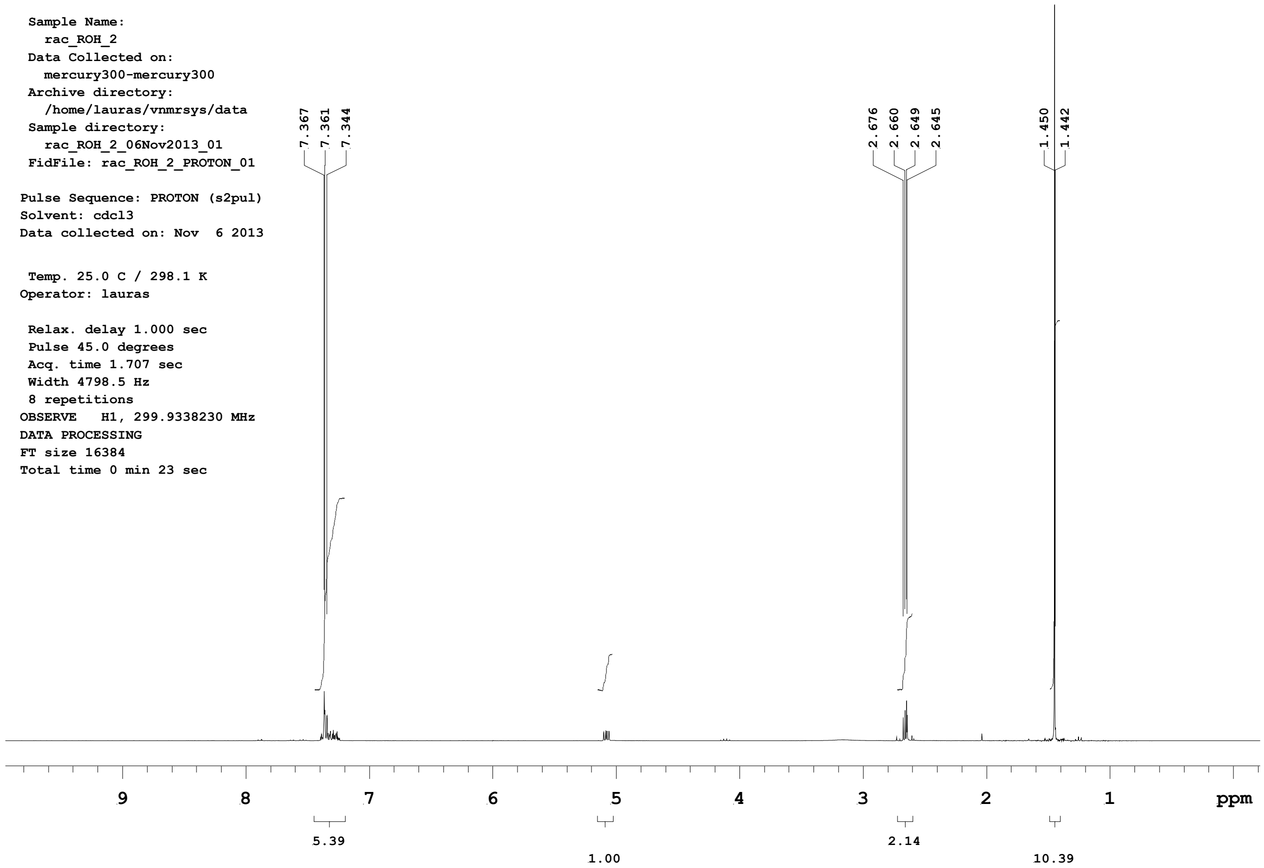

rac-ROH
Ar(Ph)R(tBu)

Sample Name:
KR_big_scale
Data Collected on:
mercury300-mercury300
Archive directory:
/home/lauras/vnmrsys/data
Sample directory:
KR_big_scale_26Nov2013_01
FidFile: KR_big_scale_CARBON_01

Pulse Sequence: CARBON (s2pul)
Solvent: cdcl3
Data collected on: Nov 26 2013

Temp. 25.0 C / 298.1 K
Operator: lauras

Relax. delay 1.000 sec
Pulse 45.0 degrees
Acq. time 0.868 sec
Width 18867.9 Hz
256 repetitions
OBSERVE C13, 75.4184198 MHz
DECOUPLE H1, 299.9352867 MHz
Power 39 dB
continuously on
WALTZ-16 modulated
DATA PROCESSING
Line broadening 0.5 Hz
FT size 32768
Total time 8 min 15 sec

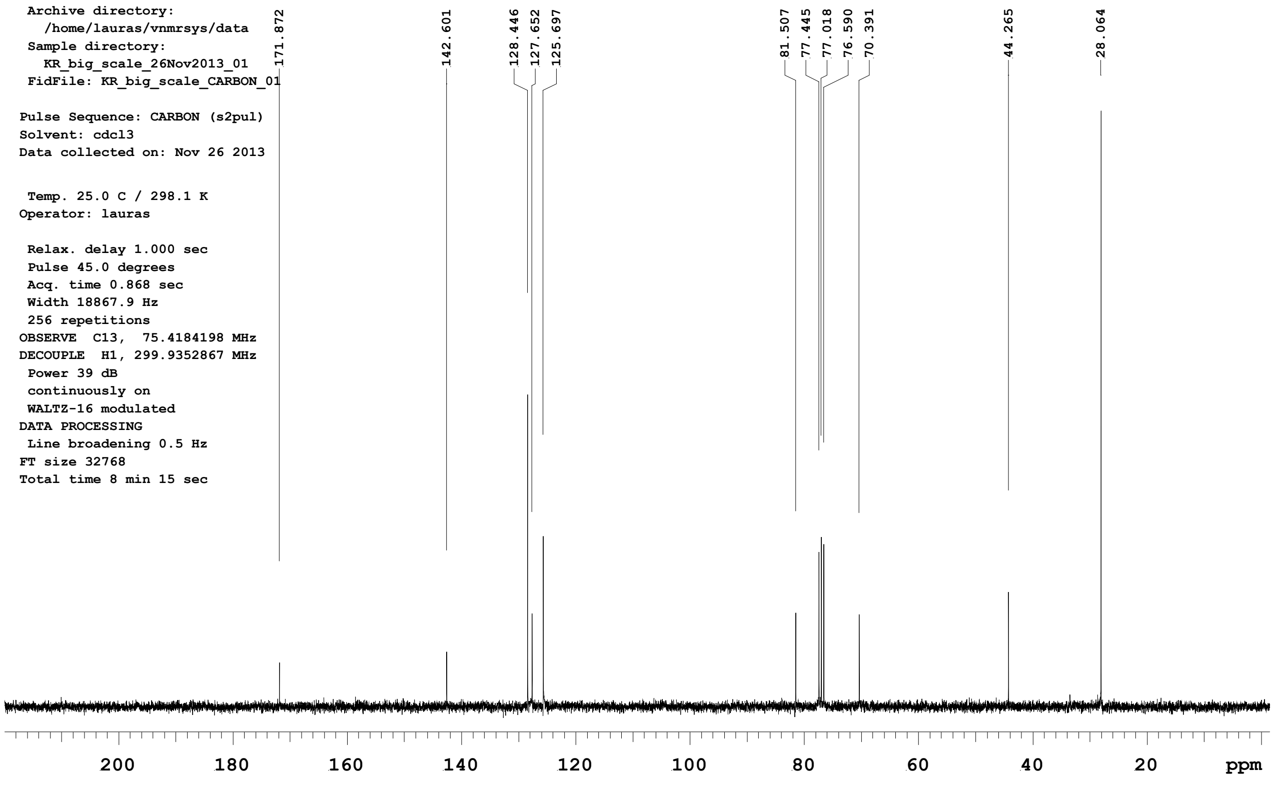

Figure S33. *t*-Butyl (*S*)-3-hydroxy-3-(4-nitrophenyl)propionate, (*S*)-**2e**.

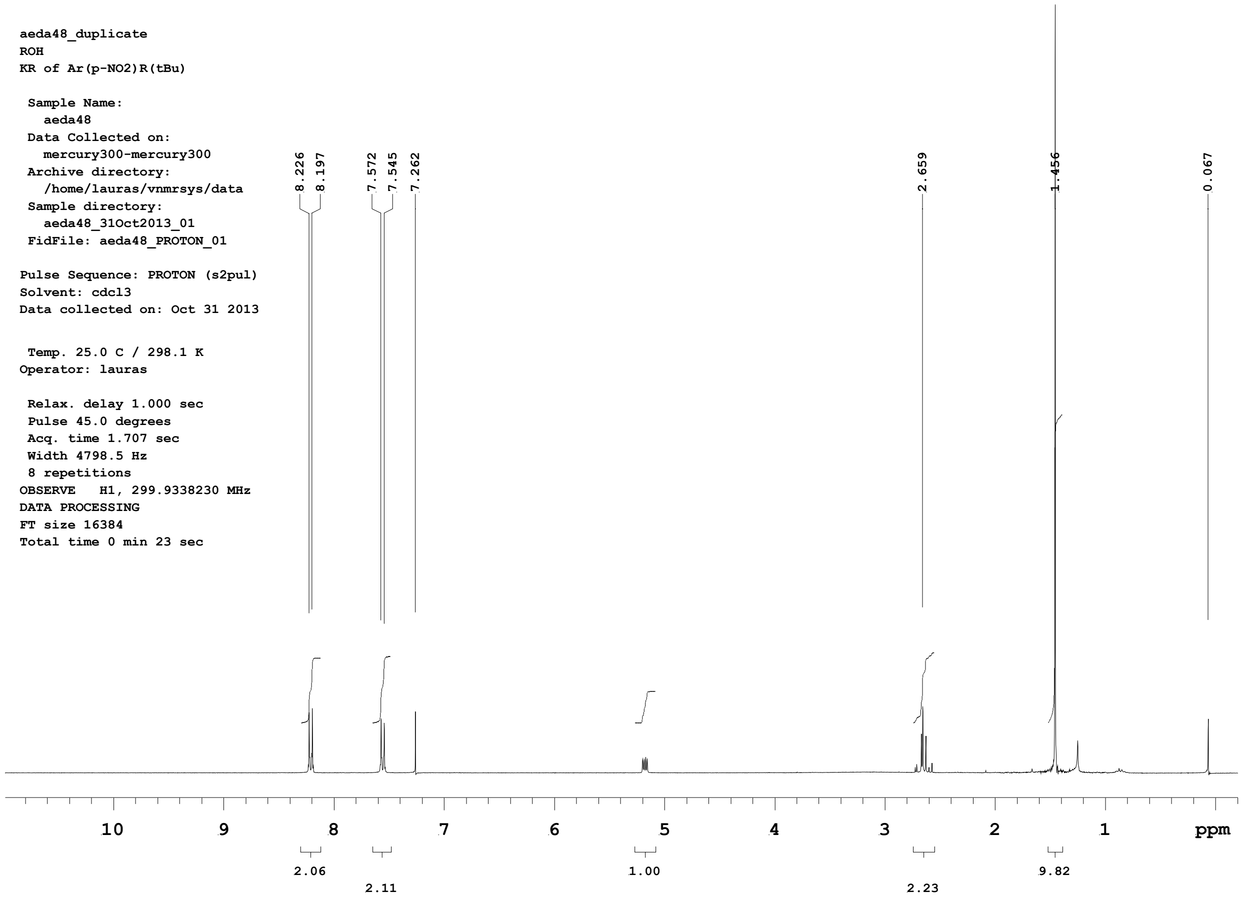

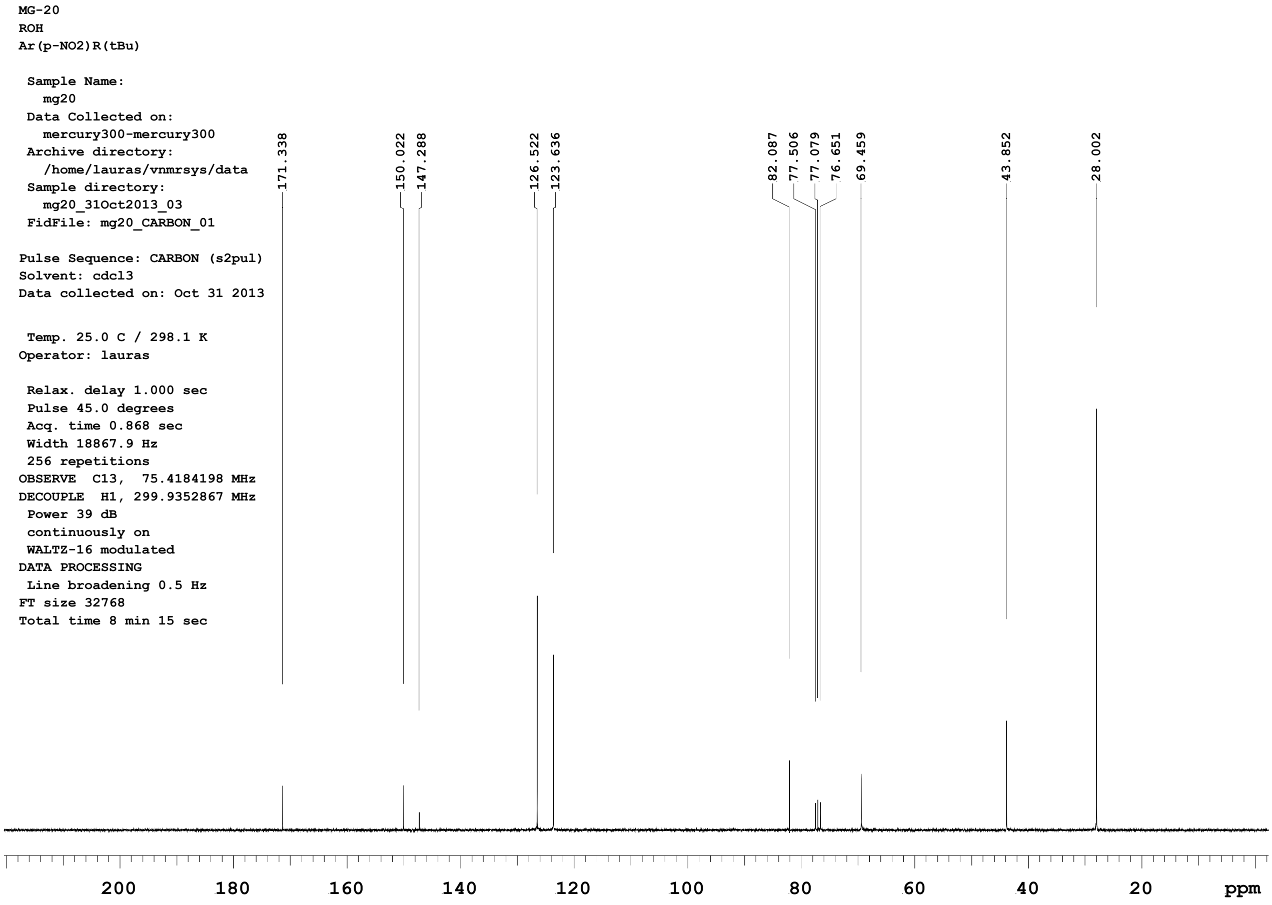

Figure S34. *t*-Butyl (*S*)-3-hydroxy-3-(4-metoxyphenyl)propionate, (*S*)-**2f**.

MG-14
rac-ROH
Ar(p-OMe)R(tBu)

Sample Name:
mg14
Data Collected on:
mercury300-mercury300
Archive directory:
/home/lauras/vnmrsys/data
Sample directory:
mg14_31Oct2013_01
FidFile: mg14_PROTON_01

Pulse Sequence: PROTON (s2pul)
Solvent: cdcl3
Data collected on: Oct 31 2013

Temp. 25.0 C / 298.1 K
Operator: lauras

Relax. delay 1.000 sec
Pulse 45.0 degrees
Acq. time 1.707 sec
Width 4798.5 Hz
8 repetitions
OBSERVE H1, 299.9338230 MHz
DATA PROCESSING
FT size 16384
Total time 0 min 23 sec

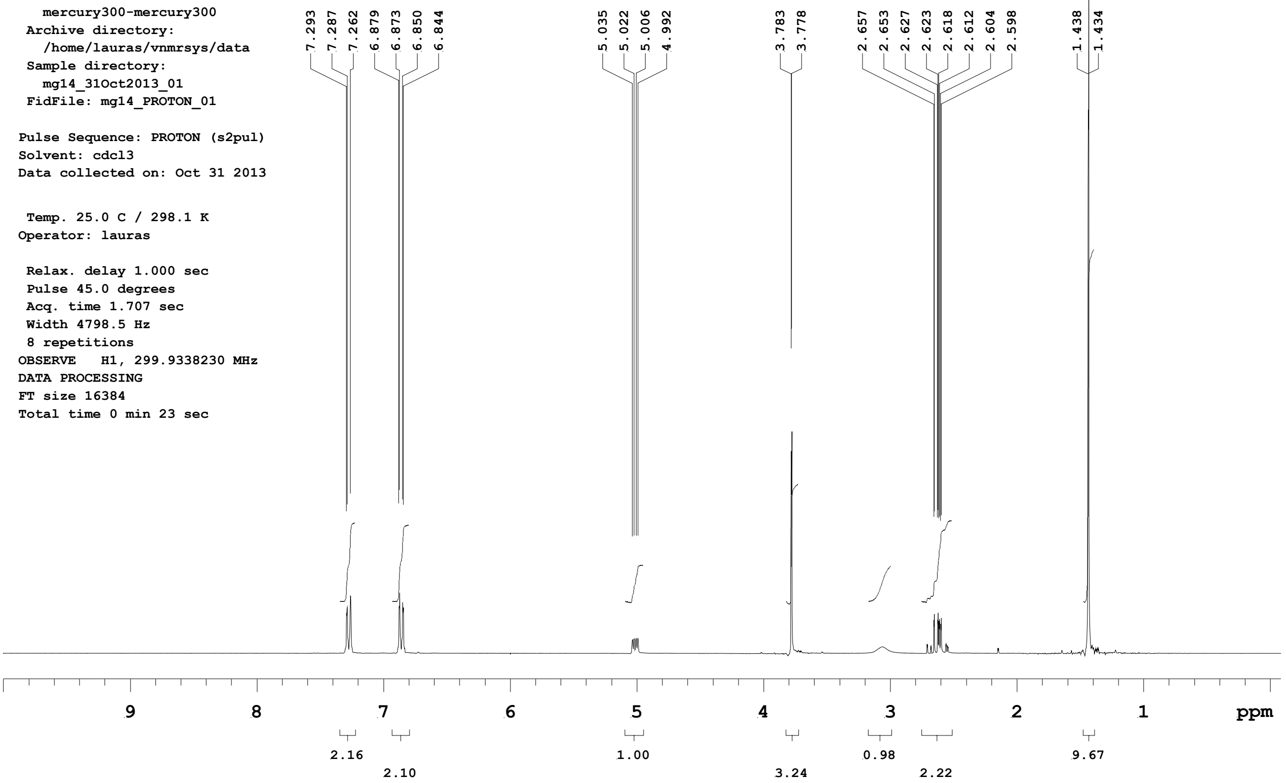

MG-14
rac-ROH
Ar(p-OMe)R(tBu)

Sample Name:
mg14
Data Collected on:
mercury300-mercury300
Archive directory:
/home/lauras/vnmrsys/data
Sample directory:
mg14_31Oct2013_01
FidFile: mg14_CARBON_01

Pulse Sequence: CARBON (s2pul)
Solvent: cdcl3
Data collected on: Oct 31 2013

Temp. 25.0 C / 298.1 K
Operator: lauras

Relax. delay 1.000 sec
Pulse 45.0 degrees
Acq. time 0.868 sec
Width 18867.9 Hz
256 repetitions
OBSERVE C13, 75.4184198 MHz
DECOUPLE H1, 299.9352867 MHz
Power 39 dB
continuously on
WALTZ-16 modulated
DATA PROCESSING
Line broadening 0.5 Hz
FT size 32768
Total time 8 min 15 sec

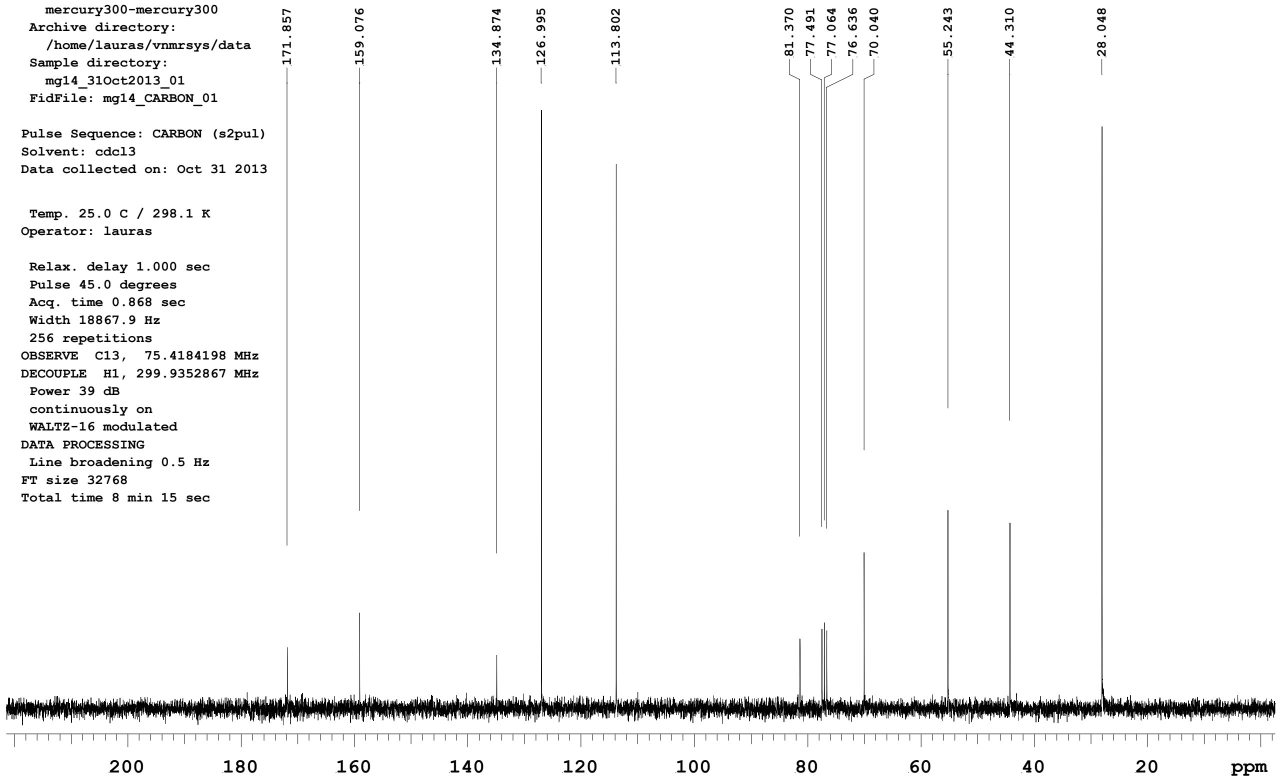

Figure S35. *t*-Butyl (*S*)-3-hydroxy-3-(2-naphthyl)propionate, (*S*)-**2g**.

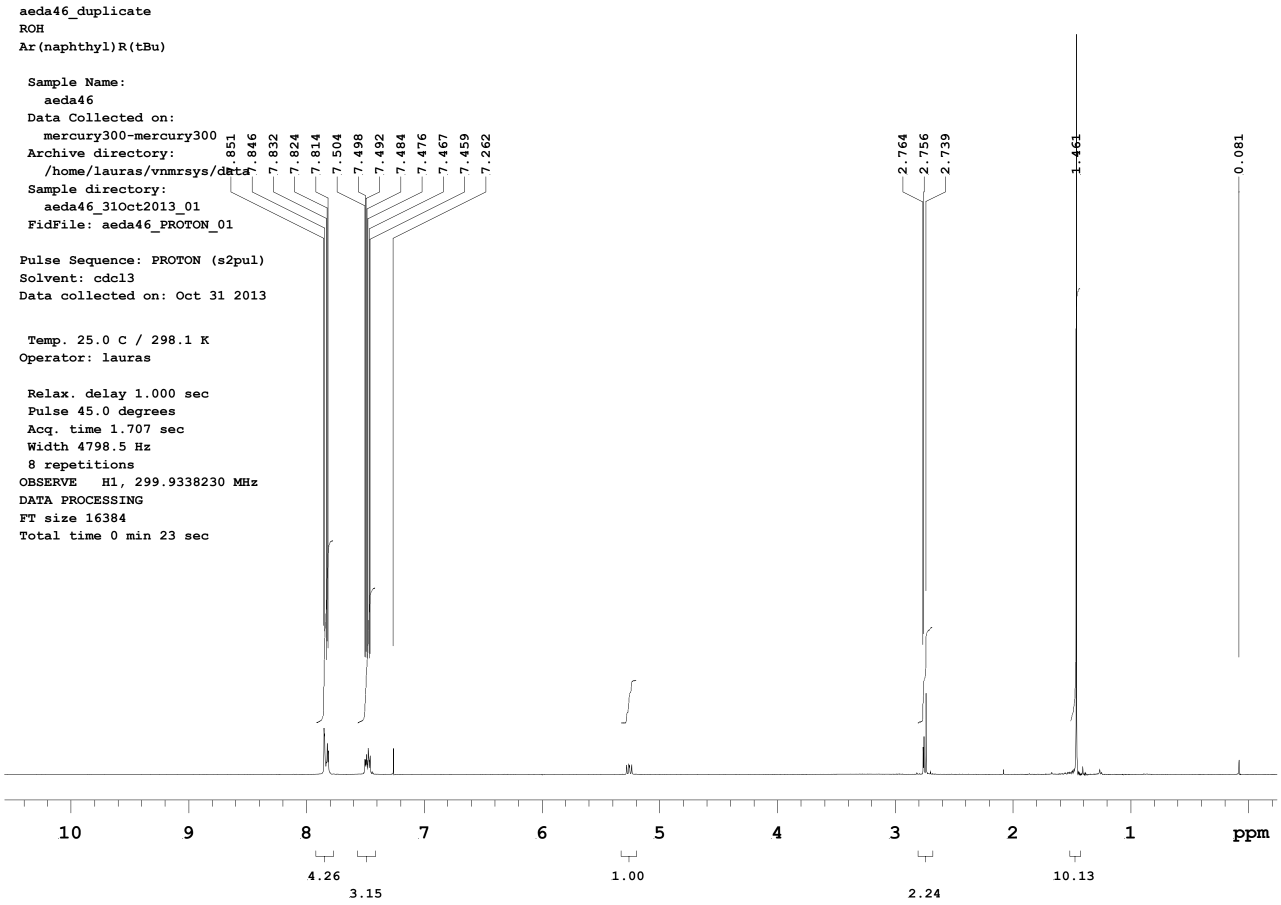

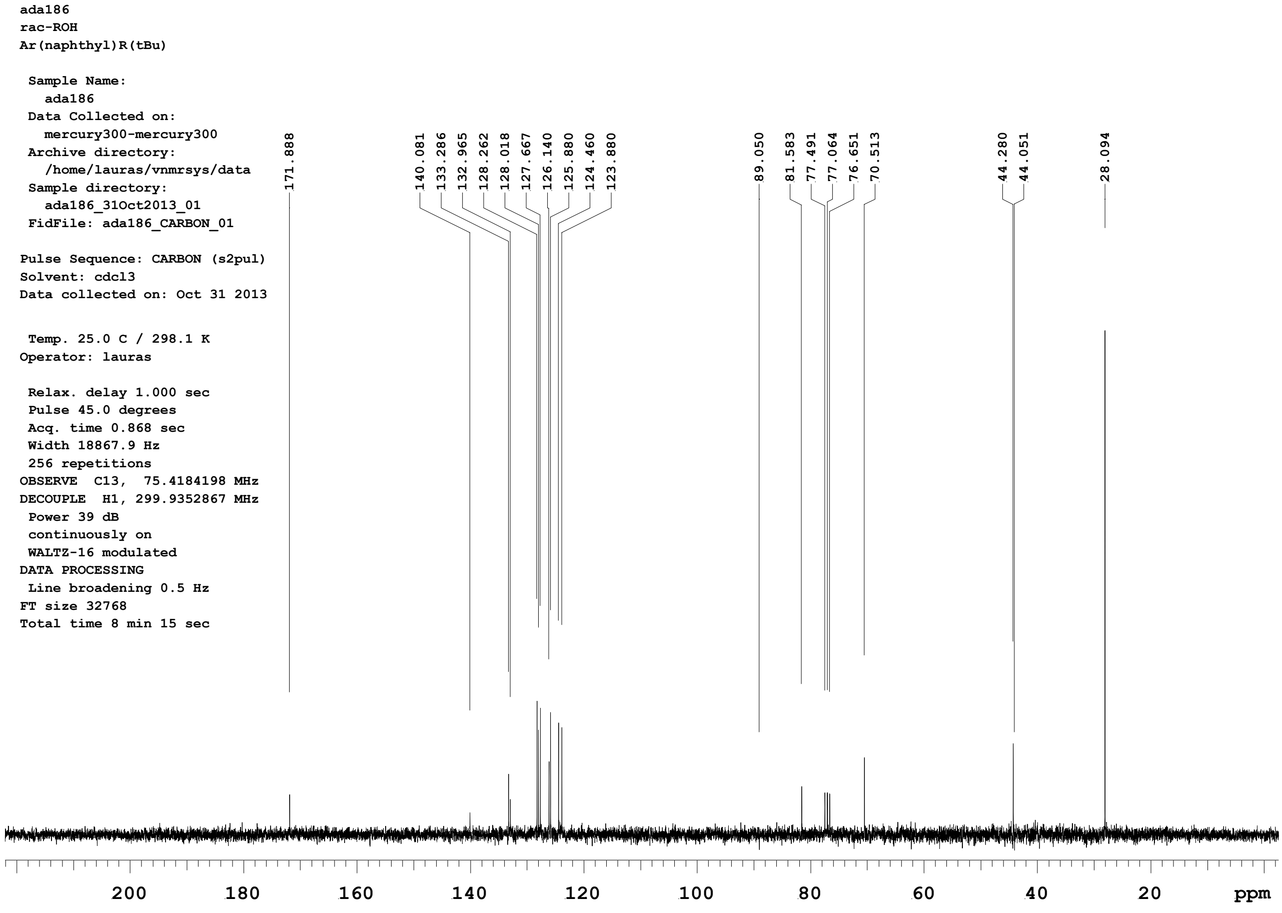

Figure S36. *t*-Butyl (*S*)-3-hydroxy-3-(4-chloro)propionate, (*S*)-**2h**.

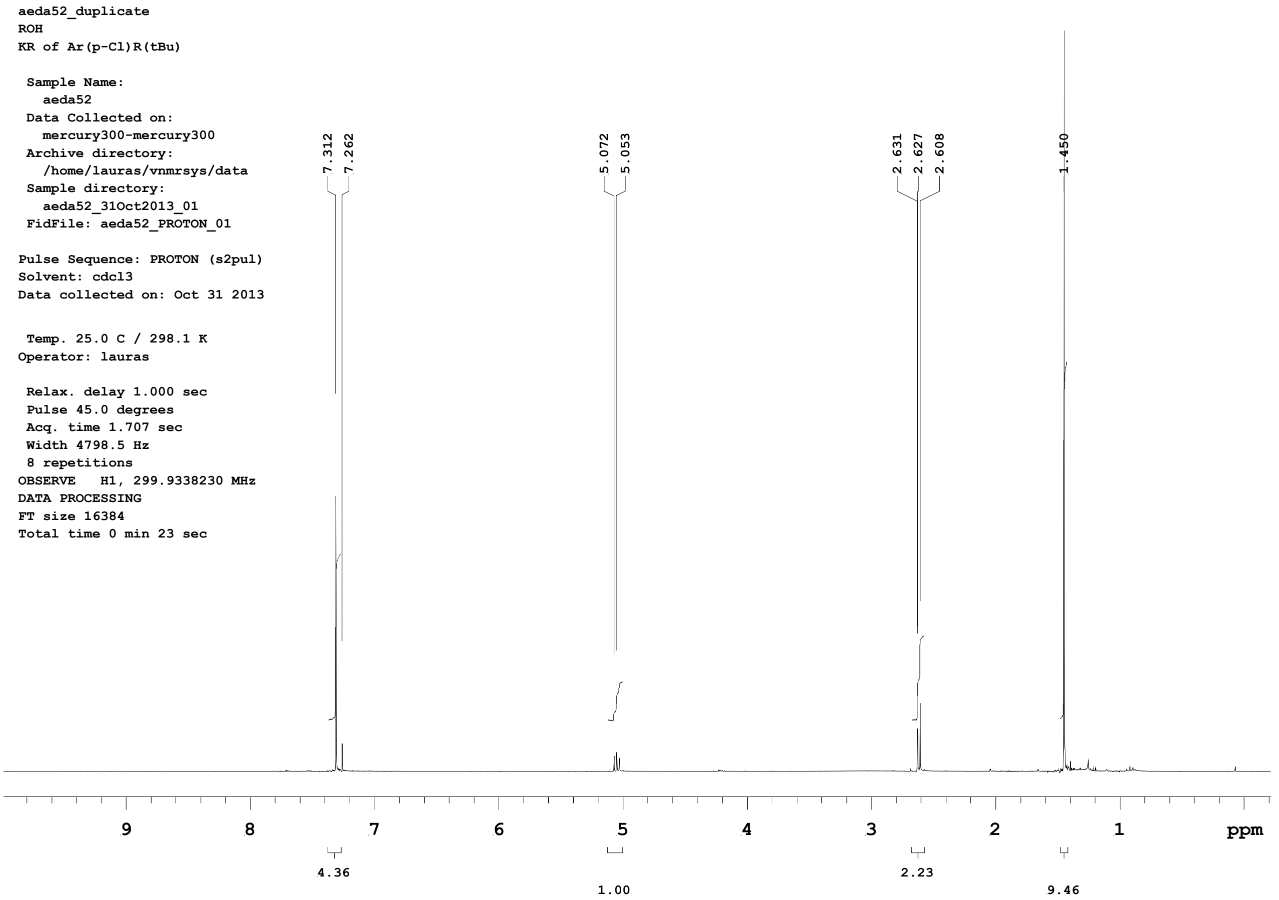

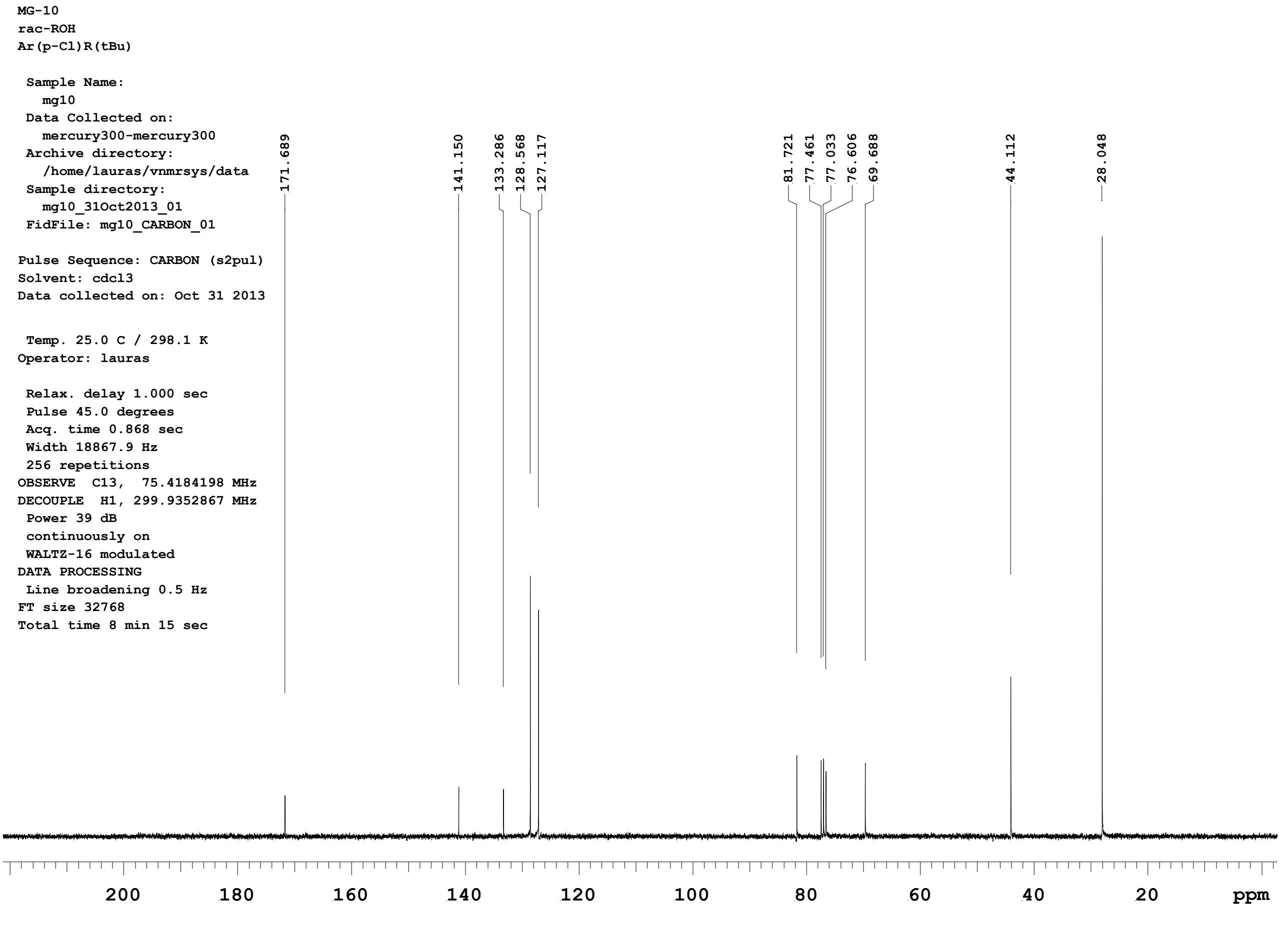

Figure S37. *t*-Butyl (*S*)-3-hydroxy-3-(2',4'-dichlorophenyl) propionate, (*S*)-**2i**.

aeda53_duplicate
ROH
KR of Ar(2,6-diCl)R(tBu)

Sample Name:
aeda53
Data Collected on:
mercury300-mercury300
Archive directory:
/home/lauras/vnmrsys/data
Sample directory:
aeda53_31Oct2013_01
FidFile: aeda53_PROTON_01

Pulse Sequence: PROTON (s2pul)
Solvent: cdcl3
Data collected on: Oct 31 2013

Temp. 25.0 C / 298.1 K
Operator: lauras

Relax. delay 1.000 sec
Pulse 45.0 degrees
Acq. time 1.707 sec
Width 4798.5 Hz
8 repetitions
OBSERVE H1, 299.9338230 MHz
DATA PROCESSING
FT size 16384
Total time 0 min 23 sec

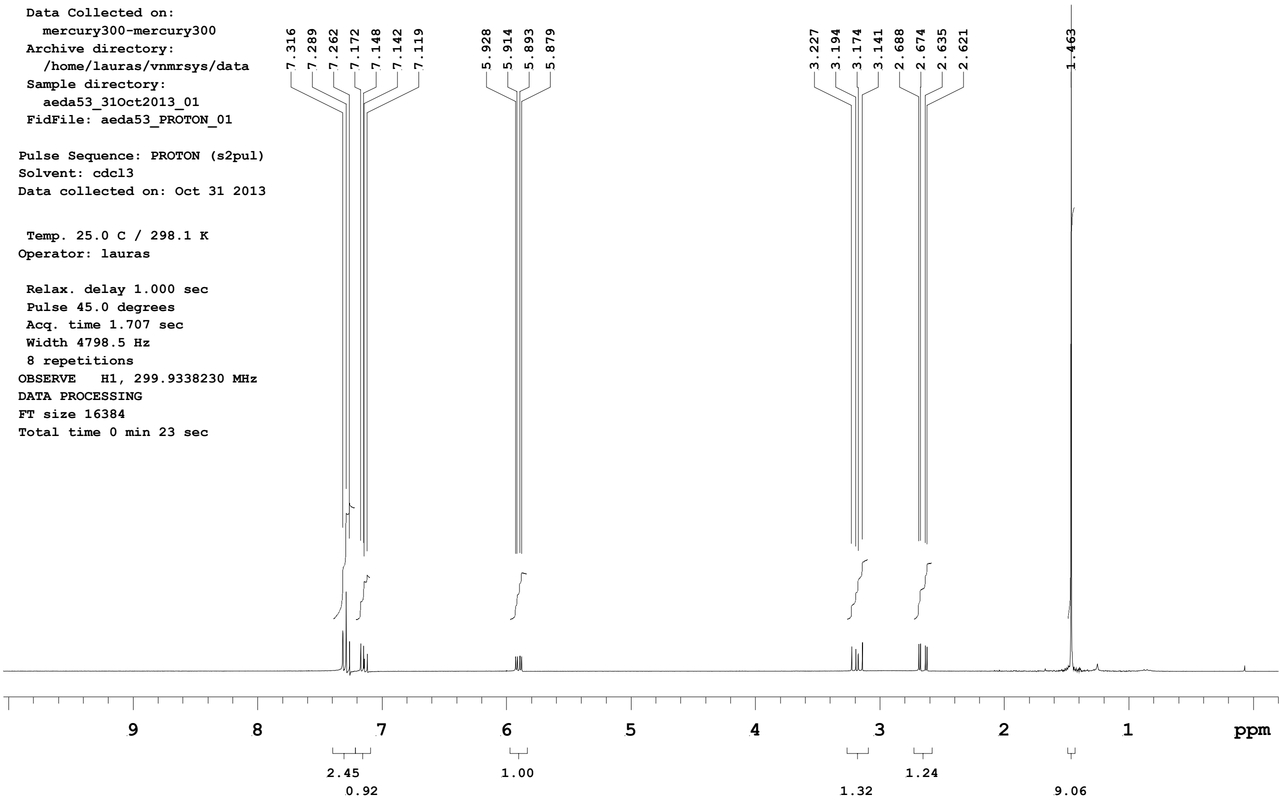

MG-15
ROH
Ar(2,6-diCl)R(tBu)

Sample Name:
mg15
Data Collected on:
mercury300-mercury300
Archive directory:
/home/lauras/vnmrsys/data
Sample directory:
mg15_31Oct2013_02
FidFile: mg15_CARBON_01

Pulse Sequence: CARBON (s2pul)
Solvent: cdcl3
Data collected on: Oct 31 2013

Temp. 25.0 C / 298.1 K
Operator: lauras

Relax. delay 0.500 sec
Pulse 45.0 degrees
Acq. time 0.868 sec
Width 18867.9 Hz
512 repetitions
OBSERVE C13, 75.4184198 MHz
DECOUPLE H1, 299.9352867 MHz
Power 39 dB
continuously on
WALTZ-16 modulated
DATA PROCESSING
Line broadening 0.5 Hz
FT size 32768
Total time 12 min

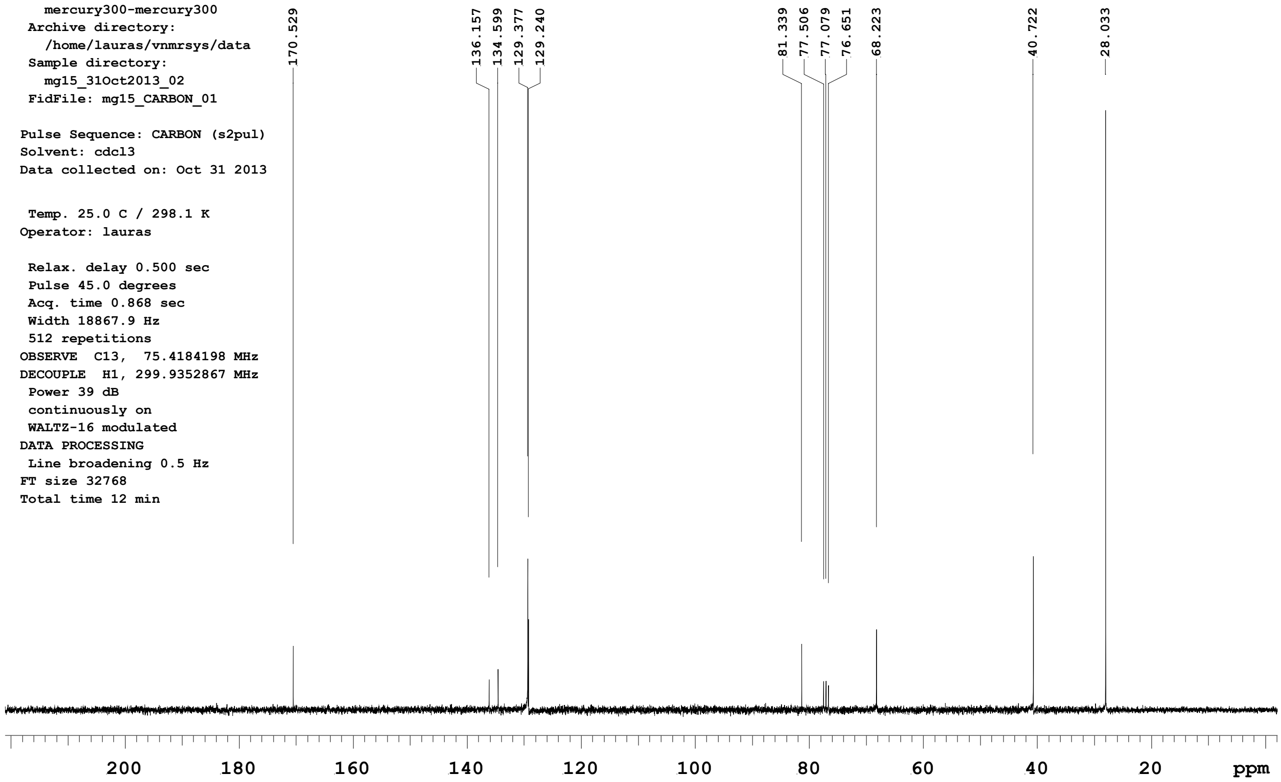

Figure S38. (*S*)-3-hydroxy-*N*-methyl-3-phenylpropanamide, (*S*)-**4**.

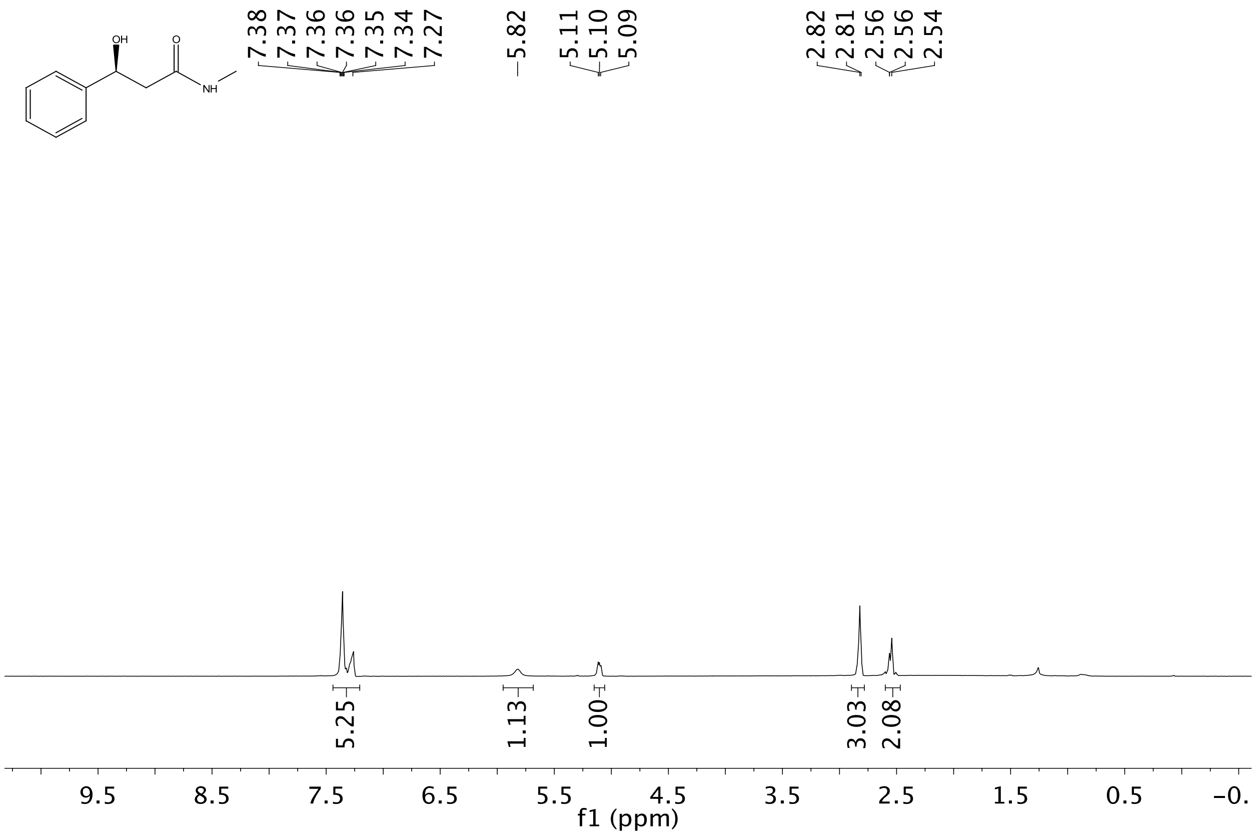

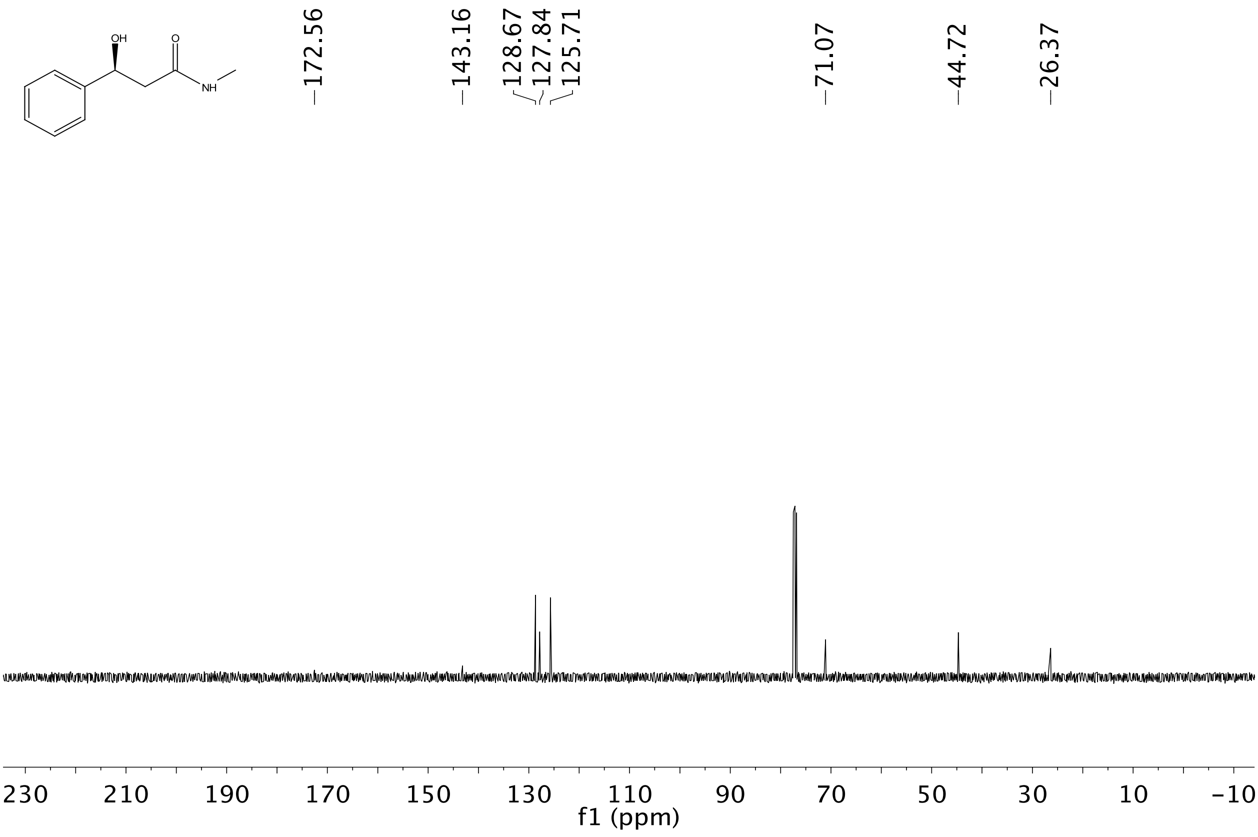

Supplement: Supplementary File 1 [file molecules-19-14273-s001.pdf]
